# Modeling differentiation-state transitions linked to therapeutic escape in triple-negative breast cancer

**DOI:** 10.1371/journal.pcbi.1006840

**Published:** 2019-03-11

**Authors:** Margaret P. Chapman, Tyler Risom, Anil J. Aswani, Ellen M. Langer, Rosalie C. Sears, Claire J. Tomlin

**Affiliations:** 1 Department of Electrical Engineering and Computer Sciences, University of California Berkeley, Berkeley, California, United States of America; 2 Department of Molecular and Medical Genetics, Oregon Health and Science University, Portland, Oregon, United States of America; 3 Department of Industrial Engineering and Operations Research, University of California Berkeley, Berkeley, California, United States of America; 4 Knight Cancer Institute, Oregon Health and Science University, Portland, Oregon, United States of America; 5 Center for Spatial Systems Biomedicine, Oregon Health and Science University, Portland, Oregon, United States of America; University of Pennsylvania, UNITED STATES

## Abstract

Drug resistance in breast cancer cell populations has been shown to arise through phenotypic transition of cancer cells to a drug-tolerant state, for example through epithelial-to-mesenchymal transition or transition to a cancer stem cell state. However, many breast tumors are a heterogeneous mixture of cell types with numerous epigenetic states in addition to stem-like and mesenchymal phenotypes, and the dynamic behavior of this heterogeneous mixture in response to drug treatment is not well-understood. Recently, we showed that plasticity between differentiation states, as identified with intracellular markers such as cytokeratins, is linked to resistance to specific targeted therapeutics. Understanding the dynamics of differentiation-state transitions in this context could facilitate the development of more effective treatments for cancers that exhibit phenotypic heterogeneity and plasticity. In this work, we develop computational models of a drug-treated, phenotypically heterogeneous triple-negative breast cancer (TNBC) cell line to elucidate the feasibility of differentiation-state transition as a mechanism for therapeutic escape in this tumor subtype. Specifically, we use modeling to predict the changes in differentiation-state transitions that underlie specific therapy-induced changes in differentiation-state marker expression that we recently observed in the HCC1143 cell line. We report several statistically significant therapy-induced changes in transition rates between basal, luminal, mesenchymal, and non-basal/non-luminal/non-mesenchymal differentiation states in HCC1143 cell populations. Moreover, we validate model predictions on cell division and cell death empirically, and we test our models on an independent data set. Overall, we demonstrate that changes in differentiation-state transition rates induced by targeted therapy can provoke distinct differentiation-state aggregations of drug-resistant cells, which may be fundamental to the design of improved therapeutic regimens for cancers with phenotypic heterogeneity.

## Author summary

Some classes of breast cancer tumors are composed of cells with different sets of observable traits, or phenotypes. The phenotype corresponds to particular cellular functionality and can arise due to the genetic/epigenetic code inside the cell, the environment outside the cell, and the genotype-environment interaction. Interestingly, treating a population of cancer cells with specific targeted therapies can stimulate changes in the phenotypic make-up of the population, contributing to resistance against the drug. Previous studies have indicated that changes in phenotypic composition of cancer cell populations might be caused by cells transitioning between phenotypes, but details of the transitions are not well-understood due to lack of sufficient time series data. Using a novel data set with well-established numerical methods, the results presented here improve our understanding of the phenotypic transitions occurring between drug-treated triple-negative breast cancer cells and have the potential to inform the design of improved cancer treatment strategies.

## Introduction

Heterogeneity of phenotypic states in cancer cell populations is likely driven by both genetic [1] [2] [3] and epigenetic [4] [5] [3] mechanisms, and is linked to the aggressiveness of cancer and its response to therapy. In particular, different phenotypic states of breast cancer cells within a tumor are associated with increased tumorigenic and metastatic capacity [6] [7], differential sensitivity to chemotherapy [8], and the development of drug resistance [7] [9] [4] [5]. There is growing evidence that dynamic interactions between phenotypic states occur in cancer cell populations, such as cells transitioning from one phenotypic state to another. Cancer stem cells, a small subset of cancer cells hypothesized to drive tumorigenesis, were initially implicated as a primary source of phenotypic heterogeneity, since they differentiate generating daughter cells with diverse phenotypic traits [10] [11]. This hierarchical explanation for phenotypic heterogeneity, however, does not necessarily agree with more recent empirical studies, which suggest that cell-state transition can occur more generally between several types of cancer cells, both stem and non-stem. For example, breast cancer stem-like cells were determined to arise *de novo* from non-stem-like basal and luminal cells using a Markov model and empirical validation [12], and sequencing of breast cancer stem cell populations demonstrated the existence of bidirectional transition between cancer stem cells and differentiated tumor cells [13]. Moreover, the same four epithelial differentiation states (two luminal phenotypes and two basal phenotypes) were identified in normal human breast tissues and in human breast cancer tissues, though in altered proportions [14], indicating that the phenotypic states of some epithelial cells switch to different states after the onset of the disease.

Phenotypic-state transition can also play a major role in the development of drug resistance in cancer cell populations, implicating such dynamic behavior as a therapeutic escape mechanism. The chemotherapy Adriamycin was found to prompt epithelial-to-mesenchymal transition (EMT) and apoptosis depending on cell cycle in the human breast adenocarcinoma cell line MCF7, but only transitioning cells exhibited multi-drug resistance and enhanced invasive potential [15]. Resistance to HER2-targeted therapies was discovered following spontaneous EMT in HER2^+^ luminal breast cancer [16]. Interestingly, treating HER2^+^ PTEN^-^ breast cancer cells continually with the HER2-targeting antibody Trastuzumab was observed to induce EMT, convert the disease to a triple-negative breast cancer, increase cancer stem cell frequency, and enhance metastatic potential [17]. Importantly, some studies have shown that such phenotypic transitions can be reversible, indicating that a better understanding of plasticity might suggest how to trap or drive cells into a state vulnerable to treatment. For example, one study that examined several drug-sensitive cancer cell lines in response to anti-cancer therapies (e.g., non-small cell lung cancer cell line PC9 treated with Erlotinib) repeatedly found a small fraction of cells occupying a reversible drug-tolerant state [5]. In addition, treating breast cancer cells with a taxane was shown to bring about transition to a transient CD44^hi^CD24^hi^ chemotherapy-tolerant state, and administering a sequence of anti-cancer agents was able to weaken this resistance [9].

In parallel with empirical work, computational models have been built to examine phenotypic-state dynamics in cancer cell populations and the role of these dynamics in the development of drug resistance [9] [12] [18] [19] [20] [21] [22] [23] [24]. A Markov chain model predicted that cancer stem-like cells can arise from non-stem-like cells using probabilities identified from observations at two time points [12]. Although parameter estimation error was not examined, the prediction was validated in an experiment [12]. Another pivotal study used ordinary differential equation (ODE) modeling to predict that cells expressing a transient drug-tolerant phenotype arise from non-stem-like cells [9]. While the model itself was not tested on independent data, the prediction deduced from the model was validated empirically [9]. Further, an ODE model was developed using the principles of biochemical reactions to represent cell-state birth, death, and transition [21] [22]. A dynamical model that generalized prior cell-state transition models [12] [21] [22] was constructed using a Markov process with a finite number of cell divisions [23], and phenotypic-state equilibria and stability properties were studied [23]. In the related field of clonal tumor evolution, a stochastic genotypic-state birth-death process model with mutations and a corresponding deterministic ODE model were developed [20]. The models along with Monte Carlo sampling and observations at two time points informed parameter sensitivity analysis, a treatment window approximation, and investigations of therapeutic scheduling [20]. Although our first modeling effort in the HCC1143 cell line of basal, mesenchymal, and non-basal/non-mesenchymal states included estimation of parameter variabilities, the training data set was small for the number of parameters that required identification, and no statistically significant drug-induced effects on phenotypic-state transitions were detected [19]. Studies with cell-state dynamical models rarely include statistical analysis of model parameters (refs. [19] and [20] are exceptions) because the available data often lacks sufficient quality and quantity at multiple time points. However, in the current paper, we leverage novel data sets to estimate model parameter variations, infer statistically significant drug-induced effects on phenotypic-state transitions, and test model generalizability.

In our recent work, we performed a large-scale phenotypic profiling study of triple-negative breast cancers exposed to a library of targeted therapeutics [18]. This study demonstrated that some targeted therapies affect the frequencies of luminal, basal, and mesenchymal states in heterogeneous triple-negative breast cancer cell lines, aggregating cells into particular drug-tolerant differentiation states [18]. The aggregated state identity was found to depend on the therapeutic target [18]. MEK and PI3K/mTOR inhibitors exemplified this effect, aggregating cells into distinct basal-differentiated and luminal-differentiated drug-tolerant persister states, respectively [18]. Using quantitative models of two states (basal, non-basal), we verified experimental evidence suggesting that these differentiation-state aggregations occur through phenotypic-state transition rather than Darwinian selection of pre-existing basal or non-basal cells [18].

However, these basal-specific models do not provide insights into the behaviors of mesenchymal-differentiated or luminal-differentiated breast cancer cells. Improved understanding of the dynamic nature of basal, mesenchymal, luminal, and non-basal/non-mesenchymal/non-luminal tumor cell states is needed to advance patient-specific clinical treatment of breast cancer. Specifically, the first three states predominate “basal-like” triple-negative tumors, “claudin-low” triple-negative tumors, and “luminal” ER^+^ tumors respectively [25] [26] [6], and many triple-negative tumors harbor a heterogeneous mixture of cells occupying all four states [18] [27]. This paper undertakes the important problem of examining the feasibility of transitions between any two of the four key differentiation states in triple-negative breast cancer cell populations under different treatment conditions.

To address this problem, we leverage two time series data sets of HCC1143-derived cell populations from Risom et al. that were acquired in two experiments conducted about one year apart [18]. Each data set contains numbers of cells occupying each differentiation state and numbers of cells where the dying cells are also specified following a particular treatment. There were four different treatment conditions: 1*μ*M Trametinib (MEK inhibitor), 1*μ*M BEZ235 (PI3K/mTOR inhibitor), 1*μ*M Trametinib+1*μ*M BEZ235 (equal-ratio combination), and DMSO (baseline).

The specific purpose of this paper is to develop and justify quantitative dynamic models of basal, mesenchymal, luminal, and non-basal/non-mesenchymal/non-luminal (DSNS for “differentiation-state non-specified”) states and to examine how different treatment conditions affect the dynamics of these four differentiation states in the HCC1143 cell line. We use our models to infer new biological insights: 1) how often HCC1143-derived cells may transition between any two of the four differentiation states following treatment with therapy or DMSO, 2) the statistical significance or insignificance of therapy-induced differences in the transition rates, and 3) how changes in transition rates may underlie certain differentiation-state aggregations of drug-tolerant cells reported in [18]. Taken together, these insights demonstrate the feasibility of transitions in the context of the four key differentiation states in triple-negative breast cancer and how different treatments can distinctly affect the behaviors of these transitions.

Our computational models are novel in particular because they were trained on an unprecedented amount of HCC1143 time series data using well-established numerical methods, specifically alternating minimization [28] wrapped around convex optimization [29]. Further, we evaluated our models on test data that was collected in a separate experiment from the training data, and we estimated variations of the model parameters due to measurement noise (via resampling residuals “wild” bootstrap [30]) to detect statistically significant effects. Notably, we leverage our models to predict how differentiation-state transitions change in response to targeted or combined therapy and to infer how these changes are linked to therapeutic escape in triple-negative breast cancer cell populations.

## Results

### Drug-specific differentiation-state dynamic models

**Fig 1:**
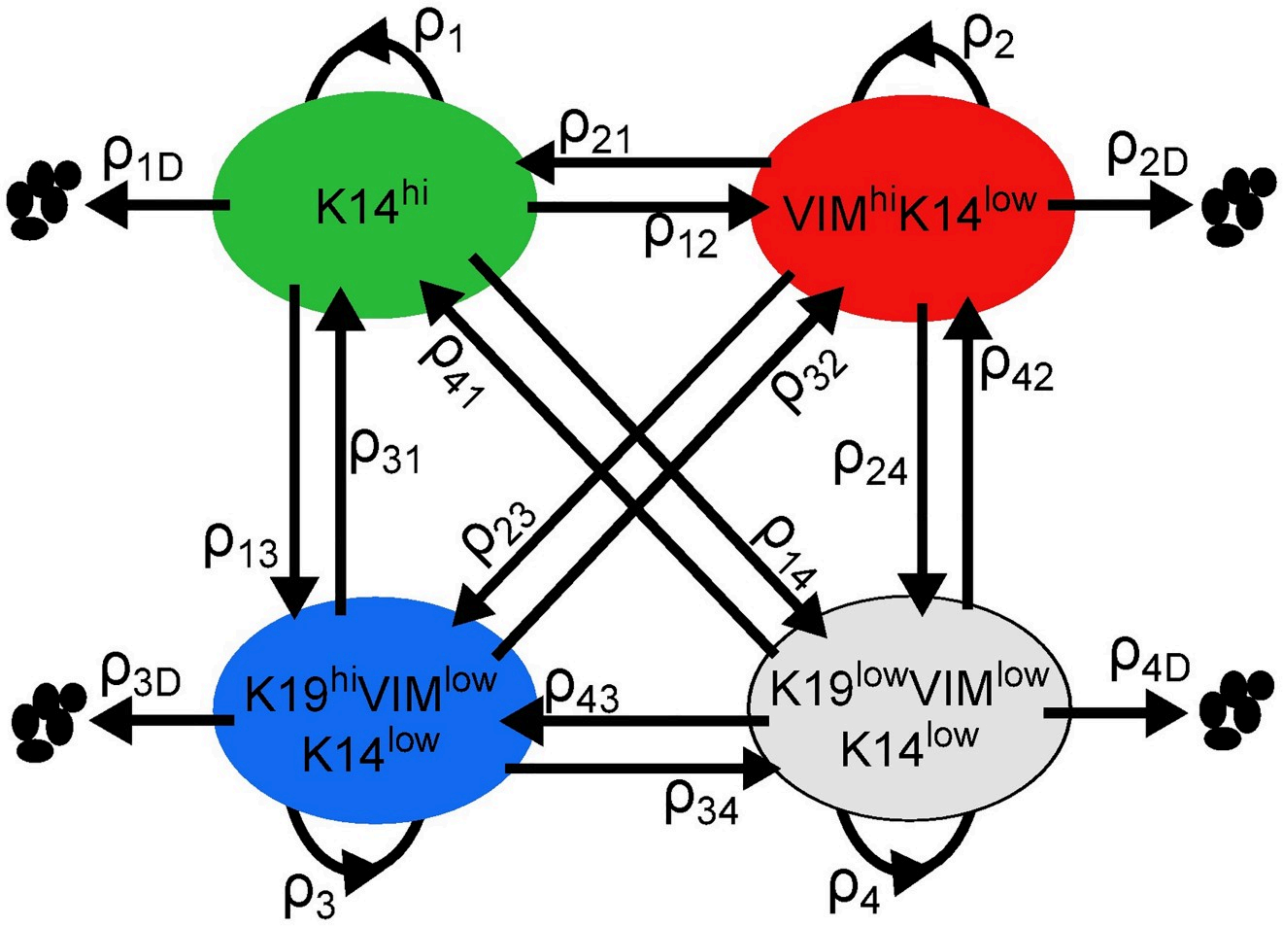
Drug-specific model. Live cells occupy four differentiation states and can transition, divide, or die. The dynamics parameters {*ρ*_*ij*_, *ρ*_*i*_, *ρ*_*i*D_}_*i*≠*j*_ are the average rates of these actions taken by live cells in each differentiation state following treatment.

We identified a dynamic model of the form depicted in Fig 1 to characterize the evolution of the four differentiation-state subpopulations in response to a given treatment condition (Trametinib, BEZ235, Trametinib+BEZ235, or DMSO). These models quantify how the number of live cells in each differentiation state and the number of dead or dying cells in total change over the time horizon (0h, 12h, …, 72h) following initial treatment. The key feature of each drug-specific model is the dynamics matrix, which contains the average rates of cell division, cell death, and transition between the four differentiation states. Specifically, these *dynamics parameters* are defined as follows: *ρ*_*i*_ is the *division gain* of differentiation state *i*; *ρ*_*i*D_ is the *death gain* of differentiation state *i*; *ρ*_*ij*_ is the *transition gain* from differentiation state *i* to differentiation state *j*. (A gain is a proportional value that quantifies the relationship between the magnitude of an input and the magnitude of an output and is a discrete-time analog of a rate).

We defined the four differentiation states according to binary expression levels of the basal marker Cytokeratin 14 (K14), the mesenchymal marker Vimentin (VIM), and the luminal marker Cytokeratin 19 (K19), as follows: 1) K14^hi^ (basal), 2) VIM^hi^K14^low^ (mesenchymal), 3) K19^hi^K14^low^VIM^low^ (luminal), and 4) K19^low^K14^low^VIM^low^ (non-basal/non-mesenchymal/non-luminal, or DSNS for brevity). For example, *ρ*_12_ is the transition gain from K14^hi^ to VIM^hi^K14^low^, and *ρ*_3_ is the division gain of K19^hi^K14^low^VIM^low^. Cells defined by dominant expression of luminal (K19), basal (K14), and mesenchymal (VIM) markers make up the majority of cells found in normal and neoplastic breast tissue, and luminal, basal, and mesenchymal tumor cell states predominate specific breast tumor subtypes [6] [25] [26]. Moreover, recent work from our group [18] and others [27] demonstrates that many triple-negative tumors contain heterogeneous cell populations characterized by the four states that we have defined. Functionally, mesenchymal-differentiated cells have been associated with enhanced stemness [10] and resistance to numerous therapeutics [15] [16] [17]. Likewise, luminal-differentiated and basal-differentiated breast cancer cells have particular drug sensitivities [31] [25] [27], and cells have been shown to transition between these states *in vitro* [9] [12] [32] and *in vivo* [32] [27]. These four differentiation states therefore represent major biologically significant cell states of breast tumors, and understanding their rates of growth, death, and transition during treatment is key to improving therapeutic strategies.

Our system identification problem is to estimate a representative ensemble of sets of dynamics parameters using the training data for each treatment condition. An ensemble of representative models can be useful for predicting trends when not all parameters are fully constrained by the available data, which is commonplace in systems biology [33] [34] [35] [36]. First, we identified a dynamics matrix and a data matrix using an alternating minimization (AM) algorithm [28] in which a convex optimization program [29] was solved at each iteration to reduce measurement error, process error, and estimation error; we specify the dynamics matrix and the data matrix returned by the algorithm at this stage as *AM-optimized*. By applying resampling residuals bootstrap [30] to the AM-optimized data matrix, we then generated multiple representative training data sets to identify an ensemble of dynamics matrices, or *model ensemble*. (We will later analyze the values of the dynamics parameters provided by the AM-optimized dynamics matrix and the 95% confidence intervals provided by the model ensemble for each treatment condition).

**Fig 2:**
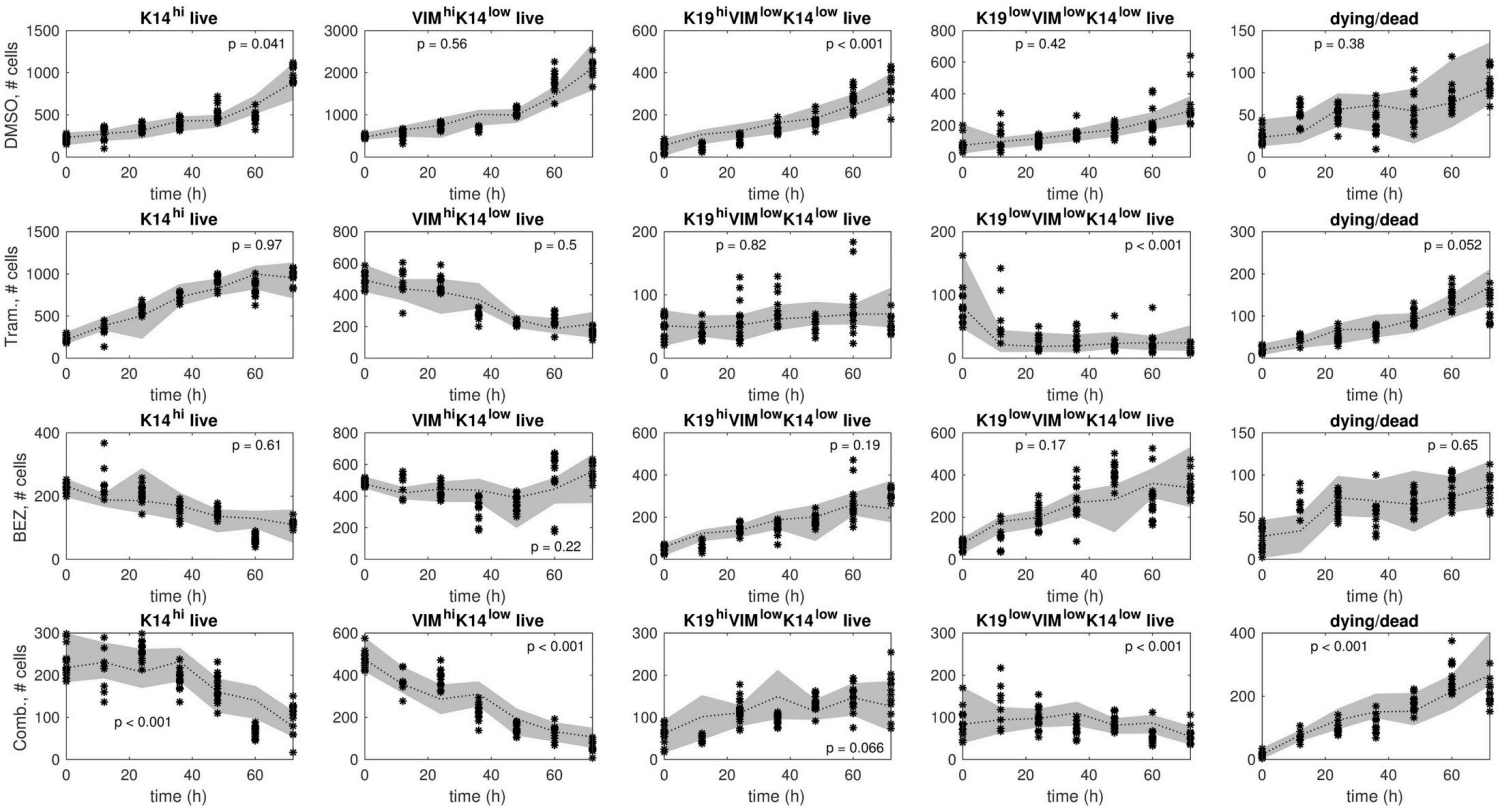
Ensemble model predictions in comparison to training data. The training samples (black stars) and predictions by the model ensemble (gray bands) are shown for each treatment condition: DMSO (row 1), Trametinib (row 2), BEZ235 (row 3), and Trametinib+BEZ235 (row 4). The model ensemble is a collection of models that were identified from the training data via resampling residuals bootstrap [30] for each treatment condition. In each plot, we show a 95% confidence interval (gray band) around the median (black dotted line) of the ensemble model predictions. *Higher* p-values indicate *better* consistency between predictions and training data over the time horizon (12h, 24h, …, 72h).

Predictions using the model ensemble in comparison to training data are shown in Fig 2 for each treatment. The model ensemble predicts the training data well, which is evident by qualitative and quantitative agreement. The predictions and the training data display comparable first-order trends (Fig 2). Further, few significant differences between predictions and training data were detected: most p-values in Fig 2 are larger than the 5% significance threshold, and these *larger* p-values indicate *lack of significant disagreement* between predictions and training data.

System identification for this paper requires time series data of the numbers of live cells in each differentiation state and the numbers of dead or dying cells summed over all states. However, the available time series data contain (a) numbers of live and dying cells in total occupying each differentiation state and (b) numbers of live and dying cells in total along with numbers of dying cells, with the caveat that the totals in these two subsets do not necessarily match (data (a) and data (b) were acquired from separate plates [18]). Specifically, differentiation-state marker expression of a cell and whether that cell was alive or dying could not be observed simultaneously since dying cells show false positivity for all markers. Thus, we undertook preliminary work to infer from the available data how death might be distributed across the differentiation states, which S1 Appendix presents in detail. To summarize, we distributed the observed death across the differentiation states in distinct ways to compute different sets of estimates of the numbers of live cells occupying each state. (The number of dead/dying cells over all states was assigned to the observed death fraction times the number of cells counted in all differentiation states). We trained and tested models on these different sets and found that model fitting errors were similar for different death distributions for each treatment condition (S1 Appendix). This finding may be attributed to the more prominent mechanism of differentiation-state transition in HCC1143 cells [18]. In view of this preliminary work, we distributed the observed death evenly across the differentiation states to compute the data samples (numbers of live cells in each differentiation state and numbers of dead/dying cells in all states) for the current paper.

**Fig 3:**
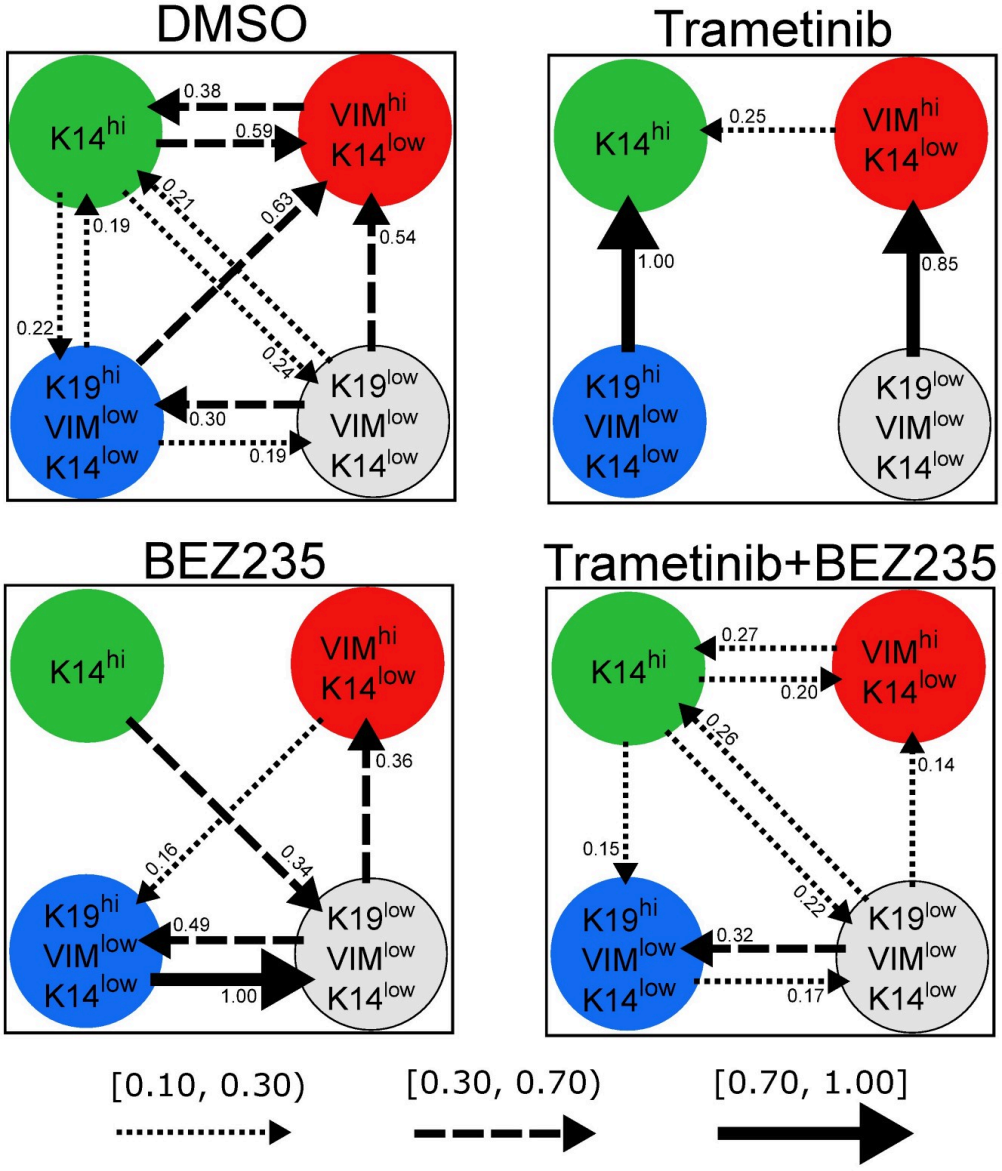
Drug-specific transition gains. For each treatment condition, values of the transition gains from the AM-optimized dynamics matrix are shown (units: $\frac{\#\text{cells}\;\text{at}\;(k+1)\;\text{multiples}\;\text{of}\;12\;\text{hours}}{\#\text{cells}\;\text{at}\;k\;\text{multiples}\;\text{of}\;12\;\text{hours}}$). Each transition gain from differentiation state *i* to differentiation state *j* of sufficient magnitude (*ρ_ij_* ≥ 0.10) is depicted as an arrow directed from *i* to *j*. Arrow style specifies gain magnitude. A dotted arrow means *ρ_ij_* ∈ [0.10, 0.30), a dashed arrow means *ρ_ij_* ∈ [0.30, 0.70), and a solid arrow means *ρ_ij_* ∈ [0.70, 1.00].

We used existing knowledge to impose constraints for system identification. For each treatment condition, we assumed that the four differentiation states have equal division gains (*ρ*_1_ = *ρ*_2_ = *ρ*_3_ = *ρ*_4_) because the HCC1143 cell line data generally showed similar percentages of EdU-positive cells for the distinct differentiation-state marker expression levels at any given time point under any given treatment. (EdU is incorporated into dividing cells as an indicator of proliferation. S1 Figure provides EdU^+^ data for each marker expression level under DMSO. Ref. [18] Figure 3b provides EdU^+^ data for each marker expression level under Trametinib or BEZ235. Ref. [18] Supplementary Figure 10c provides EdU^+^ data for each marker expression level under Trametinib+BEZ235). For each treatment condition, we also assumed that the four differentiation states have equal death gains (*ρ*_1D_ = *ρ*_2D_ = *ρ*_3D_ = *ρ*_4D_) in view of our preliminary work (see previous paragraph), in addition to the conclusion that drug-tolerant persister states induced by MEK or PI3K/mTOR inhibitors arise through differentiation-state transitions rather than state-specific death of pre-existing subpopulations [18]. This conclusion is supported by an empirical observation suggesting that cell death is independent of the differentiation-state changes induced by targeted therapy. Specifically, the combination of the pan-caspase inhibitor Z-VAD-FMK with Trametinib or BEZ235 significantly reduced the cell death incurred by these drugs, but negligible effects on the differentiation-state changes were observed [18]. The conclusion is further supported by simulations of basal/non-basal differentiation-state dynamic models [18]. The above assumptions cannot be relaxed by adding more parameters because the quantitative data necessary to estimate the additional parameters is not available.

### Modeling predicts drug-induced changes in differentiation-state transitions linked to therapeutic escape

**Fig 4:**
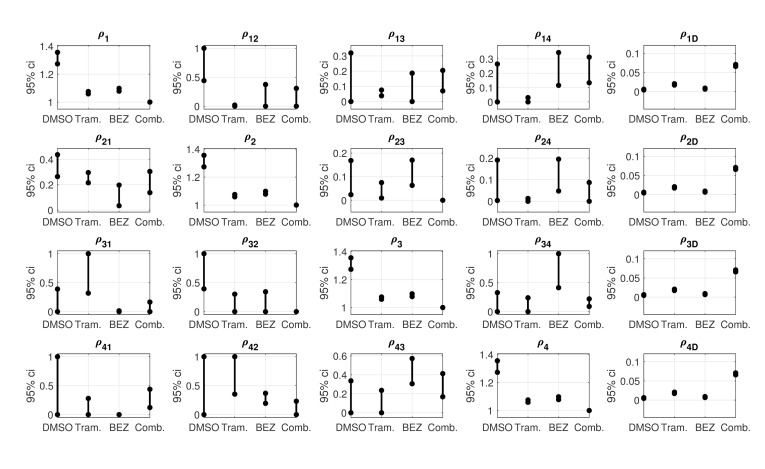
Uncertainty analysis of the dynamics parameters. For each treatment condition, 95% confidence intervals computed from the model ensemble are shown. These intervals indicate variations of the dynamics parameters due to measurement noise. Non-overlapping intervals of a given parameter specify a statistically significant difference. For example, a statistically significant reduction in K14^hi^-to-VIM^hi^K14^low^ transition was detected under Trametinib versus DMSO because the *ρ*_12_-interval for Trametinib is strictly below the *ρ*_12_-interval for DMSO. A p-value for each dynamics parameter is also provided in S2 Appendix.

While the relevance of basal/non-basal transitions to the emergence of drug-tolerant persister cell subpopulations has been reported [18], the nature of the transitions between the four differentiation states in triple-negative breast cancer (basal, mesenchymal, luminal, DSNS) is not well-understood. Here we predict the changes in differentiation-state transitions that underlie the six major differences in marker expressions induced by therapy in the HCC1143 cell line: 1) Trametinib-induced K14^hi^ enrichment, 2) BEZ235-induced K14^hi^ de-enrichment, 3) BEZ235-induced K19^low^VIM^low^K14^low^ enrichment, 4) Trametinib-induced K19^low^VIM^low^K14^low^ de-enrichment, 5) Trametinib+BEZ235-induced K19^hi^VIM^low^K14^low^ enrichment, and 6) Trametinib+BEZ235-induced VIM^hi^K14^^low^^ de-enrichment (see [18], Figures 3a and 4f). Specifically, we analyze the transition gains that were identified under therapy (Trametinib, BEZ235, or Trametinib+BEZ235) in comparison to DMSO, using the values from each drug-specific AM-optimized dynamics matrix (Fig 3) and the 95% confidence intervals from each drug-specific model ensemble (Fig 4). (*AM-optimized* and *model ensemble* were formerly specified in the “Drug-specific differentiation-state dynamic models” subsection).

#### Trametinib-induced K14^hi^ enrichment (vs. DMSO)

Our results indicate that reduced K14^hi^-to-VIM^hi^K14^low^ transition is fundamental to the K14^hi^ enrichment that follows Trametinib treatment. The K14^hi^-to-VIM^hi^K14^low^ transition gain *ρ*_12_ is significantly reduced under Trametinib versus DMSO because the *ρ*_12_-confidence interval for Trametinib is strictly below that for DMSO (Fig 4). No significant difference in the reverse direction, VIM^hi^K14^low^ to K14^hi^, was detected under Trametinib versus DMSO since the *ρ*_21_-confidence intervals for Trametinib and DMSO overlap (Fig 4).

Increased transition from K19^hi^VIM^low^K14^low^ to K14^hi^ may also underlie the K14^hi^ enrichment in Trametinib-treated cells. In particular, the associated transition gain is maximal for Trametinib, *ρ*_31_ = 1, and five times smaller for DMSO, *ρ*_31_ = 0.19 (Fig 3). No significant increase was detected because the *ρ*_31_-confidence intervals for Trametinib and DMSO overlap, but the amount of overlap is small compared to the length of either interval. The *ρ*_31_-confidence interval for Trametinib is [0.32, 1], and the *ρ*_31_-confidence interval for DMSO is [0, 0.39] (Fig 4).

**Fig 5:**
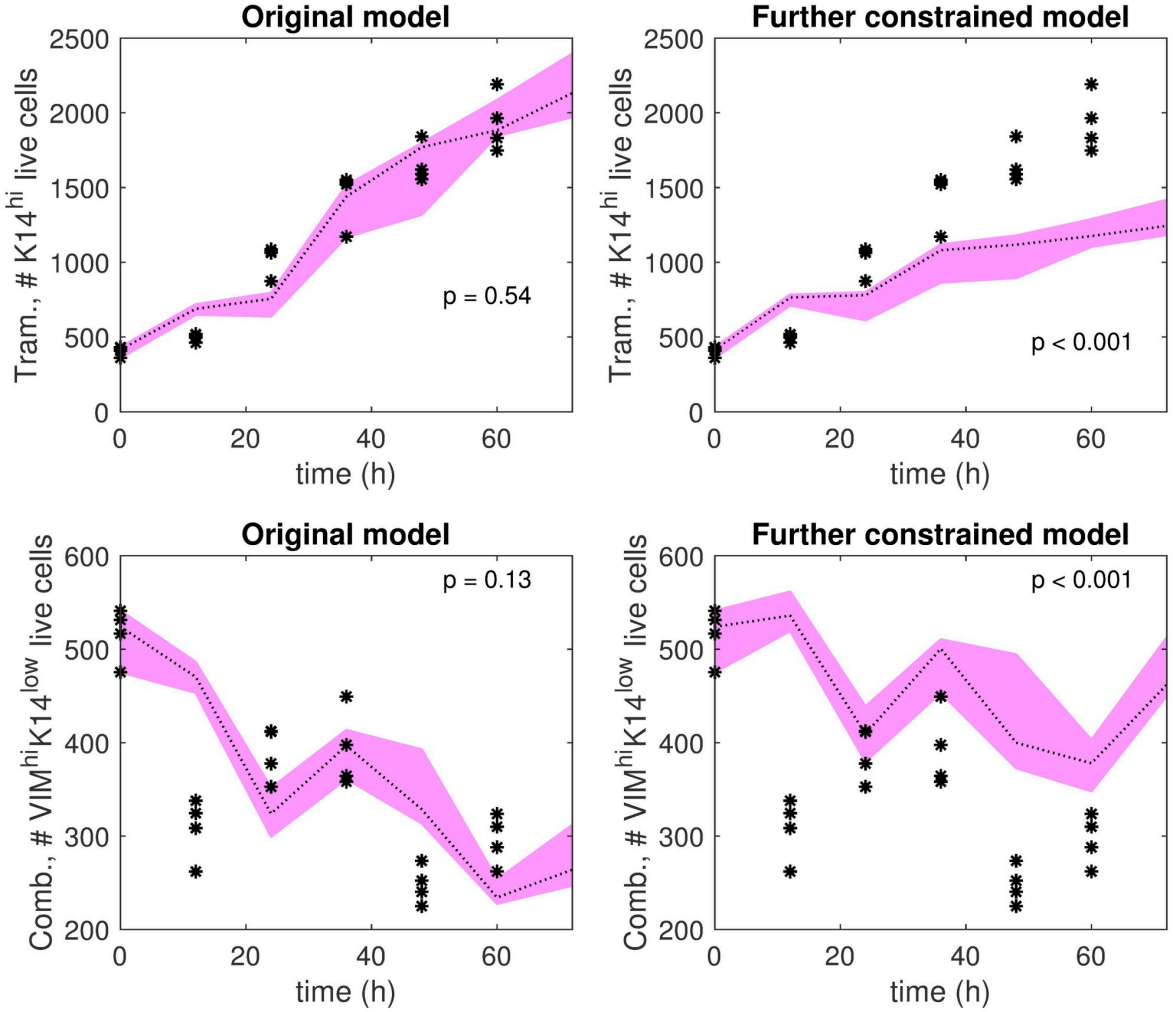
Further investigations of Trametinib-induced K14^hi^ enrichment hypothesis and Trametinib+BEZ235-induced VIM^hi^K14^low^ de-enrichment hypothesis. **Top left**: Trametinib K14^hi^ live cell predictions by the AM-optimized dynamics matrix (pink band) are shown in comparison to test data (black stars). **Top right**: Trametinib K14^hi^ live cell predictions by a dynamics matrix identified with additional constraints (pink band) in comparison to test data (black stars). The additional constraints are *ρ*_12_ ≥ 0.59, the value of *ρ*_12_ for DMSO, and *ρ*_31_ ≤ 0.19, the value of *ρ*_31_ for DMSO. (*ρ*_12_ is the K14^hi^-to-VIM^hi^K14^low^ transition gain, and *ρ*_31_ is the K19^hi^VIM^low^K14^low^-to-K14^hi^ transition gain). **Bottom left**: Trametinib+BEZ235 VIM^hi^K14^low^ live cell predictions by the AM-optimized dynamics matrix (pink band) are shown in comparison to test data (black stars). **Bottom right**: Trametinib+BEZ235 VIM^hi^K14^low^ live cell predictions by a dynamics matrix identified with additional constraints (pink band) are shown in comparison to test data (black stars). In this setting, the additional constraints are *ρ*_12_ ≥ 0.59, the value of *ρ*_12_ for DMSO, and *ρ*_32_ ≥ 0.63, the value of *ρ*_32_ for DMSO. (*ρ*_12_ is the K14^hi^-to-VIM^hi^K14^low^ transition gain, and *ρ*_32_ is the K19^hi^VIM^low^K14^low^-to-VIM^hi^K14^low^ transition gain). In each plot, the pink band extends between the maximum prediction and the minimum prediction out of four predictions in total at each time point (0h, 12h, …, 72h). The dotted line indicates the median of the predictions. Higher p-values indicate better consistency between predictions and test data over the time horizon (12h, 24h, …, 60h).

To further examine our predictions, we trained another dynamics matrix for Trametinib with two additional constraints: (i) *ρ*_12_ ≥ 0.59, which is the value of the DMSO K14^hi^-to-VIM^hi^K14^low^ transition gain, and (ii) *ρ*_31_ ≤ 0.19, which is the value of the DMSO K19^hi^VIM^low^K14^low^-to-K14^hi^ transition gain (Fig 3). The top row of Fig 5 shows the K14^hi^ live cell trajectories predicted by the further constrained dynamics matrix and those predicted by the (Trametinib) AM-optimized dynamics matrix in comparison to test data. The AM-optimized dynamics matrix provides trajectories that demonstrate qualitative and quantitative consistency with the test data, whereas the further constrained dynamics matrix fails in these regards. This simulation result supports our prediction that decreased K14^hi^-to-VIM^hi^K14^low^ transition or increased K19^hi^VIM^low^K14^low^-to-K14^hi^ transition underlie the K14^hi^ enrichment that follows Trametinib treatment in comparison to DMSO.

#### BEZ235-induced K14^hi^ de-enrichment (vs. DMSO)

Consider the collection of transition gains that involve the K14^hi^ differentiation state, {(*ρ*_12_, *ρ*_21_), (*ρ*_13_, *ρ*_31_), (*ρ*_14_, *ρ*_41_)}. Interestingly, for DMSO, the gains of any such pair have comparable non-zero magnitudes. For example, *ρ*_13_ = 0.22 and *ρ*_31_ = 0.19 for DMSO (Fig 3). But, the values of *ρ*_13_ and *ρ*_31_ are both zero for BEZ235 (S1 Table), suggesting negligible transition in either direction between K14^hi^ and K19^hi^VIM^low^K14^low^ in BEZ235-treated cells. For DMSO, the transition gains between K14^hi^ and VIM^hi^K14^low^ have values near one-half, *ρ*_12_ = 0.59 and *ρ*_21_ = 0.38, but for BEZ235 these gains have much smaller values, *ρ*_12_ = 0 and *ρ*_21_ = 0.05 (S1 Table). The BEZ235-induced reductions in these particular transitions are statistically significant because the *ρ*_12_-confidence interval and the *ρ*_21_-confidence interval for BEZ235 are strictly below those for DMSO (Fig 4). Further, for DMSO, the magnitudes of the transition gains between K14^hi^ and K19^low^VIM^low^K14^low^ are comparable and non-zero: *ρ*_14_ = 0.24 and *ρ*_41_ = 0.21 (Fig 3). For BEZ235, no transition is predicted from K19^low^VIM^low^K14^low^ to K14^hi^, *ρ*_41_ = 0, but some transition is predicted in the reverse direction, *ρ*_14_ = 0.34 (S1 Table, Fig 3).

Our modeling suggests that DMSO exhibits a *balancing effect* on the K14^hi^ differentiation state. Indeed, for DMSO, the proportion of cells transitioning from K14^hi^ to another differentiation state is predicted to be similar to the proportion of cells transitioning in the reverse direction over a 12h period. Our modeling predicts that BEZ235 treatment destabilizes this balance towards de-enrichment of the K14^hi^ state. BEZ235-treated cells are predicted to transition out of K14^hi^ to K19^low^VIM^low^K14^low^, and the rates of transition back into the K14^hi^ state are predicted to be near zero.

#### BEZ235-induced K19^low^VIM^low^K14^low^ enrichment (vs. DMSO)

BEZ235 treatment enriches the K19^low^VIM^low^K14^low^ state at the expense of the K14^hi^ state in the HCC1143 cell line [18]. The modeling predicts increased transition from K14^hi^ to K19^low^VIM^low^K14^low^ and reduced transition in the reverse due to BEZ235 therapy; *ρ*_14_ = 0.34 and *ρ*_41_ = 0 for BEZ235, but *ρ*_14_ = 0.24 and *ρ*_41_ = 0.21 for DMSO (S1 Table, Fig 3). Further, a statistically significant increase in transition from K19^hi^VIM^low^K14^low^ to K19^low^VIM^low^K14^low^ was detected under BEZ235 versus DMSO, enriching the latter state directly; the *ρ*_34_-confidence interval for BEZ235 is positioned above the *ρ*_34_-confidence interval for DMSO without overlap (Fig 4). Moreover, our results indicate decreased transition from K19^hi^VIM^low^K14^low^ to VIM^hi^K14^low^ in BEZ235-treated cells, which may permit more cells to transition from K19^hi^VIM^low^K14^low^ to K19^low^VIM^low^K14^low^ instead. The *ρ*_32_-confidence interval for BEZ235 is situated below the *ρ*_32_-confidence interval for DMSO without overlap, indicting a statistically significant result (Fig 4). To summarize, our modeling indicates that: (i) increased K14^hi^-to-K19^low^VIM^low^K14^low^ transition, (ii) decreased K19^low^VIM^low^K14^low^-to-K14^hi^ transition, (iii) increased K19^hi^VIM^low^K14^low^-to-K19^low^VIM^low^K14^low^ transition, or (iv) decreased K19^hi^VIM^low^K14^low^-to-VIM^hi^K14^low^ transition underlie the K19^low^VIM^low^K14^low^ enrichment in BEZ235-treated cells relative to DMSO.

#### Trametinib-induced K19^low^VIM^low^K14^low^ de-enrichment (vs. DMSO)

For DMSO, our modeling predicts similar non-zero rates of transitions between K19^low^VIM^low^K14^low^ and K19^hi^VIM^low^K14^low^ (*ρ*_34_ = 0.19, *ρ*_43_ = 0.30) and between K19^low^VIM^low^K14^low^ and K14^hi^ (*ρ*_14_ = 0.24, *ρ*_41_ = 0.21) (Fig 3). In contrast, for Trametinib, our results indicate minimal transitions between these two pairs of differentiation states; *ρ*_34_ = 0 and *ρ*_43_ = 0.03 as well as *ρ*_14_ = 0.03 and *ρ*_41_ = 0 (S1 Table). The K19^low^VIM^low^K14^low^-to-VIM^hi^K14^low^ transition gain for DMSO (*ρ*_42_ = 0.54) and the one for Trametinib (*ρ*_42_ = 0.85) are both large, though the latter is greater than the former (Fig 3). Together, these results suggest that increased transition from K19^low^VIM^low^K14^low^ to VIM^hi^K14^low^ or reduced transition into K19^low^VIM^low^K14^low^ may be fundamental to the K19^low^VIM^low^K14^low^ de-enrichment that follows Trametinib treatment relative to DMSO.

To provide more insight, we analyzed our results for Trametinib in comparison to those for BEZ235, since K19^low^VIM^low^K14^low^ is de-enriched in Trametinib but enriched in BEZ235 relative to DMSO [18]. In particular, the *ρ*_42_-confidence interval for Trametinib is [0.35, 1], and the *ρ*_42_-confidence interval for BEZ235 is [0.19, 0.37] (Fig 4). The intervals overlap, though only slightly, which supports our prediction that increased K19^low^VIM^low^K14^low^-to-VIM^hi^K14^low^ transition may underlie the Trametinib-induced K19^low^VIM^low^K14^low^ de-enrichment. Further, statistically significant reductions in transitions were detected between K19^low^VIM^low^K14^low^ and K19^hi^VIM^low^K14^low^ under Trametinib versus BEZ235; the *ρ*_43_-confidence interval and the *ρ*_34_-confidence interval for Trametinib are both strictly beneath those for BEZ235 (Fig 4). Because both *ρ*_43_ and *ρ*_34_ decrease significantly under Trametinib versus BEZ235, any changes in the transition rates between K19^low^VIM^low^K14^low^ and K19^hi^VIM^low^K14^low^ are not likely to contribute to the K19^low^VIM^low^K14^low^ de-enrichment. However, significant decreases in *ρ*_14_ and *ρ*_24_ were detected under Trametinib versus BEZ235 (Fig 4).

Taken together, our results suggest that increased K19^low^VIM^low^K14^low^-to-VIM^hi^K14^low^ transition, or decreased transition into K19^low^VIM^low^K14^low^, feasibly from K14^hi^ or VIM^hi^K14^low^, underlie the Trametinib-induced K19^low^VIM^low^K14^low^ de-enrichment relative to DMSO.

#### Trametinib+BEZ235-induced K19^hi^VIM^low^K14^low^ enrichment and VIM^hi^K14^low^ de-enrichment (vs. DMSO)

Given the divergent differentiation-state enrichments following Trametinib and BEZ235 (K14^hi^ and K19^low^VIM^low^K14^low^, respectively) [18], it is not surprising that a combination of these drugs causes the residual cells to aggregate into a distinct state. Trametinib+BEZ235 treatment enriches the K19^hi^VIM^low^K14^low^ differentiation state at the expense of the VIM^hi^K14^low^ state in the HCC1143 cell line [18]. Reduced transition from K19^hi^VIM^low^K14^low^ to VIM^hi^K14^low^ is predicted to play a significant role in these changes. The *ρ*_32_-confidence interval for Trametinib+BEZ235 hovers near zero, while the *ρ*_32_-confidence interval for DMSO extends from 0.39 to 1 (Fig 4). Our modeling also indicates that reduced transition from K14^hi^ to VIM^hi^K14^low^ contributes to the VIM^hi^K14^low^ de-enrichment following Trametinib+BEZ235 treatment. The *ρ*_12_-confidence interval for Trametinib+BEZ235 is positioned below the *ρ*_12_-confidence interval for DMSO without overlap, indicating a statistically significant result (Fig 4). Thus, we predict that decreased K14^hi^-to-VIM^hi^K14^low^ transition or decreased K19^hi^VIM^low^K14^low^-to-VIM^hi^K14^low^ transition underlie the VIM^hi^K14^low^ de-enrichment that occurs under Trametinib+BEZ235 relative to DMSO.

To further test this prediction, we trained another dynamics matrix for Trametinib+BEZ235 with additional constraints: (i) *ρ*_12_ ≥ 0.59, which is the value of the DMSO K14^hi^-to-VIM^hi^K14^low^ transition gain, and (ii) *ρ*_32_ ≥ 0.63, which is the value of the DMSO K19^hi^VIM^low^K14^low^-to-VIM^hi^K14^low^ transition gain (Fig 3). The bottom row of Fig 5 shows the VIM^hi^K14^low^ live cell trajectories predicted by the further constrained dynamics matrix and those predicted by the (Trametinib+BEZ235) AM-optimized dynamics matrix in comparison to test data. The trajectories predicted by the further constrained matrix do not demonstrate quantitative or qualitative consistency with the test data, while the trajectories predicted by the AM-optimized dynamics matrix are consistent with the test data, evident by comparable trends and a sufficiently large p-value (p = 0.13 > 0.05). This simulation result supports our prediction that decreased K14^hi^-to-VIM^hi^K14^low^ transition or decreased K19^hi^VIM^low^K14^low^-to-VIM^hi^K14^low^ transition are fundamental to Trametinib+BEZ235-induced VIM^hi^K14^low^ de-enrichment.

### Empirical validation of division gains

The value of the division gain from each drug-specific AM-optimized dynamics matrix and the associated 95% confidence interval from each drug-specific model ensemble are shown in Table 1. The values indicate that cell division occurs most often under DMSO followed by BEZ235, Trametinib, and Trametinib+BEZ235 in decreasing order (Table 1). This ordering is consistent with empirical observations of the percentages of EdU-positive cells following these treatments (see [18], Figure 4c). The division gains of the cytotoxic therapies are significantly reduced compared to the DMSO division gain, indicated by non-overlapping *ρ*_*i*_-confidence intervals (Table 1). Consistent with these computational findings, smaller percentages of EdU-positive cells were detected 24h after treatment with Trametinib, BEZ235, and Trametinib+BEZ235 compared to DMSO, and these trends persisted over time [18]. In parallel, Gene Set Enrichment Analysis [37] revealed de-enrichment of proliferation gene sets in cells treated with Trametinib+BEZ235 relative to DMSO (see [18], Figure 4g). Further, the Trametinib+BEZ235 division gain is significantly reduced compared to the Trametinib division gain and the BEZ235 division gain (Table 1). Similarly, the percentages of EdU-positive cells are significantly reduced from 48h to 72h following Trametinib+BEZ235 treatment versus BEZ235 treatment, and these percentages are significantly reduced from 12h to 48h under Trametinib+BEZ235 versus Trametinib [18]. The above computational-empirical consistencies provide empirical validation for the representation of cell division in our models.

**Table 1:** Drug-specific division gains and death gains.

Drug	Division gain *ρ*_*i*_	Death gain *ρ*_*i*D_
DMSO	1.34 [1.27, 1.36]	0.0057 [0.0045, 0.0063]
Trametinib	1.07 [1.06, 1.08]	0.019 [0.017, 0.021]
BEZ235	1.09 [1.08, 1.10]	0.0083 [0.0067, 0.0091]
Trametinib+BEZ235	1.00 [1.00, 1.00]	0.068 [0.066, 0.071]

The value of the division gains *ρ*_*i*_ and the value of the death gains *ρ*_*i*D_ from each drug-specific AM-optimized dynamics matrix are provided (units: $\frac{\#\text{cells}\;\text{at}\;(k+1)\;\text{multiples}\;\text{of}\;12\;\text{hours}}{\#\text{cells}\;\text{at}\;k\;\text{multiples}\;\text{of}\;12\;\text{hours}}$), where *i* ∈ {1,2,3,4} is a differentiation-state index, *ρ*_1_ = *ρ*_2_ = *ρ*_3_ = *ρ*_4_, and *ρ*_1D_ = *ρ*_2D_ = *ρ*_3D_ = *ρ*_4D_. 95% confidence intervals from each drug-specific model ensemble are also shown. Higher values indicate more frequent division or death on average over time compared to lower values.

### Empirical validation of death gains

**Fig 6:**
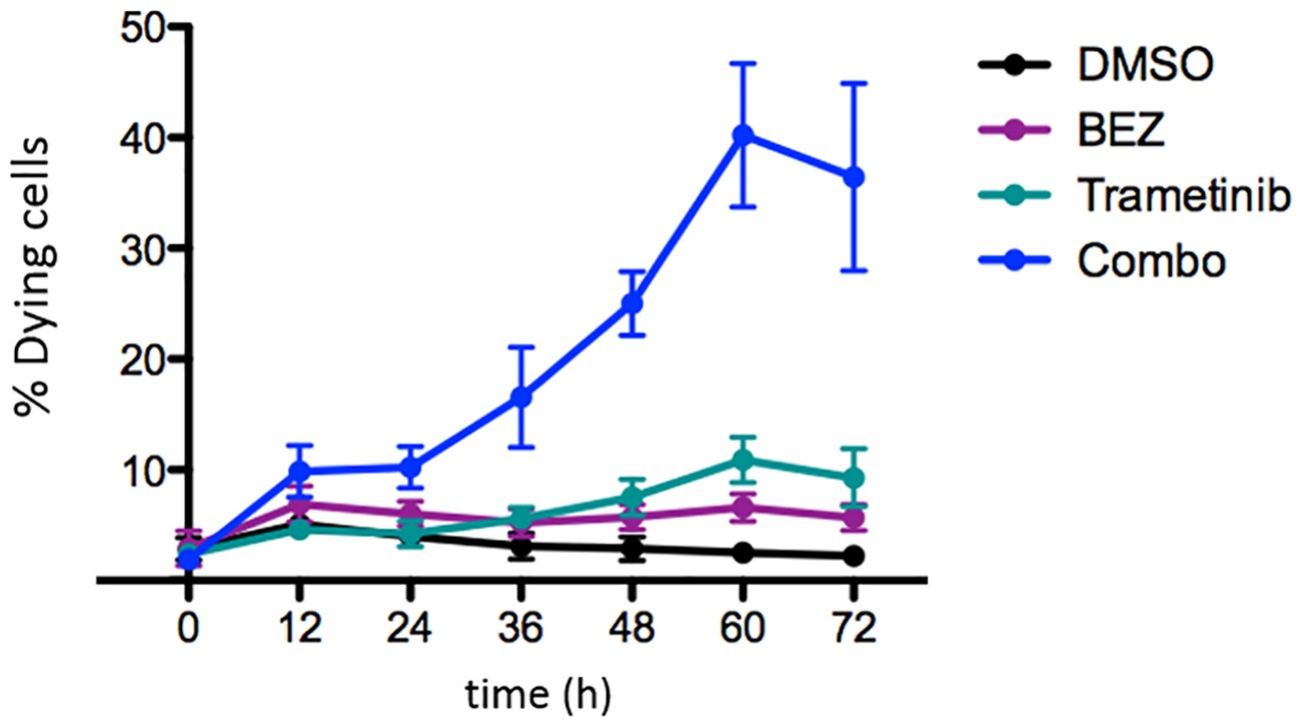
Death time series data for model training. The YO-PRO-1 dye was used to quantify the proportion of dying cells every 12h in response to drug treatment [18]. The cells were treated with DMSO (baseline), 1*μ*M Trametinib, 1*μ*M BEZ235, or the combination of 1*μ*M Trametinib + 1*μ*M BEZ235.

Our modeling indicates that Trametinib induces more death on average compared to BEZ235 (Table 1). Our models were trained using data consistent with this conclusion shown in Fig 6. However, additional data that was not used for training shows that BEZ235 generally induces more cell death than Trametinib (see [18], Figure 4b).

Our modeling specifies that HCC1143 cells undergo apoptosis most often under Trametinib+BEZ235 treatment, according to the death gain values in Table 1. Further, the Trametinib+BEZ235 death gain is statistically significantly higher compared to the Trametinib death gain and the BEZ235 death gain; see the *ρ*_*i*D_-confidence intervals in Table 1. Similarly, the percentages of dying cells detected via YO-PRO-1 staining under Trametinib+BEZ235 are significantly higher relative to those under Trametinib or BEZ235 from 36h to 72h in the additional data (see [18], Figure 4b). This computational-empirical consistency provides experimental validation for the representation of cell death in our models, namely with respect to the superior cell-kill ability of the Trametinib+BEZ235 condition.

### Model testing on independent data

The 14 dynamics parameters of each drug-specific model were trained on 90 to 99 samples, depending on the drug, of HCC1143 cell line data (S1 Appendix), and the variations of these parameters were estimated by bootstrapping [30] the training data, providing statistically significant findings that we analyzed in prior sections (Fig 4). We further evaluated the models on test data (24 samples per treatment condition) that was collected roughly one year before the training data.

**Fig 7:**
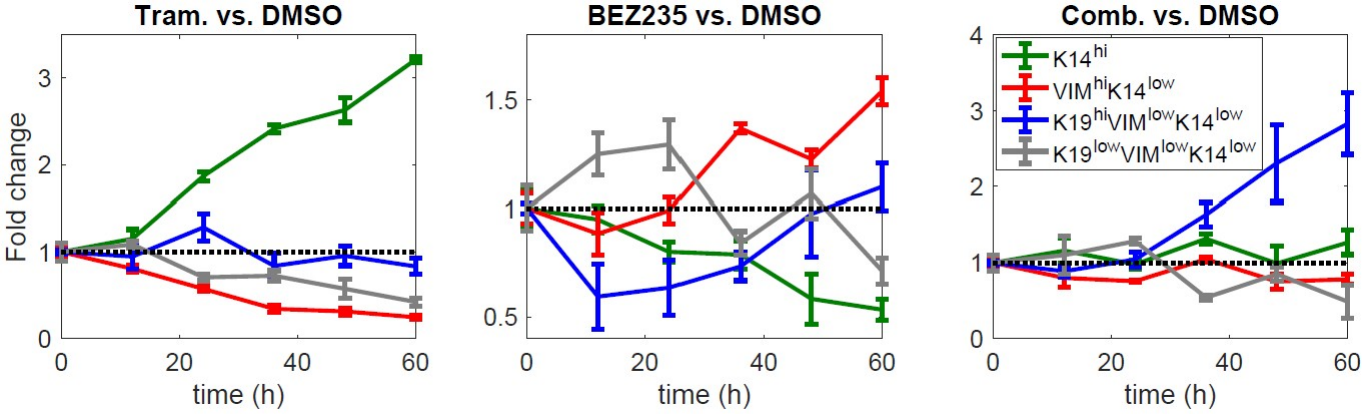
Differentiation-state time series portion of the test data. The sample mean and the sample standard deviation of fold change for each differentiation state are shown at each time point, computed from 4 samples. Fold change is $\frac{\text{fraction}\;\text{differentiation}\;\text{state}\;i,\;\text{well}\;w,\;\text{time}\;k,\;\text{therapy}}{\text{average}\;\text{fraction}\;\text{differentiation}\;\text{state}\;i,\;\text{time}\;k,\;\text{DMSO}}$, where fraction differentiation state *i* is the number of cells counted in that state divided by the population total.

While the experiment that provided the test data and the experiment that provided the training data were intended to be identical, a brief examination of the trends in these data reveals clear qualitative differences for the differentiation states defined by the K19 or VIM markers. We show the differentiation-state time series portion of the test data in Fig 7, and please see Risom et al., Figures 3a and 4f, for the differentiation-state time series portion of the training data [18]. For example, the training data indicates that the VIM^hi^K14^low^ BEZ235-versus-DMSO fold change stays below 1 after 24h [18]. However, the test data indicates that the VIM^hi^K14^low^ BEZ235-versus-DMSO fold change stays above 1 after 24h (Fig 7). To specify another example, the K19^low^K14^low^VIM^low^ Trametinib+BEZ235-versus-DMSO fold change is generally above 1 in the training data [18], whereas this is not true in the test data (Fig 7).

**Fig 8:**
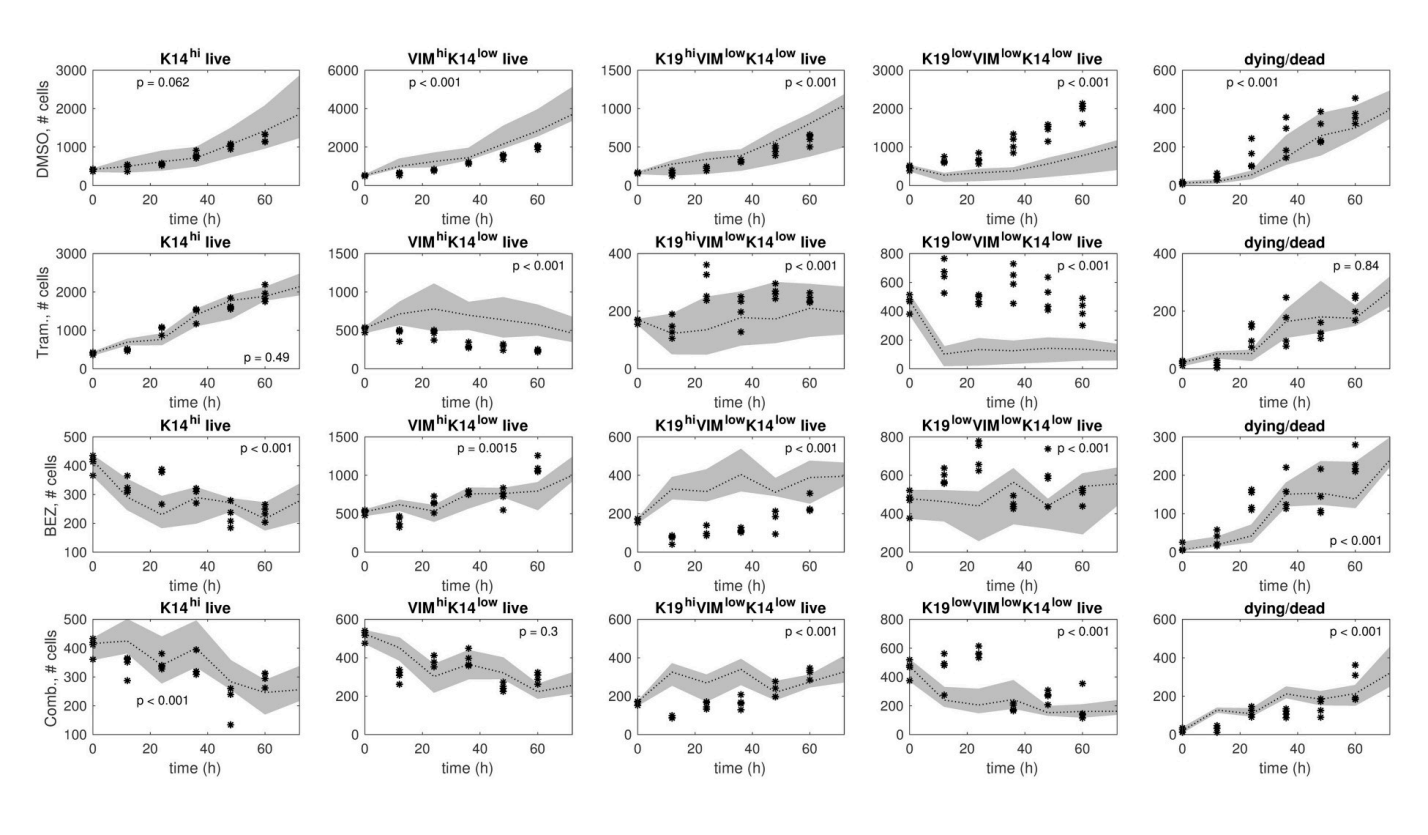
Ensemble model predictions in comparison to test data. The test samples (black stars) and ensemble model predictions (gray bands) are shown for each treatment condition: DMSO (row 1), Trametinib (row 2), BEZ235 (row 3), and Trametinib+BEZ235 (row 4). The model ensemble is a collection of models that were identified from the training data via resampling residuals bootstrap [30] for each treatment condition. In each plot, we show a 95% confidence interval (gray band) around the median (black dotted line) of the ensemble model predictions. *Higher* p-values indicate *better* consistency between predictions and test data over the time horizon (12h, 24h, …, 60h).

Predictions using the model ensemble in comparison to the test data are shown in Fig 8 for each treatment condition. This is a stringent assessment of the models since there are qualitative differences between the training data and the test data. Nonetheless, the ensemble model predictions and the test data demonstrate consistency in the number of K14^hi^ live cells under DMSO, the number of K14^hi^ live cells under Trametinib, and the number of VIM^hi^K14^low^ live cells under Trametinib+BEZ235, evident by comparable trends and lack of significant differences (Fig 8). There is also qualitative agreement between the predictions and the test data in the number of dead/dying cells for each treatment condition (Fig 8). In certain cases, the predictions and the test data both increase overall, although their respective rates of change differ; e.g., see VIM^hi^K14^low^ and K19^low^K14^low^VIM^low^ for DMSO (Fig 8). The most severe discrepancies involve the differentiation states defined by VIM or K19 (Fig 8), which can be explained partly by existing biological knowledge.

Vimentin and Cytokeratin 19 display a continuum of low expression to high expression in HCC1143 cells, which makes the low and high cutoffs more variable across replicate experiments and introduces noise into the subpopulation fractions (S2 Figure). Cytokeratin 14, however, is strongly expressed by a subset of cells and is weakly expressed, or lacks expression, in the other subset of cells (S2 Figure). This biphasic expression pattern forms distinct high and low subpopulations, so the fraction of cells in each subpopulation is more similar across replicate experiments.

**Fig 9:**
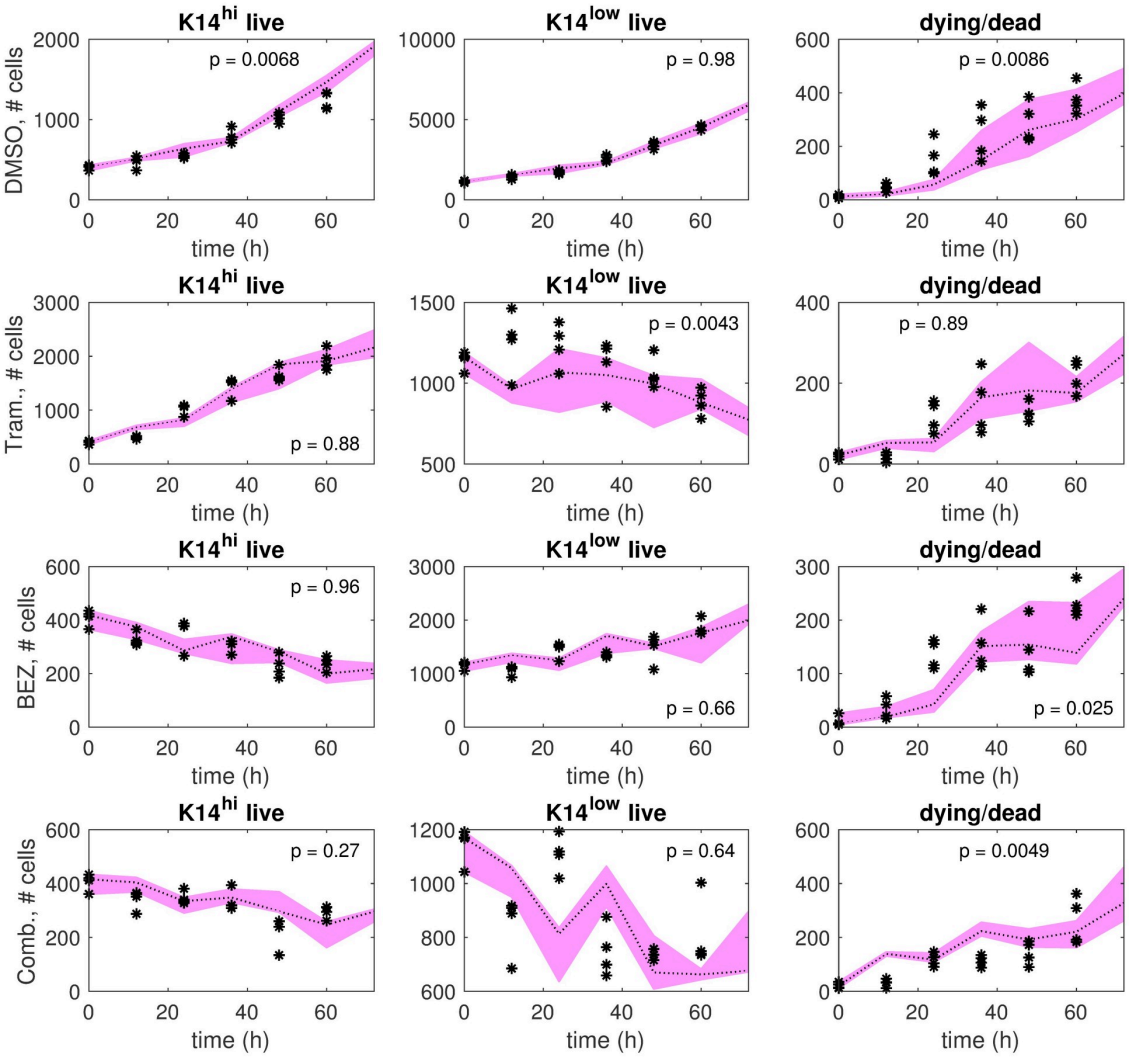
Single model predictions in comparison to test data, where the differentiation states are defined by K14 only. The test samples (black stars) and single model predictions (pink bands) are shown for each treatment condition: DMSO (row 1), Trametinib (row 2), BEZ235 (row 3), and Trametinib+BEZ235 (row 4). The single model was identified on the training data using K14^hi^ and K14^low^ as the differentiation states for each treatment condition. The pink band extends between the maximum prediction and the minimum prediction out of four predictions in total at each time point (0h, 12h, …, 72h). The dotted line indicates the median of the predictions. *Higher* p-values indicate *better* consistency between predictions and test data over the time horizon (12h, 24h, …, 60h).

Driven by these findings, for each treatment condition we identified a lower-dimensional dynamics matrix on the training data using K14^hi^ and K14^low^ as the differentiation-state definitions, and then evaluated how well this matrix could predict the test data. As shown in Fig 9, the predictions and the test data in this setting demonstrate qualitative consistency (comparable trends) and quantitative consistency (sufficiently large p-values, p > 0.05) for most cell types (K14^hi^ live, K14^low^ live, dead/dying) and treatment conditions.

## Discussion

In this study, we developed novel quantitative dynamic models to demonstrate how different treatments can distinctly affect the rates of differentiation-state transitions in the context of the four key states in triple-negative breast cancer. Using existing time series data of HCC1143-derived cell populations, we applied optimization algorithms to estimate dynamics parameters and their variations due to measurement noise (Figs 3 and 4, Table 1). We used these variations to detect statistically significant drug-induced effects on the rates of differentiation-state transition, cell division, and cell death. We validated several model predictions on cell division and cell death empirically. Our models predict how changes in transition rates may underlie specific differentiation-state aggregations of drug-tolerant cells reported by Risom et al. [18]. Simulations with respect to test data further substantiate certain predictions on drug-induced changes in differentiation-state transition rates (Fig 5).

Our model predictions indicate that small-molecule targeted therapy strongly affects differentiation-state transition rates relative to DMSO in the HCC1143 cell line (Fig 3). Robust but reciprocal transitions continually occurring under DMSO provides an environment where therapy-induced changes in the balance of transitions can provoke differentiation-state aggregations. Indeed, differentiation-state transitions are predicted to occur in the DMSO condition, and many pairwise transition rates are similar (Fig 3; e.g., *ρ*_14_ = 0.24 and *ρ*_41_ = 0.21, where state 1 is K14^hi^ and state 4 is K19^low^VIM^low^K14^low^). Both Trametinib and BEZ235 are predicted to reduce the rates of particular state-to-state transitions and increase the rates of others, leading to the distinct differentiation-state aggregations of drug-tolerant cells reported in [18]. Specifically, we found that reduced K14^hi^-to-VIM^hi^K14^low^ transition or increased K19^hi^VIM^low^K14^low^-to-K14^hi^ transition are key to the K14^hi^ enrichment that was observed in response to Trametinib (Fig 3 shows predicted transitions under Trametinib and DMSO; see [18], Figure 3a, for Trametinib vs. DMSO data). Secondly, increased K14^hi^-to-K19^low^VIM^low^K14^low^ transition, decreased K19^low^VIM^low^K14^low^-to-K14^hi^ transition, increased K19^hi^VIM^low^K14^low^-to-K19^low^VIM^low^K14^low^ transition, or decreased K19^hi^VIM^low^K14^low^-to-VIM^hi^K14^low^ transition are predicted to underlie the K19^low^VIM^low^K14^low^ enrichment observed after BEZ235 treatment (Fig 3 shows predicted transitions under BEZ235 and DMSO; see [18], Figure 3a, for BEZ235 vs. DMSO data). Also, reduced K19^hi^VIM^low^K14^low^-to-VIM^hi^K14^low^ transition is predicted to be critical to the K19^hi^VIM^low^K14^low^ enrichment following Trametinib+BEZ235 treatment (Fig 3 shows predicted transitions under Trametinib+BEZ235 and DMSO; see [18], Figure 4f, for Trametinib+BEZ235 vs. DMSO data).

We evaluated our models using a test data set that was collected separately from the training data set. The differentiation-state time series portion of the test data is presented in Fig 7, and the differentiation-state time series portion of the training data is presented in [18], Figures 3a and 4f. (These data are provided in spreadsheets in S1 Code/Training Data/Test Data) The K14^hi^ trends are similar in the training data and the test data, but this is not necessarily true for the trends of the other states. Thus, the model predictions and the test data are generally more consistent for the K14^hi^ live cells and less consistent for the live cells in the states defined by K19 or VIM (Fig 8). The latter outcome is likely due to the continuum of expression levels of K19 and VIM (S2 Figure), making “high” state calls more noisy and suggesting that identification of more robust, differentially expressed lineage markers could improve consistency between model predictions and test data in the future. Lower-dimensional models of two differentiation states, K14^hi^ and K14^low^, yielded predictions that demonstrate improved consistency with the test data (Fig 9); this result is not surprising due to the biphasic expression pattern of K14 (S2 Figure) and the reduction in the number of parameters that required identification. Our higher-dimensional models are also valid in a statistical sense because between 90 to 99 samples (depending on the particular drug) were used to train the 14 parameters of each model (S1 Appendix). Further, variations of these parameters were estimated via resampling residuals “wild” bootstrap [30], and several statistically significant differences were detected (Fig 4). It is important that we identified a moderate number of parameters to help mitigate overfitting the data available for each treatment condition [38].

It should be noted that evaluating the generalizability of the models on test data that was collected separately from the training data is a stringent approach. (If enough data from a single experiment is available, it is common to choose the training set and the test set from this one experiment to avoid inter-experimental variability). Nonetheless, our testing results substantiate our prediction that decreased K14^hi^-to-VIM^hi^K14^low^ transition or increased K19^hi^VIM^low^K14^low^-to-K14^hi^ transition underlie Trametinib-induced K14^hi^ enrichment in HCC1143 cells. When both changes were inhibited computationally, the model predictions and the test data are inconsistent (Fig 5, top right); otherwise, the predictions and the test data are consistent qualitatively and quantitatively (Fig 5, top left). Our testing results also affirm that decreased K14^hi^-to-VIM^hi^K14^low^ transition or decreased K19^hi^VIM^low^K14^low^-to-VIM^hi^K14^low^ transition lead to VIM^hi^K14^low^ de-enrichment in Trametinib+BEZ235-treated cells. Indeed, the model predictions and the test data are inconsistent when both changes were inhibited in the model (Fig 5, bottom right), but the predictions and the test data demonstrate improved qualitative and quantitative consistency otherwise (Fig 5, bottom left).

Our models of the four differentiation states are powerful tools to infer the transition behaviors that may underlie differentiation-state aggregations of drug-tolerant cells induced by therapy. MEK and PI3K/mTOR inhibitors have been found to aggregate HCC1143 cells into distinct basal-differentiated and luminal-differentiated drug-tolerant persister states, respectively, evident by changed levels of K14, VIM, or K19 expression [18]. Immunofluorescent imaging and image cytometry have shown that treatment-naive TNBC tumors have high phenotypic heterogeneity, harboring subpopulations of cells that express the basal marker K14, the mesenchymal marker VIM, the luminal marker K19, or a combination of these intermediate filament markers [18]. These differentiation states have been shown to possess distinct sensitivity to therapeutics [31] [25] [16], making it critical to identify which states are aggregated post-treatment, and from which states these transitions occurred, in order to design improved therapeutic regimens. Quantitative models of the four states defined by K14, VIM, or K19 are necessary to better understand the differentiation-state heterogeneity of triple-negative breast cancer and more specifically, to predict the dynamics of differentiation-state transitions.

As well as predicting how targeted therapy affects transitions, another crucial prediction that we could not determine empirically—but is provided by our modeling—is that differentiation-state transitions occur continually under DMSO. This finding may be specific to the TNBC plasticity phenotype, as we found previously that other breast cancer subtypes (e.g., luminal breast cancers) do not display differentiation-state heterogeneity to the same extent [18]. In addition, if the baseline ability to transition between states is critical for the ability of these cells to survive therapeutic treatment, this could explain why the TNBC basal-like subtype is particularly sensitive to combinations of such state-aggregating drugs with the BET inhibitor JQ1, which prevents efficient chromatin rewiring [18].

Empirical validation of our predictions regarding differentiation-state transitions poses particular challenges. Current antibody-based techniques for assessing intracellular protein expression in cells grown in 2D require the permeabilization of the cell to permit antibody access to its antigens. To maintain structural integrity of the cell during this process, cell fixation is required. So, our phenotypic assessment of cells based on intermediate filament expression can be performed only in fixed cells. But, if cell-surface markers were found to correlate well with the four differentiation states in our study, then existing methods could be used to validate our hypotheses. A given state could be isolated via Fluorescence-Activated Cell Sorting [12], and then the homogeneous cell population could be treated and observed for changes in cell-surface marker expression.

The accuracy and the predictive power of differentiation-state dynamic models will improve as experimental methods improve. Since dying cells show false positivity for all markers, our instruments could not simultaneously detect the differentiation-state marker expression of a single cell and whether that cell was alive or dying. To manage this limitation, we distributed the observed death fractions evenly across the observed numbers of cells occupying each differentiation state to estimate the data samples required for modeling and subsequent analyses (S1 Appendix). Moreover, our instruments can only detect cells with intact nuclei, so dying cells can fade from view. This is one reason why the number of dead or dying cells in the data may decrease (e.g., see Fig 9). While empirical observations indicate time-varying rates of cell division and death, our models are restricted to encoding these rates on average (see [18], Figure 4c, for cell division data; Fig 6 shows death data; Table 1 provides division and death gains). There will be potential to relax the time-invariance assumption when more time series data is available to help mitigate overfitting [38].

Although more experiments are required to identify optimal strategies for targeted therapy in the HCC1143 cell line, administering therapies in moderate doses one-by-one, where the next drug and the waiting time before its application are chosen according to model predictions and recent measurements of the cells being treated, may effectively manage cancer growth, drug toxicity, and therapeutic resistance. In particular, it may be important to apply the next drug at the time of maximal signaling pathway activity induced by the previous drug [9] and take into account uncertainty due to unmodeled drug-drug interactions [39].

This paper predicts that treating HCC1143 cells with a MEK inhibitor, a PI3K/mTOR inhibitor, or a combination of these inhibitors alters specific rates of transitions between basal, mesenchymal, luminal, and DSNS states relative to DMSO. These predictions provide new biological insights into how changes in transition rates may underlie certain differentiation-state aggregations of drug-tolerant persister cells recently reported by [18]. In particular, our findings support differentiation-state transition as the major mechanism underlying resistance to MEK and PI3K/mTOR inhibitors. Our modeling work demonstrates the feasibility of this mechanism by predicting—with statistical rigor—the directionality of state transition in the absence of, and in the presence of, therapeutic pressure. Improved understanding of the directionality of state transition may inform the design of mechanistic studies that promote the development of superior treatment strategies for heterogeneous plastic cancers.

## Methods

### HCC1143 cell line experiments

Numbers of cells in each differentiation state, numbers of live and dying cells in total, and numbers of dying cells were observed from 15 replicate wells of the HCC1143 triple-negative breast cancer cell line every 12 hours over 7 time points following initial drug treatment [18]. The drugs were the MEK inhibitor Trametinib (1*μ*M), the PI3K/mTOR inhibitor BEZ235 (1*μ*M), the combination of 1*μ*M Trametinib + 1*μ*M BEZ235, and DMSO (baseline). Cellular phenotype was assessed by immunofluorescent imaging, using the combined expression of the basal marker Cytokeratin 14, the mesenchymal marker Vimentin, and the luminal marker Cytokeratin 19 to identify cellular phenotype [18]. The YO-PRO-1 cell death dye along with phase imaging were used to measure the numbers of live and dying cells in total and the numbers of dying cells [18]. Between 90 to 99 samples (depending on the particular drug) were used for training for each treatment condition (S1 Appendix). Although 105 samples were collected for each drug (15 wells × 7 time points per well = 105), several samples had to be discarded because of instrument errors. The primary error was loss of the imaging focal plane during plate scanning. Out-of-focus images failed automated single cell segmentation, were flagged, and were removed from the data set.

An independent data set was used for model testing. It was collected a year prior to the training data set and included 6 time points of measurements with 4 replicate wells imaged every 12 hours following initial drug treatment.

### System model

We modeled the evolution of differentiation-state heterogeneity in response to drug treatment as a switched linear time-invariant positive dynamical system [39] [40] [41] [42],

$$\begin{array}{c} \begin{array}{cc} x\left(k+1\right)=A_{\delta_{k}}\cdot x\left(k\right);\; & k=0,1,2,\cdots ,\\ & A_{\delta_{k}}\in A,\delta_{k}\in D. \end{array} \end{array}$$ (1)

$x\in R^{5}$ is the nonnegative cell type vector; *x* = [*x*_1_, …, *x*_5_]^*T*^ with *x*_*i*_ ≥ 0 for each *i*. If *i* < 5, *x*_*i*_ is the number of live cells in differentiation state *i*. *x*_5_ is the number of dead or dying cells in total. We adopted a *fluid-like* representation of cell populations, where *x*_*i*_ is not necessarily integer-valued [43], to accommodate the limitations of the data which does not distinguish between the live cells and the dying cells occupying a given differentiation state. $A_{\delta }\in A$ is the dynamics matrix for drug δ∈D, where $A\subset R^{5\times 5}$ is the set of dynamics constraints and D is the set of drugs. The dynamics parameters—transition gains, division gains, and death gains—are encoded in the dynamics matrix (S1 Appendix). The discrete-time interval [*k*, *k* + 1) is the duration between two consecutive measurements, or 12 hours.

### System identification

The core numerical problem is to estimate a dynamics matrix for each treatment condition that fits the empirical data well under the form specified by the system model (1). This problem cannot be solved exactly due to the limitations of the data: (i) the data does not distinguish between the live cells and the dying cells occupying a given differentiation state, and (ii) measurements from certain wells at certain time points were not available due to instrument errors. To address the first challenge, we combined the observed numbers of cells in each differentiation state and the observed death fractions into the form of the cell type vector *x*, where death was distributed evenly across the differentiation states in view of the preliminary work (S1 Appendix). To address the second challenge, we inserted these data samples into an alternating minimization (AM) algorithm to obtain an estimate of *A_δ_*, which we refer to as the *AM-optimized dynamics matrix* (${\hat{A}}_{\delta }$). *Alternating minimization* [28] is a local optimization method that reduces the value of a given cost function by alternating the role of the optimization variable between two variables; in our setting, these two variables are a data variable *X* and a dynamics matrix variable A. (Expectation maximization is a special case of alternating minimization [44] [45] [46] [47] [48]). *Initialization for local optimization* [29] was used to help mitigate the possibility of converging to a local minimum that poorly represented the cancer dynamics. Specifically, we initialized the alternating minimization with the dynamics matrix that solved a convex problem exactly within numerical accuracy, where the convex problem approximates our original non-convex problem [29]. This convex problem is the minimization of our cost function in which the data variable was set to an appropriate estimate $\hat{X}$ of its true value. Each column of $\hat{X}$ is a training data sample for a particular time point-well pair, or the sample mean of the available training data for the time point when training data for the time point-well pair was not available. The values of the dynamics parameters converge within numerical accuracy during the iterative process of the alternating minimization algorithm (S3 Appendix). S4 Appendix assesses the sensitivity of the dynamics matrix returned by the algorithm with respect to the initialization of the data variable.

The cost function for the alternating minimization algorithm was designed to reduce measurement error, process error, and estimation error measured in the *l*_2_-norm. This norm was chosen because, as a general measure of length, it is well-suited to identify networks without known structural characteristics, such as sparsity. The penalties applied to measurement error and process error were set equal in view of the preliminary analysis (S1 Appendix). The cost function incorporated *l*_2_-regularization to induce element-wise shrinkage of the dynamics matrix to zero in order to reduce estimation error of the dynamics parameters [49] [50].

### Uncertainty analysis of dynamics parameters

Variations of the dynamics parameters due to measurement error were estimated using the resampling residuals “wild” bootstrap proposed by Wu [30]. We used the resampling method proposed by Davidson and Flachaire [51]. Measurement errors were assumed to be homoskedastic and independent across cell types conditioned on time point and well index in the data generating process. For each treatment condition, 120 bootstrapped dynamics matrices were generated. From these 120 bootstrapped matrices, 120 samples of each dynamics parameter were obtained, and a 95% confidence interval of each parameter was computed by discarding the 3 largest samples and the 3 smallest samples (Fig 4). For each treatment, we also conducted a two-sided one-sample sign test for each dynamics parameter using the corresponding 120 bootstrapped samples; S2 Appendix provides details.

### Comparisons between predictions and data

Methods regarding the computations of data samples, predictions, and p-values in Figs 2, 5, 8 and 9 are provided here. Training or test data samples take the form of *x* specified in (1) and were computed by distributing the observed death fractions evenly across the observed numbers of cells in each differentiation state. Given a dynamics matrix *A*, trajectories predicted by *A* were computed of the form, (*x*_0_, *Ax*_0_, *Ax*_1_, *Ax*_2_, …), where *x*_0_ is a data sample at time 0h, *x*_1_ is a data sample at time 12h, *x*_2_ is a data sample at time 24h, etc. Predictions were chosen to equal the data samples at time 0h. Predictions at time 12h take the form of *Ax*_0_, and predictions at time 24h take the form of *Ax*_1_, etc. *Analysis of variance* (MATLAB function: **anovan**) was used to compute a p-value to quantify the degree of consistency between predictions and data samples over the time horizon starting at time 12h. *Higher* p-values indicate *better* consistency between predictions and data.

### Ensemble model predictions

*Ensemble modeling* was used to evaluate the degree of consistency between predictions and data samples in Figs 2 and 8 (see also Comparisons between Predictions and Data). An ensemble of representative models can be useful for predicting trends when not all parameters are fully constrained by the available data [33] [34] [35] [36]. For a given treatment condition, trajectories predicted by the ensemble of bootstrapped dynamics matrices were computed. At each time point, we computed a 95% confidence interval of the predictions by discarding the 2.5% largest predictions and the 2.5% smallest predictions (rounded down to the nearest integer). Predictions were computed with respect to training data samples in Fig 2 and with respect to test data samples in Fig 8.

### Further investigations of hypotheses

Details regarding Fig 5 are provided below (see also Comparisons between Predictions and Data). The predictions on the left were obtained using the AM-optimized dynamics matrix ${\hat{A}}_{\delta }$ for the particular treatment condition *δ* ∈ {Trametinib, Trametinib+ BEZ235}. The predictions on the right were obtained using a drug-specific dynamics matrix that was trained under additional constraints. Predictions were computed with respect to test data samples. The maximum prediction, the minimum prediction, and the median prediction out of four predictions in total are shown in each plot at each time point.

### Single model predictions

Details regarding Fig 9 are provided below (see also Comparisons between Predictions and Data). A lower-dimensional dynamics matrix was identified via the alternating minimization procedure on the training data with the differentiation-state definitions K14^hi^ and K14^low^, where the observed death was evenly distributed between these two states. The numbers of cells in VIM^hi^K14^low^, K19^hi^VIM^low^K14^low^, and K19^low^VIM^low^K14^low^ were summed to compute the numbers of cells in the K14^low^ state. Predictions by the lower-dimensional dynamics matrix were computed with respect to the test data samples. The maximum prediction, the minimum prediction, and the median prediction out of four predictions in total are shown at each time point.

### Software/Hardware

Computations were executed using commercial software that specializes in linear algebraic computing (MATLAB R2016b, The MathWorks, Inc., Natick, MA). Optimization routines were performed using a convex optimization software package that interfaces with MATLAB (CVX [52], Version 2.1, Build 1116) with the solvers SeDuMi [53] and SDPT3 [54]. Computing was completed on a 64-bit operating system with 16.0 GB RAM, and Intel Core i7-4700MQ CPU @ 2.40GHz processor. Execution time for system identification was roughly one half-hour, and execution time for uncertainty analysis (bootstrapping) was roughly 3 days. Code, training data, and test data are provided in S1 Code/Training Data/Test Data.

## Supporting information

S1 Code/Training Data/Test Data MATLAB Code with Training and Test Data. The experimental data and code used to generate the computational results of this paper are provided. MATLAB (The MathWorks, Inc.) and CVX software [52] are required. The raw training data is in the file Timeseries_Raw_15wells.xlsx, and the raw test data is in the file Timeseries_Raw_4wells.xlsx.
(ZIP)

S1 Table Dynamics parameters. This table provides the values of the dynamics parameters from each drug-specific AM-optimized dynamics matrix.
(XLSX)

S1 Figure EdU-positivity of DMSO-treated HCC1143 cells. The percentages of EdU-positive DMSO-treated HCC1143 cells for each differentiation-state marker expression level are shown. The data were collected via the cell cycle analysis methods of [18].
(TIF)

S2 Figure Single-cell mean-fluorescent intensities of Cytokeratin 19, Cytokeratin 14, and Vimentin in the HCC1143 cell line. HCC1143 cells were treated with either DMSO (gray), 1*μ*M Trametinib (green), or 1*μ*M BEZ235 (blue) for 72h, then fixed and stained with antibodies against Cytokeratin 19 (K19), Cytokeratin 14 (K14), and Vimentin (VIM). Cells were imaged and single-cell mean-fluorescent intensities were calculated using image cytometry software [18] and displayed in a histogram.
(TIF)

S1 Figure Data HCC1143 DMSO EdU data. The data for S1 Figure is provided here.
(XLSX)

S2 Figure Data HCC1143 marker histogram data. The data for S2 Figure is provided here.
(XLSX)

S1 Appendix Mathematical and numerical methods. This document provides additional details on the mathematical and numerical methods.
(PDF)

S2 Appendix P-values for dynamics parameters. This appendix provides the outcome of a two-sided one-sample sign test for each dynamics parameter.
(PDF)

S3 Appendix Evolution of dynamics parameters during alternating minimization. This appendix shows how the values of the dynamics parameters evolve during the iterative process of the alternating minimization algorithm.
(PDF)

S4 Appendix Multi-initialization system identification. This appendix assesses the sensitivity of the dynamics matrix returned by the alternating minimization algorithm with respect to the initialization.
(PDF)
