# Correction: Modeling differentiation-state transitions linked to therapeutic escape in triple-negative breast cancer

**DOI:** 10.1371/journal.pcbi.1007441

**Published:** 2019-10-09

**Authors:** Margaret P. Chapman, Tyler Risom, Anil J. Aswani, Ellen M. Langer, Rosalie C. Sears, Claire J. Tomlin

In the funding statement the following sentence was omitted:

“This work was supported in part by NCI Cancer Systems Biology Center U54 CA209988.”

The correct funding statement is as follows:

MPC is supported by a National Science Foundation Graduate Research Fellowship (www.nsfgrfp.org) and was supported by the Berkeley Fellowship for Graduate Study (grad.berkeley.edu) and the Tau Beta Pi Engineering Honors Society, Williams No. 35 (www.tbp.org). CJT and MPC were supported by the National Institutes of Health (NIH) Center “Systems Biology of Collective Cells Decisions” through Stanford University NIH #P50GM107615, and by the National Cancer Institute (NCI) CSBC consortia “Model-Based Predictions of Responses to RTK Pathway Therapies” through OHSU NCI #U54CA112970 and “Measuring, Modeling and Controlling Heterogeneity” through OHSU NCI #1U54CA209988-01A1. TR was supported by the Ruth L. Kirschstein T32 Program in Molecular and Cellular Biosciences Training Grant 5T32GM071338-09, Vertex Pharmaceuticals Scholarship (www.vrtx.com), and Tartar Trust Fellowship (www.ohsu.edu). EML was supported by the American Cancer Society Postdoctoral Fellowship (www.cancer.org). RCS is supported by the National Institutes of Health, National Cancer Institute R01-CA196228, R01-CA186241, and U54-CA209988, the Department of Defense Breast Cancer Research Program BC160550P1, the Colson Family Foundation (Vancouver, WA), and the Prospect Creek Foundation (Minneapolis, MN). This work was supported in part by NCI Cancer Systems Biology Center U54 CA209988. The funders had no role in study design, data collection and analysis, decision to publish, or preparation of the manuscript.
