# Supplementary material for: Modeling differentiation-state transitions linked to therapeutic escape in triple-negative breast cancer
Source: PLoS Comput Biol. 2019 Mar 11;15(3):e1006840. doi: 10.1371/journal.pcbi.1006840 (PMC6428348; doi:10.1371/journal.pcbi.1006840)
Supplement: S1 Code/Training Data/Test Data — The experimental data and code used to generate the computational results of this paper are provided. MATLAB (The MathWorks, Inc.) and CVX software [52] are required. The raw training data is in the file Timeseries_Raw_15wells.xlsx, and the raw test data is in the file Timeseries_Raw_4wells.xlsx. (ZIP) [file pcbi.1006840.s001.zip › S1_Code_TrainData_TestData/Results_Summer_2017/bootstrap/command_window_bootstrap_aug6.docx]

>> load('model_id_K14VIMK19_july31.mat')

cv : 1 out of 7 done. AM iter = 190

cv : 2 out of 7 done. AM iter = 186

cv : 3 out of 7 done. AM iter = 685

cv : 4 out of 7 done. AM iter = 927

cv : 5 out of 7 done. AM iter = 658

cv : 6 out of 7 done. AM iter = 575

cv : 7 out of 7 done. AM iter = 62

model id done, even death, mu = lambda, lambda* = 0.005. AM iter = 444

DMSO BOOT iter 1 done out of 120

cv : 1 out of 7 done. AM iter = 96

cv : 2 out of 7 done. AM iter = 94

cv : 3 out of 7 done. AM iter = 82

cv : 4 out of 7 done. AM iter = 72

cv : 5 out of 7 done. AM iter = 408

cv : 6 out of 7 done. AM iter = 657

cv : 7 out of 7 done. AM iter = 62

model id done, even death, mu = lambda, lambda* = 0.005. AM iter = 61

DMSO BOOT iter 2 done out of 120

cv : 1 out of 7 done. AM iter = 71

cv : 2 out of 7 done. AM iter = 71

cv : 3 out of 7 done. AM iter = 71

cv : 4 out of 7 done. AM iter = 71

cv : 5 out of 7 done. AM iter = 547

cv : 6 out of 7 done. AM iter = 360

cv : 7 out of 7 done. AM iter = 66

model id done, even death, mu = lambda, lambda* = 0.001. AM iter = 53

DMSO BOOT iter 3 done out of 120

cv : 1 out of 7 done. AM iter = 138

cv : 2 out of 7 done. AM iter = 134

cv : 3 out of 7 done. AM iter = 111

cv : 4 out of 7 done. AM iter = 91

cv : 5 out of 7 done. AM iter = 97

cv : 6 out of 7 done. AM iter = 85

cv : 7 out of 7 done. AM iter = 145

model id done, even death, mu = lambda, lambda* = 0.005. AM iter = 269

DMSO BOOT iter 4 done out of 120

cv : 1 out of 7 done. AM iter = 106

cv : 2 out of 7 done. AM iter = 106

cv : 3 out of 7 done. AM iter = 186

cv : 4 out of 7 done. AM iter = 122

cv : 5 out of 7 done. AM iter = 461

cv : 6 out of 7 done. AM iter = 358

cv : 7 out of 7 done. AM iter = 68

model id done, even death, mu = lambda, lambda* = 0.1. AM iter = 99

DMSO BOOT iter 5 done out of 120

cv : 1 out of 7 done. AM iter = 113

cv : 2 out of 7 done. AM iter = 145

cv : 3 out of 7 done. AM iter = 350

cv : 4 out of 7 done. AM iter = 157

cv : 5 out of 7 done. AM iter = 86

cv : 6 out of 7 done. AM iter = 213

cv : 7 out of 7 done. AM iter = 62

model id done, even death, mu = lambda, lambda* = 0.05. AM iter = 184

DMSO BOOT iter 6 done out of 120

cv : 1 out of 7 done. AM iter = 86

cv : 2 out of 7 done. AM iter = 86

cv : 3 out of 7 done. AM iter = 85

cv : 4 out of 7 done. AM iter = 91

cv : 5 out of 7 done. AM iter = 173

cv : 6 out of 7 done. AM iter = 160

cv : 7 out of 7 done. AM iter = 50

model id done, even death, mu = lambda, lambda* = 0.001. AM iter = 88

DMSO BOOT iter 7 done out of 120

cv : 1 out of 7 done. AM iter = 84

cv : 2 out of 7 done. AM iter = 84

cv : 3 out of 7 done. AM iter = 87

cv : 4 out of 7 done. AM iter = 95

cv : 5 out of 7 done. AM iter = 163

cv : 6 out of 7 done. AM iter = 137

cv : 7 out of 7 done. AM iter = 54

model id done, even death, mu = lambda, lambda* = 0.005. AM iter = 139

DMSO BOOT iter 8 done out of 120

cv : 1 out of 7 done. AM iter = 83

cv : 2 out of 7 done. AM iter = 82

cv : 3 out of 7 done. AM iter = 80

cv : 4 out of 7 done. AM iter = 114

cv : 5 out of 7 done. AM iter = 127

cv : 6 out of 7 done. AM iter = 87

cv : 7 out of 7 done. AM iter = 36

model id done, even death, mu = lambda, lambda* = 0.005. AM iter = 127

DMSO BOOT iter 9 done out of 120

cv : 1 out of 7 done. AM iter = 188

cv : 2 out of 7 done. AM iter = 168

cv : 3 out of 7 done. AM iter = 207

cv : 4 out of 7 done. AM iter = 187

cv : 5 out of 7 done. AM iter = 247

cv : 6 out of 7 done. AM iter = 633

cv : 7 out of 7 done. AM iter = 49

model id done, even death, mu = lambda, lambda* = 0.01. AM iter = 484

DMSO BOOT iter 10 done out of 120

cv : 1 out of 7 done. AM iter = 101

cv : 2 out of 7 done. AM iter = 101

cv : 3 out of 7 done. AM iter = 100

cv : 4 out of 7 done. AM iter = 100

cv : 5 out of 7 done. AM iter = 223

cv : 6 out of 7 done. AM iter = 113

cv : 7 out of 7 done. AM iter = 49

model id done, even death, mu = lambda, lambda* = 0.01. AM iter = 193

DMSO BOOT iter 11 done out of 120

cv : 1 out of 7 done. AM iter = 73

cv : 2 out of 7 done. AM iter = 73

cv : 3 out of 7 done. AM iter = 72

cv : 4 out of 7 done. AM iter = 73

cv : 5 out of 7 done. AM iter = 45

cv : 6 out of 7 done. AM iter = 52

cv : 7 out of 7 done. AM iter = 174

model id done, even death, mu = lambda, lambda* = 0.01. AM iter = 35

DMSO BOOT iter 12 done out of 120

cv : 1 out of 7 done. AM iter = 47

cv : 2 out of 7 done. AM iter = 47

cv : 3 out of 7 done. AM iter = 47

cv : 4 out of 7 done. AM iter = 47

cv : 5 out of 7 done. AM iter = 122

cv : 6 out of 7 done. AM iter = 71

cv : 7 out of 7 done. AM iter = 36

model id done, even death, mu = lambda, lambda* = 0.01. AM iter = 112

DMSO BOOT iter 13 done out of 120

cv : 1 out of 7 done. AM iter = 92

cv : 2 out of 7 done. AM iter = 92

cv : 3 out of 7 done. AM iter = 91

cv : 4 out of 7 done. AM iter = 95

cv : 5 out of 7 done. AM iter = 122

cv : 6 out of 7 done. AM iter = 120

cv : 7 out of 7 done. AM iter = 56

model id done, even death, mu = lambda, lambda* = 0.005. AM iter = 137

DMSO BOOT iter 14 done out of 120

cv : 1 out of 7 done. AM iter = 457

cv : 2 out of 7 done. AM iter = 408

cv : 3 out of 7 done. AM iter = 242

cv : 4 out of 7 done. AM iter = 234

cv : 5 out of 7 done. AM iter = 92

cv : 6 out of 7 done. AM iter = 83

cv : 7 out of 7 done. AM iter = 101

model id done, even death, mu = lambda, lambda* = 0.001. AM iter = 92

DMSO BOOT iter 15 done out of 120

cv : 1 out of 7 done. AM iter = 86

cv : 2 out of 7 done. AM iter = 86

cv : 3 out of 7 done. AM iter = 85

cv : 4 out of 7 done. AM iter = 85

cv : 5 out of 7 done. AM iter = 188

cv : 6 out of 7 done. AM iter = 168

cv : 7 out of 7 done. AM iter = 50

model id done, even death, mu = lambda, lambda* = 0.001. AM iter = 56

DMSO BOOT iter 16 done out of 120

cv : 1 out of 7 done. AM iter = 497

cv : 2 out of 7 done. AM iter = 498

cv : 3 out of 7 done. AM iter = 140

cv : 4 out of 7 done. AM iter = 131

cv : 5 out of 7 done. AM iter = 68

cv : 6 out of 7 done. AM iter = 65

cv : 7 out of 7 done. AM iter = 65

model id done, even death, mu = lambda, lambda* = 0.01. AM iter = 185

DMSO BOOT iter 17 done out of 120

cv : 1 out of 7 done. AM iter = 97

cv : 2 out of 7 done. AM iter = 100

cv : 3 out of 7 done. AM iter = 119

cv : 4 out of 7 done. AM iter = 197

cv : 5 out of 7 done. AM iter = 124

cv : 6 out of 7 done. AM iter = 89

cv : 7 out of 7 done. AM iter = 32

model id done, even death, mu = lambda, lambda* = 0.005. AM iter = 107

DMSO BOOT iter 18 done out of 120

cv : 1 out of 7 done. AM iter = 69

cv : 2 out of 7 done. AM iter = 68

cv : 3 out of 7 done. AM iter = 66

cv : 4 out of 7 done. AM iter = 66

cv : 5 out of 7 done. AM iter = 87

cv : 6 out of 7 done. AM iter = 74

cv : 7 out of 7 done. AM iter = 35

model id done, even death, mu = lambda, lambda* = 0.01. AM iter = 100

DMSO BOOT iter 19 done out of 120

cv : 1 out of 7 done. AM iter = 89

cv : 2 out of 7 done. AM iter = 90

cv : 3 out of 7 done. AM iter = 97

cv : 4 out of 7 done. AM iter = 128

cv : 5 out of 7 done. AM iter = 189

cv : 6 out of 7 done. AM iter = 166

cv : 7 out of 7 done. AM iter = 59

model id done, even death, mu = lambda, lambda* = 0.01. AM iter = 453

DMSO BOOT iter 20 done out of 120

cv : 1 out of 7 done. AM iter = 80

cv : 2 out of 7 done. AM iter = 82

cv : 3 out of 7 done. AM iter = 101

cv : 4 out of 7 done. AM iter = 181

cv : 5 out of 7 done. AM iter = 62

cv : 6 out of 7 done. AM iter = 66

cv : 7 out of 7 done. AM iter = 157

model id done, even death, mu = lambda, lambda* = 0.01. AM iter = 232

DMSO BOOT iter 21 done out of 120

cv : 1 out of 7 done. AM iter = 67

cv : 2 out of 7 done. AM iter = 67

cv : 3 out of 7 done. AM iter = 76

cv : 4 out of 7 done. AM iter = 76

cv : 5 out of 7 done. AM iter = 74

cv : 6 out of 7 done. AM iter = 49

cv : 7 out of 7 done. AM iter = 31

model id done, even death, mu = lambda, lambda* = 0.01. AM iter = 67

DMSO BOOT iter 22 done out of 120

cv : 1 out of 7 done. AM iter = 86

cv : 2 out of 7 done. AM iter = 86

cv : 3 out of 7 done. AM iter = 87

cv : 4 out of 7 done. AM iter = 131

cv : 5 out of 7 done. AM iter = 162

cv : 6 out of 7 done. AM iter = 163

cv : 7 out of 7 done. AM iter = 58

model id done, even death, mu = lambda, lambda* = 0.001. AM iter = 47

DMSO BOOT iter 23 done out of 120

cv : 1 out of 7 done. AM iter = 99

cv : 2 out of 7 done. AM iter = 109

cv : 3 out of 7 done. AM iter = 122

cv : 4 out of 7 done. AM iter = 180

cv : 5 out of 7 done. AM iter = 222

cv : 6 out of 7 done. AM iter = 182

cv : 7 out of 7 done. AM iter = 59

model id done, even death, mu = lambda, lambda* = 0.01. AM iter = 143

DMSO BOOT iter 24 done out of 120

cv : 1 out of 7 done. AM iter = 86

cv : 2 out of 7 done. AM iter = 95

cv : 3 out of 7 done. AM iter = 87

cv : 4 out of 7 done. AM iter = 75

cv : 5 out of 7 done. AM iter = 476

cv : 6 out of 7 done. AM iter = 59

cv : 7 out of 7 done. AM iter = 34

model id done, even death, mu = lambda, lambda* = 0.005. AM iter = 74

DMSO BOOT iter 25 done out of 120

cv : 1 out of 7 done. AM iter = 125

cv : 2 out of 7 done. AM iter = 122

cv : 3 out of 7 done. AM iter = 114

cv : 4 out of 7 done. AM iter = 105

cv : 5 out of 7 done. AM iter = 63

cv : 6 out of 7 done. AM iter = 54

cv : 7 out of 7 done. AM iter = 31

model id done, even death, mu = lambda, lambda* = 0.005. AM iter = 130

DMSO BOOT iter 26 done out of 120

cv : 1 out of 7 done. AM iter = 81

cv : 2 out of 7 done. AM iter = 77

cv : 3 out of 7 done. AM iter = 238

cv : 4 out of 7 done. AM iter = 119

cv : 5 out of 7 done. AM iter = 107

cv : 6 out of 7 done. AM iter = 103

cv : 7 out of 7 done. AM iter = 38

model id done, even death, mu = lambda, lambda* = 0.005. AM iter = 167

DMSO BOOT iter 27 done out of 120

cv : 1 out of 7 done. AM iter = 63

cv : 2 out of 7 done. AM iter = 63

cv : 3 out of 7 done. AM iter = 68

cv : 4 out of 7 done. AM iter = 121

cv : 5 out of 7 done. AM iter = 117

cv : 6 out of 7 done. AM iter = 117

cv : 7 out of 7 done. AM iter = 44

model id done, even death, mu = lambda, lambda* = 0.001. AM iter = 55

DMSO BOOT iter 28 done out of 120

cv : 1 out of 7 done. AM iter = 196

cv : 2 out of 7 done. AM iter = 185

cv : 3 out of 7 done. AM iter = 212

cv : 4 out of 7 done. AM iter = 118

cv : 5 out of 7 done. AM iter = 75

cv : 6 out of 7 done. AM iter = 62

cv : 7 out of 7 done. AM iter = 179

model id done, even death, mu = lambda, lambda* = 0.001. AM iter = 167

DMSO BOOT iter 29 done out of 120

cv : 1 out of 7 done. AM iter = 85

cv : 2 out of 7 done. AM iter = 85

cv : 3 out of 7 done. AM iter = 85

cv : 4 out of 7 done. AM iter = 86

cv : 5 out of 7 done. AM iter = 73

cv : 6 out of 7 done. AM iter = 75

cv : 7 out of 7 done. AM iter = 107

model id done, even death, mu = lambda, lambda* = 0.01. AM iter = 79

DMSO BOOT iter 30 done out of 120

cv : 1 out of 7 done. AM iter = 65

cv : 2 out of 7 done. AM iter = 67

cv : 3 out of 7 done. AM iter = 75

cv : 4 out of 7 done. AM iter = 82

cv : 5 out of 7 done. AM iter = 254

cv : 6 out of 7 done. AM iter = 309

cv : 7 out of 7 done. AM iter = 129

model id done, even death, mu = lambda, lambda* = 0.005. AM iter = 356

DMSO BOOT iter 31 done out of 120

cv : 1 out of 7 done. AM iter = 123

cv : 2 out of 7 done. AM iter = 128

cv : 3 out of 7 done. AM iter = 197

cv : 4 out of 7 done. AM iter = 199

cv : 5 out of 7 done. AM iter = 96

cv : 6 out of 7 done. AM iter = 73

cv : 7 out of 7 done. AM iter = 85

model id done, even death, mu = lambda, lambda* = 0.01. AM iter = 144

DMSO BOOT iter 32 done out of 120

cv : 1 out of 7 done. AM iter = 282

cv : 2 out of 7 done. AM iter = 228

cv : 3 out of 7 done. AM iter = 113

cv : 4 out of 7 done. AM iter = 122

cv : 5 out of 7 done. AM iter = 131

cv : 6 out of 7 done. AM iter = 66

cv : 7 out of 7 done. AM iter = 38

model id done, even death, mu = lambda, lambda* = 0.001. AM iter = 44

DMSO BOOT iter 33 done out of 120

cv : 1 out of 7 done. AM iter = 160

cv : 2 out of 7 done. AM iter = 150

cv : 3 out of 7 done. AM iter = 109

cv : 4 out of 7 done. AM iter = 95

cv : 5 out of 7 done. AM iter = 112

cv : 6 out of 7 done. AM iter = 310

cv : 7 out of 7 done. AM iter = 95

model id done, even death, mu = lambda, lambda* = 0.005. AM iter = 100

DMSO BOOT iter 34 done out of 120

cv : 1 out of 7 done. AM iter = 94

cv : 2 out of 7 done. AM iter = 92

cv : 3 out of 7 done. AM iter = 143

cv : 4 out of 7 done. AM iter = 111

cv : 5 out of 7 done. AM iter = 427

cv : 6 out of 7 done. AM iter = 1000

cv : 7 out of 7 done. AM iter = 106

model id done, even death, mu = lambda, lambda* = 0.001. AM iter = 98

DMSO BOOT iter 35 done out of 120

cv : 1 out of 7 done. AM iter = 247

cv : 2 out of 7 done. AM iter = 227

cv : 3 out of 7 done. AM iter = 172

cv : 4 out of 7 done. AM iter = 144

cv : 5 out of 7 done. AM iter = 976

cv : 6 out of 7 done. AM iter = 146

cv : 7 out of 7 done. AM iter = 64

model id done, even death, mu = lambda, lambda* = 0.005. AM iter = 245

DMSO BOOT iter 36 done out of 120

cv : 1 out of 7 done. AM iter = 53

cv : 2 out of 7 done. AM iter = 53

cv : 3 out of 7 done. AM iter = 48

cv : 4 out of 7 done. AM iter = 117

cv : 5 out of 7 done. AM iter = 45

cv : 6 out of 7 done. AM iter = 35

cv : 7 out of 7 done. AM iter = 114

model id done, even death, mu = lambda, lambda* = 0.01. AM iter = 77

DMSO BOOT iter 37 done out of 120

cv : 1 out of 7 done. AM iter = 96

cv : 2 out of 7 done. AM iter = 96

cv : 3 out of 7 done. AM iter = 104

cv : 4 out of 7 done. AM iter = 159

cv : 5 out of 7 done. AM iter = 44

cv : 6 out of 7 done. AM iter = 80

cv : 7 out of 7 done. AM iter = 153

model id done, even death, mu = lambda, lambda* = 0.01. AM iter = 66

DMSO BOOT iter 38 done out of 120

cv : 1 out of 7 done. AM iter = 155

cv : 2 out of 7 done. AM iter = 136

cv : 3 out of 7 done. AM iter = 105

cv : 4 out of 7 done. AM iter = 98

cv : 5 out of 7 done. AM iter = 39

cv : 6 out of 7 done. AM iter = 41

cv : 7 out of 7 done. AM iter = 316

model id done, even death, mu = lambda, lambda* = 0.01. AM iter = 52

DMSO BOOT iter 39 done out of 120

cv : 1 out of 7 done. AM iter = 120

cv : 2 out of 7 done. AM iter = 119

cv : 3 out of 7 done. AM iter = 111

cv : 4 out of 7 done. AM iter = 151

cv : 5 out of 7 done. AM iter = 185

cv : 6 out of 7 done. AM iter = 185

cv : 7 out of 7 done. AM iter = 77

model id done, even death, mu = lambda, lambda* = 0.05. AM iter = 160

DMSO BOOT iter 40 done out of 120

cv : 1 out of 7 done. AM iter = 75

cv : 2 out of 7 done. AM iter = 74

cv : 3 out of 7 done. AM iter = 111

cv : 4 out of 7 done. AM iter = 79

cv : 5 out of 7 done. AM iter = 264

cv : 6 out of 7 done. AM iter = 207

cv : 7 out of 7 done. AM iter = 41

model id done, even death, mu = lambda, lambda* = 0.01. AM iter = 293

DMSO BOOT iter 41 done out of 120

cv : 1 out of 7 done. AM iter = 252

cv : 2 out of 7 done. AM iter = 246

cv : 3 out of 7 done. AM iter = 217

cv : 4 out of 7 done. AM iter = 529

cv : 5 out of 7 done. AM iter = 193

cv : 6 out of 7 done. AM iter = 152

cv : 7 out of 7 done. AM iter = 57

model id done, even death, mu = lambda, lambda* = 0.005. AM iter = 112

DMSO BOOT iter 42 done out of 120

cv : 1 out of 7 done. AM iter = 86

cv : 2 out of 7 done. AM iter = 86

cv : 3 out of 7 done. AM iter = 88

cv : 4 out of 7 done. AM iter = 83

cv : 5 out of 7 done. AM iter = 180

cv : 6 out of 7 done. AM iter = 153

cv : 7 out of 7 done. AM iter = 87

model id done, even death, mu = lambda, lambda* = 0.01. AM iter = 80

DMSO BOOT iter 43 done out of 120

cv : 1 out of 7 done. AM iter = 64

cv : 2 out of 7 done. AM iter = 64

cv : 3 out of 7 done. AM iter = 71

cv : 4 out of 7 done. AM iter = 180

cv : 5 out of 7 done. AM iter = 118

cv : 6 out of 7 done. AM iter = 96

cv : 7 out of 7 done. AM iter = 34

model id done, even death, mu = lambda, lambda* = 0.005. AM iter = 106

DMSO BOOT iter 44 done out of 120

cv : 1 out of 7 done. AM iter = 83

cv : 2 out of 7 done. AM iter = 83

cv : 3 out of 7 done. AM iter = 114

cv : 4 out of 7 done. AM iter = 90

cv : 5 out of 7 done. AM iter = 84

cv : 6 out of 7 done. AM iter = 72

cv : 7 out of 7 done. AM iter = 80

model id done, even death, mu = lambda, lambda* = 0.01. AM iter = 54

DMSO BOOT iter 45 done out of 120

cv : 1 out of 7 done. AM iter = 133

cv : 2 out of 7 done. AM iter = 134

cv : 3 out of 7 done. AM iter = 355

cv : 4 out of 7 done. AM iter = 137

cv : 5 out of 7 done. AM iter = 184

cv : 6 out of 7 done. AM iter = 439

cv : 7 out of 7 done. AM iter = 45

model id done, even death, mu = lambda, lambda* = 0.01. AM iter = 1000

DMSO BOOT iter 46 done out of 120

cv : 1 out of 7 done. AM iter = 406

cv : 2 out of 7 done. AM iter = 143

cv : 3 out of 7 done. AM iter = 253

cv : 4 out of 7 done. AM iter = 33

cv : 5 out of 7 done. AM iter = 39

cv : 6 out of 7 done. AM iter = 45

cv : 7 out of 7 done. AM iter = 309

model id done, even death, mu = lambda, lambda* = 0.05. AM iter = 1000

DMSO BOOT iter 47 done out of 120

cv : 1 out of 7 done. AM iter = 53

cv : 2 out of 7 done. AM iter = 53

cv : 3 out of 7 done. AM iter = 50

cv : 4 out of 7 done. AM iter = 49

cv : 5 out of 7 done. AM iter = 311

cv : 6 out of 7 done. AM iter = 388

cv : 7 out of 7 done. AM iter = 65

model id done, even death, mu = lambda, lambda* = 0.05. AM iter = 61

DMSO BOOT iter 48 done out of 120

cv : 1 out of 7 done. AM iter = 70

cv : 2 out of 7 done. AM iter = 68

cv : 3 out of 7 done. AM iter = 140

cv : 4 out of 7 done. AM iter = 137

cv : 5 out of 7 done. AM iter = 105

cv : 6 out of 7 done. AM iter = 99

cv : 7 out of 7 done. AM iter = 47

model id done, even death, mu = lambda, lambda* = 0.01. AM iter = 129

DMSO BOOT iter 49 done out of 120

cv : 1 out of 7 done. AM iter = 140

cv : 2 out of 7 done. AM iter = 138

cv : 3 out of 7 done. AM iter = 124

cv : 4 out of 7 done. AM iter = 113

cv : 5 out of 7 done. AM iter = 59

cv : 6 out of 7 done. AM iter = 49

cv : 7 out of 7 done. AM iter = 122

model id done, even death, mu = lambda, lambda* = 0.01. AM iter = 54

DMSO BOOT iter 50 done out of 120

cv : 1 out of 7 done. AM iter = 56

cv : 2 out of 7 done. AM iter = 58

cv : 3 out of 7 done. AM iter = 825

cv : 4 out of 7 done. AM iter = 311

cv : 5 out of 7 done. AM iter = 329

cv : 6 out of 7 done. AM iter = 265

cv : 7 out of 7 done. AM iter = 87

model id done, even death, mu = lambda, lambda* = 1. AM iter = 58

DMSO BOOT iter 51 done out of 120

cv : 1 out of 7 done. AM iter = 57

cv : 2 out of 7 done. AM iter = 57

cv : 3 out of 7 done. AM iter = 57

cv : 4 out of 7 done. AM iter = 57

cv : 5 out of 7 done. AM iter = 145

cv : 6 out of 7 done. AM iter = 141

cv : 7 out of 7 done. AM iter = 50

model id done, even death, mu = lambda, lambda* = 0.005. AM iter = 289

DMSO BOOT iter 52 done out of 120

cv : 1 out of 7 done. AM iter = 75

cv : 2 out of 7 done. AM iter = 75

cv : 3 out of 7 done. AM iter = 75

cv : 4 out of 7 done. AM iter = 75

cv : 5 out of 7 done. AM iter = 82

cv : 6 out of 7 done. AM iter = 80

cv : 7 out of 7 done. AM iter = 37

model id done, even death, mu = lambda, lambda* = 0.05. AM iter = 77

DMSO BOOT iter 53 done out of 120

cv : 1 out of 7 done. AM iter = 544

cv : 2 out of 7 done. AM iter = 501

cv : 3 out of 7 done. AM iter = 447

cv : 4 out of 7 done. AM iter = 183

cv : 5 out of 7 done. AM iter = 1

cv : 6 out of 7 done. AM iter = 78

cv : 7 out of 7 done. AM iter = 170

model id done, even death, mu = lambda, lambda* = 0.05. AM iter = 71

DMSO BOOT iter 54 done out of 120

cv : 1 out of 7 done. AM iter = 90

cv : 2 out of 7 done. AM iter = 88

cv : 3 out of 7 done. AM iter = 77

cv : 4 out of 7 done. AM iter = 86

cv : 5 out of 7 done. AM iter = 180

cv : 6 out of 7 done. AM iter = 156

cv : 7 out of 7 done. AM iter = 43

model id done, even death, mu = lambda, lambda* = 1. AM iter = 197

DMSO BOOT iter 55 done out of 120

cv : 1 out of 7 done. AM iter = 83

cv : 2 out of 7 done. AM iter = 82

cv : 3 out of 7 done. AM iter = 72

cv : 4 out of 7 done. AM iter = 58

cv : 5 out of 7 done. AM iter = 49

cv : 6 out of 7 done. AM iter = 1

cv : 7 out of 7 done. AM iter = 150

model id done, even death, mu = lambda, lambda* = 0.005. AM iter = 1

DMSO BOOT iter 56 done out of 120

cv : 1 out of 7 done. AM iter = 71

cv : 2 out of 7 done. AM iter = 71

cv : 3 out of 7 done. AM iter = 71

cv : 4 out of 7 done. AM iter = 73

cv : 5 out of 7 done. AM iter = 379

cv : 6 out of 7 done. AM iter = 465

cv : 7 out of 7 done. AM iter = 59

model id done, even death, mu = lambda, lambda* = 0.01. AM iter = 76

DMSO BOOT iter 57 done out of 120

cv : 1 out of 7 done. AM iter = 81

cv : 2 out of 7 done. AM iter = 81

cv : 3 out of 7 done. AM iter = 81

cv : 4 out of 7 done. AM iter = 137

cv : 5 out of 7 done. AM iter = 394

cv : 6 out of 7 done. AM iter = 422

cv : 7 out of 7 done. AM iter = 59

model id done, even death, mu = lambda, lambda* = 0.001. AM iter = 63

DMSO BOOT iter 58 done out of 120

cv : 1 out of 7 done. AM iter = 75

cv : 2 out of 7 done. AM iter = 68

cv : 3 out of 7 done. AM iter = 66

cv : 4 out of 7 done. AM iter = 63

cv : 5 out of 7 done. AM iter = 28

cv : 6 out of 7 done. AM iter = 46

cv : 7 out of 7 done. AM iter = 242

model id done, even death, mu = lambda, lambda* = 0.005. AM iter = 138

DMSO BOOT iter 59 done out of 120

cv : 1 out of 7 done. AM iter = 163

cv : 2 out of 7 done. AM iter = 158

cv : 3 out of 7 done. AM iter = 154

cv : 4 out of 7 done. AM iter = 203

cv : 5 out of 7 done. AM iter = 69

cv : 6 out of 7 done. AM iter = 80

cv : 7 out of 7 done. AM iter = 67

model id done, even death, mu = lambda, lambda* = 0.05. AM iter = 63

DMSO BOOT iter 60 done out of 120

cv : 1 out of 7 done. AM iter = 118

cv : 2 out of 7 done. AM iter = 115

cv : 3 out of 7 done. AM iter = 63

cv : 4 out of 7 done. AM iter = 60

cv : 5 out of 7 done. AM iter = 136

cv : 6 out of 7 done. AM iter = 92

cv : 7 out of 7 done. AM iter = 66

model id done, even death, mu = lambda, lambda* = 0.05. AM iter = 101

DMSO BOOT iter 61 done out of 120

cv : 1 out of 7 done. AM iter = 110

cv : 2 out of 7 done. AM iter = 109

cv : 3 out of 7 done. AM iter = 104

cv : 4 out of 7 done. AM iter = 100

cv : 5 out of 7 done. AM iter = 575

cv : 6 out of 7 done. AM iter = 319

cv : 7 out of 7 done. AM iter = 52

model id done, even death, mu = lambda, lambda* = 0.01. AM iter = 475

DMSO BOOT iter 62 done out of 120

cv : 1 out of 7 done. AM iter = 168

cv : 2 out of 7 done. AM iter = 164

cv : 3 out of 7 done. AM iter = 145

cv : 4 out of 7 done. AM iter = 374

cv : 5 out of 7 done. AM iter = 330

cv : 6 out of 7 done. AM iter = 325

cv : 7 out of 7 done. AM iter = 84

model id done, even death, mu = lambda, lambda* = 0.001. AM iter = 141

DMSO BOOT iter 63 done out of 120

cv : 1 out of 7 done. AM iter = 113

cv : 2 out of 7 done. AM iter = 112

cv : 3 out of 7 done. AM iter = 104

cv : 4 out of 7 done. AM iter = 87

cv : 5 out of 7 done. AM iter = 1

cv : 6 out of 7 done. AM iter = 31

cv : 7 out of 7 done. AM iter = 120

model id done, even death, mu = lambda, lambda* = 0.01. AM iter = 1

DMSO BOOT iter 64 done out of 120

cv : 1 out of 7 done. AM iter = 110

cv : 2 out of 7 done. AM iter = 110

cv : 3 out of 7 done. AM iter = 112

cv : 4 out of 7 done. AM iter = 518

cv : 5 out of 7 done. AM iter = 155

cv : 6 out of 7 done. AM iter = 136

cv : 7 out of 7 done. AM iter = 49

model id done, even death, mu = lambda, lambda* = 0.01. AM iter = 329

DMSO BOOT iter 65 done out of 120

cv : 1 out of 7 done. AM iter = 109

cv : 2 out of 7 done. AM iter = 107

cv : 3 out of 7 done. AM iter = 138

cv : 4 out of 7 done. AM iter = 223

cv : 5 out of 7 done. AM iter = 163

cv : 6 out of 7 done. AM iter = 93

cv : 7 out of 7 done. AM iter = 40

model id done, even death, mu = lambda, lambda* = 0.005. AM iter = 146

DMSO BOOT iter 66 done out of 120

cv : 1 out of 7 done. AM iter = 30

cv : 2 out of 7 done. AM iter = 32

cv : 3 out of 7 done. AM iter = 42

cv : 4 out of 7 done. AM iter = 61

cv : 5 out of 7 done. AM iter = 1000

cv : 6 out of 7 done. AM iter = 811

cv : 7 out of 7 done. AM iter = 93

model id done, even death, mu = lambda, lambda* = 0.005. AM iter = 365

DMSO BOOT iter 67 done out of 120

cv : 1 out of 7 done. AM iter = 96

cv : 2 out of 7 done. AM iter = 95

cv : 3 out of 7 done. AM iter = 91

cv : 4 out of 7 done. AM iter = 86

cv : 5 out of 7 done. AM iter = 136

cv : 6 out of 7 done. AM iter = 107

cv : 7 out of 7 done. AM iter = 38

model id done, even death, mu = lambda, lambda* = 0.005. AM iter = 144

DMSO BOOT iter 68 done out of 120

cv : 1 out of 7 done. AM iter = 101

cv : 2 out of 7 done. AM iter = 102

cv : 3 out of 7 done. AM iter = 109

cv : 4 out of 7 done. AM iter = 134

cv : 5 out of 7 done. AM iter = 140

cv : 6 out of 7 done. AM iter = 125

cv : 7 out of 7 done. AM iter = 50

model id done, even death, mu = lambda, lambda* = 0.01. AM iter = 157

DMSO BOOT iter 69 done out of 120

cv : 1 out of 7 done. AM iter = 87

cv : 2 out of 7 done. AM iter = 88

cv : 3 out of 7 done. AM iter = 234

cv : 4 out of 7 done. AM iter = 90

cv : 5 out of 7 done. AM iter = 1000

cv : 6 out of 7 done. AM iter = 1000

cv : 7 out of 7 done. AM iter = 94

model id done, even death, mu = lambda, lambda* = 0.005. AM iter = 1000

DMSO BOOT iter 70 done out of 120

cv : 1 out of 7 done. AM iter = 65

cv : 2 out of 7 done. AM iter = 65

cv : 3 out of 7 done. AM iter = 73

cv : 4 out of 7 done. AM iter = 67

cv : 5 out of 7 done. AM iter = 505

cv : 6 out of 7 done. AM iter = 980

cv : 7 out of 7 done. AM iter = 58

model id done, even death, mu = lambda, lambda* = 0.01. AM iter = 711

DMSO BOOT iter 71 done out of 120

cv : 1 out of 7 done. AM iter = 67

cv : 2 out of 7 done. AM iter = 67

cv : 3 out of 7 done. AM iter = 67

cv : 4 out of 7 done. AM iter = 67

cv : 5 out of 7 done. AM iter = 112

cv : 6 out of 7 done. AM iter = 61

cv : 7 out of 7 done. AM iter = 27

model id done, even death, mu = lambda, lambda* = 0.005. AM iter = 105

DMSO BOOT iter 72 done out of 120

cv : 1 out of 7 done. AM iter = 75

cv : 2 out of 7 done. AM iter = 76

cv : 3 out of 7 done. AM iter = 81

cv : 4 out of 7 done. AM iter = 88

cv : 5 out of 7 done. AM iter = 183

cv : 6 out of 7 done. AM iter = 151

cv : 7 out of 7 done. AM iter = 84

model id done, even death, mu = lambda, lambda* = 0.005. AM iter = 140

DMSO BOOT iter 73 done out of 120

cv : 1 out of 7 done. AM iter = 73

cv : 2 out of 7 done. AM iter = 73

cv : 3 out of 7 done. AM iter = 73

cv : 4 out of 7 done. AM iter = 74

cv : 5 out of 7 done. AM iter = 42

cv : 6 out of 7 done. AM iter = 40

cv : 7 out of 7 done. AM iter = 351

model id done, even death, mu = lambda, lambda* = 1. AM iter = 81

DMSO BOOT iter 74 done out of 120

cv : 1 out of 7 done. AM iter = 62

cv : 2 out of 7 done. AM iter = 62

cv : 3 out of 7 done. AM iter = 72

cv : 4 out of 7 done. AM iter = 68

cv : 5 out of 7 done. AM iter = 232

cv : 6 out of 7 done. AM iter = 75

cv : 7 out of 7 done. AM iter = 38

model id done, even death, mu = lambda, lambda* = 0.005. AM iter = 206

DMSO BOOT iter 75 done out of 120

cv : 1 out of 7 done. AM iter = 60

cv : 2 out of 7 done. AM iter = 65

cv : 3 out of 7 done. AM iter = 352

cv : 4 out of 7 done. AM iter = 1000

cv : 5 out of 7 done. AM iter = 85

cv : 6 out of 7 done. AM iter = 70

cv : 7 out of 7 done. AM iter = 76

model id done, even death, mu = lambda, lambda* = 0.01. AM iter = 72

DMSO BOOT iter 76 done out of 120

cv : 1 out of 7 done. AM iter = 709

cv : 2 out of 7 done. AM iter = 660

cv : 3 out of 7 done. AM iter = 649

cv : 4 out of 7 done. AM iter = 293

cv : 5 out of 7 done. AM iter = 347

cv : 6 out of 7 done. AM iter = 304

cv : 7 out of 7 done. AM iter = 69

model id done, even death, mu = lambda, lambda* = 0.001. AM iter = 74

DMSO BOOT iter 77 done out of 120

cv : 1 out of 7 done. AM iter = 54

cv : 2 out of 7 done. AM iter = 54

cv : 3 out of 7 done. AM iter = 54

cv : 4 out of 7 done. AM iter = 456

cv : 5 out of 7 done. AM iter = 98

cv : 6 out of 7 done. AM iter = 87

cv : 7 out of 7 done. AM iter = 41

model id done, even death, mu = lambda, lambda* = 0.005. AM iter = 136

DMSO BOOT iter 78 done out of 120

cv : 1 out of 7 done. AM iter = 63

cv : 2 out of 7 done. AM iter = 64

cv : 3 out of 7 done. AM iter = 104

cv : 4 out of 7 done. AM iter = 100

cv : 5 out of 7 done. AM iter = 78

cv : 6 out of 7 done. AM iter = 68

cv : 7 out of 7 done. AM iter = 53

model id done, even death, mu = lambda, lambda* = 0.01. AM iter = 201

DMSO BOOT iter 79 done out of 120

cv : 1 out of 7 done. AM iter = 75

cv : 2 out of 7 done. AM iter = 75

cv : 3 out of 7 done. AM iter = 109

cv : 4 out of 7 done. AM iter = 81

cv : 5 out of 7 done. AM iter = 138

cv : 6 out of 7 done. AM iter = 127

cv : 7 out of 7 done. AM iter = 46

model id done, even death, mu = lambda, lambda* = 0.01. AM iter = 197

DMSO BOOT iter 80 done out of 120

cv : 1 out of 7 done. AM iter = 71

cv : 2 out of 7 done. AM iter = 69

cv : 3 out of 7 done. AM iter = 97

cv : 4 out of 7 done. AM iter = 87

cv : 5 out of 7 done. AM iter = 145

cv : 6 out of 7 done. AM iter = 930

cv : 7 out of 7 done. AM iter = 59

model id done, even death, mu = lambda, lambda* = 0.5. AM iter = 70

DMSO BOOT iter 81 done out of 120

cv : 1 out of 7 done. AM iter = 102

cv : 2 out of 7 done. AM iter = 100

cv : 3 out of 7 done. AM iter = 83

cv : 4 out of 7 done. AM iter = 76

cv : 5 out of 7 done. AM iter = 176

cv : 6 out of 7 done. AM iter = 171

cv : 7 out of 7 done. AM iter = 56

model id done, even death, mu = lambda, lambda* = 1. AM iter = 78

DMSO BOOT iter 82 done out of 120

cv : 1 out of 7 done. AM iter = 97

cv : 2 out of 7 done. AM iter = 97

cv : 3 out of 7 done. AM iter = 121

cv : 4 out of 7 done. AM iter = 107

cv : 5 out of 7 done. AM iter = 84

cv : 6 out of 7 done. AM iter = 86

cv : 7 out of 7 done. AM iter = 43

model id done, even death, mu = lambda, lambda* = 0.005. AM iter = 171

DMSO BOOT iter 83 done out of 120

cv : 1 out of 7 done. AM iter = 236

cv : 2 out of 7 done. AM iter = 239

cv : 3 out of 7 done. AM iter = 402

cv : 4 out of 7 done. AM iter = 537

cv : 5 out of 7 done. AM iter = 137

cv : 6 out of 7 done. AM iter = 67

cv : 7 out of 7 done. AM iter = 34

model id done, even death, mu = lambda, lambda* = 0.005. AM iter = 113

DMSO BOOT iter 84 done out of 120

cv : 1 out of 7 done. AM iter = 99

cv : 2 out of 7 done. AM iter = 99

cv : 3 out of 7 done. AM iter = 95

cv : 4 out of 7 done. AM iter = 113

cv : 5 out of 7 done. AM iter = 68

cv : 6 out of 7 done. AM iter = 49

cv : 7 out of 7 done. AM iter = 188

model id done, even death, mu = lambda, lambda* = 0.001. AM iter = 95

DMSO BOOT iter 85 done out of 120

cv : 1 out of 7 done. AM iter = 66

cv : 2 out of 7 done. AM iter = 64

cv : 3 out of 7 done. AM iter = 127

cv : 4 out of 7 done. AM iter = 120

cv : 5 out of 7 done. AM iter = 729

cv : 6 out of 7 done. AM iter = 700

cv : 7 out of 7 done. AM iter = 61

model id done, even death, mu = lambda, lambda* = 0.01. AM iter = 120

DMSO BOOT iter 86 done out of 120

cv : 1 out of 7 done. AM iter = 143

cv : 2 out of 7 done. AM iter = 154

cv : 3 out of 7 done. AM iter = 72

cv : 4 out of 7 done. AM iter = 88

cv : 5 out of 7 done. AM iter = 2

cv : 6 out of 7 done. AM iter = 2

cv : 7 out of 7 done. AM iter = 83

model id done, even death, mu = lambda, lambda* = 0.01. AM iter = 509

DMSO BOOT iter 87 done out of 120

cv : 1 out of 7 done. AM iter = 87

cv : 2 out of 7 done. AM iter = 87

cv : 3 out of 7 done. AM iter = 87

cv : 4 out of 7 done. AM iter = 88

cv : 5 out of 7 done. AM iter = 195

cv : 6 out of 7 done. AM iter = 129

cv : 7 out of 7 done. AM iter = 55

model id done, even death, mu = lambda, lambda* = 0.005. AM iter = 320

DMSO BOOT iter 88 done out of 120

cv : 1 out of 7 done. AM iter = 98

cv : 2 out of 7 done. AM iter = 97

cv : 3 out of 7 done. AM iter = 151

cv : 4 out of 7 done. AM iter = 105

cv : 5 out of 7 done. AM iter = 188

cv : 6 out of 7 done. AM iter = 215

cv : 7 out of 7 done. AM iter = 82

model id done, even death, mu = lambda, lambda* = 0.05. AM iter = 193

DMSO BOOT iter 89 done out of 120

cv : 1 out of 7 done. AM iter = 44

cv : 2 out of 7 done. AM iter = 44

cv : 3 out of 7 done. AM iter = 46

cv : 4 out of 7 done. AM iter = 46

cv : 5 out of 7 done. AM iter = 106

cv : 6 out of 7 done. AM iter = 46

cv : 7 out of 7 done. AM iter = 27

model id done, even death, mu = lambda, lambda* = 0.005. AM iter = 63

DMSO BOOT iter 90 done out of 120

cv : 1 out of 7 done. AM iter = 74

cv : 2 out of 7 done. AM iter = 74

cv : 3 out of 7 done. AM iter = 75

cv : 4 out of 7 done. AM iter = 80

cv : 5 out of 7 done. AM iter = 128

cv : 6 out of 7 done. AM iter = 119

cv : 7 out of 7 done. AM iter = 49

model id done, even death, mu = lambda, lambda* = 0.01. AM iter = 146

DMSO BOOT iter 91 done out of 120

cv : 1 out of 7 done. AM iter = 58

cv : 2 out of 7 done. AM iter = 58

cv : 3 out of 7 done. AM iter = 72

cv : 4 out of 7 done. AM iter = 58

cv : 5 out of 7 done. AM iter = 86

cv : 6 out of 7 done. AM iter = 818

cv : 7 out of 7 done. AM iter = 68

model id done, even death, mu = lambda, lambda* = 0.01. AM iter = 149

DMSO BOOT iter 92 done out of 120

cv : 1 out of 7 done. AM iter = 55

cv : 2 out of 7 done. AM iter = 54

cv : 3 out of 7 done. AM iter = 62

cv : 4 out of 7 done. AM iter = 56

cv : 5 out of 7 done. AM iter = 141

cv : 6 out of 7 done. AM iter = 105

cv : 7 out of 7 done. AM iter = 38

model id done, even death, mu = lambda, lambda* = 0.05. AM iter = 89

DMSO BOOT iter 93 done out of 120

cv : 1 out of 7 done. AM iter = 87

cv : 2 out of 7 done. AM iter = 87

cv : 3 out of 7 done. AM iter = 86

cv : 4 out of 7 done. AM iter = 104

cv : 5 out of 7 done. AM iter = 93

cv : 6 out of 7 done. AM iter = 56

cv : 7 out of 7 done. AM iter = 84

model id done, even death, mu = lambda, lambda* = 0.01. AM iter = 49

DMSO BOOT iter 94 done out of 120

cv : 1 out of 7 done. AM iter = 84

cv : 2 out of 7 done. AM iter = 84

cv : 3 out of 7 done. AM iter = 84

cv : 4 out of 7 done. AM iter = 92

cv : 5 out of 7 done. AM iter = 30

cv : 6 out of 7 done. AM iter = 25

cv : 7 out of 7 done. AM iter = 58

model id done, even death, mu = lambda, lambda* = 0.01. AM iter = 41

DMSO BOOT iter 95 done out of 120

cv : 1 out of 7 done. AM iter = 52

cv : 2 out of 7 done. AM iter = 52

cv : 3 out of 7 done. AM iter = 52

cv : 4 out of 7 done. AM iter = 52

cv : 5 out of 7 done. AM iter = 53

cv : 6 out of 7 done. AM iter = 191

cv : 7 out of 7 done. AM iter = 45

model id done, even death, mu = lambda, lambda* = 0.005. AM iter = 269

DMSO BOOT iter 96 done out of 120

cv : 1 out of 7 done. AM iter = 100

cv : 2 out of 7 done. AM iter = 101

cv : 3 out of 7 done. AM iter = 83

cv : 4 out of 7 done. AM iter = 110

cv : 5 out of 7 done. AM iter = 51

cv : 6 out of 7 done. AM iter = 44

cv : 7 out of 7 done. AM iter = 229

model id done, even death, mu = lambda, lambda* = 0.005. AM iter = 756

DMSO BOOT iter 97 done out of 120

cv : 1 out of 7 done. AM iter = 60

cv : 2 out of 7 done. AM iter = 113

cv : 3 out of 7 done. AM iter = 76

cv : 4 out of 7 done. AM iter = 72

cv : 5 out of 7 done. AM iter = 61

cv : 6 out of 7 done. AM iter = 57

cv : 7 out of 7 done. AM iter = 32

model id done, even death, mu = lambda, lambda* = 0.005. AM iter = 74

DMSO BOOT iter 98 done out of 120

cv : 1 out of 7 done. AM iter = 92

cv : 2 out of 7 done. AM iter = 91

cv : 3 out of 7 done. AM iter = 86

cv : 4 out of 7 done. AM iter = 90

cv : 5 out of 7 done. AM iter = 129

cv : 6 out of 7 done. AM iter = 189

cv : 7 out of 7 done. AM iter = 83

model id done, even death, mu = lambda, lambda* = 0.005. AM iter = 203

DMSO BOOT iter 99 done out of 120

cv : 1 out of 7 done. AM iter = 361

cv : 2 out of 7 done. AM iter = 353

cv : 3 out of 7 done. AM iter = 194

cv : 4 out of 7 done. AM iter = 112

cv : 5 out of 7 done. AM iter = 150

cv : 6 out of 7 done. AM iter = 99

cv : 7 out of 7 done. AM iter = 102

model id done, even death, mu = lambda, lambda* = 0.05. AM iter = 79

DMSO BOOT iter 100 done out of 120

cv : 1 out of 7 done. AM iter = 69

cv : 2 out of 7 done. AM iter = 68

cv : 3 out of 7 done. AM iter = 133

cv : 4 out of 7 done. AM iter = 108

cv : 5 out of 7 done. AM iter = 169

cv : 6 out of 7 done. AM iter = 178

cv : 7 out of 7 done. AM iter = 80

model id done, even death, mu = lambda, lambda* = 0.005. AM iter = 187

DMSO BOOT iter 101 done out of 120

cv : 1 out of 7 done. AM iter = 53

cv : 2 out of 7 done. AM iter = 53

cv : 3 out of 7 done. AM iter = 53

cv : 4 out of 7 done. AM iter = 53

cv : 5 out of 7 done. AM iter = 61

cv : 6 out of 7 done. AM iter = 58

cv : 7 out of 7 done. AM iter = 148

model id done, even death, mu = lambda, lambda* = 0.001. AM iter = 84

DMSO BOOT iter 102 done out of 120

cv : 1 out of 7 done. AM iter = 133

cv : 2 out of 7 done. AM iter = 133

cv : 3 out of 7 done. AM iter = 288

cv : 4 out of 7 done. AM iter = 559

cv : 5 out of 7 done. AM iter = 65

cv : 6 out of 7 done. AM iter = 54

cv : 7 out of 7 done. AM iter = 296

model id done, even death, mu = lambda, lambda* = 0.001. AM iter = 125

DMSO BOOT iter 103 done out of 120

cv : 1 out of 7 done. AM iter = 153

cv : 2 out of 7 done. AM iter = 155

cv : 3 out of 7 done. AM iter = 133

cv : 4 out of 7 done. AM iter = 109

cv : 5 out of 7 done. AM iter = 112

cv : 6 out of 7 done. AM iter = 95

cv : 7 out of 7 done. AM iter = 83

model id done, even death, mu = lambda, lambda* = 0.01. AM iter = 50

DMSO BOOT iter 104 done out of 120

cv : 1 out of 7 done. AM iter = 34

cv : 2 out of 7 done. AM iter = 35

cv : 3 out of 7 done. AM iter = 29

cv : 4 out of 7 done. AM iter = 32

cv : 5 out of 7 done. AM iter = 100

cv : 6 out of 7 done. AM iter = 71

cv : 7 out of 7 done. AM iter = 126

model id done, even death, mu = lambda, lambda* = 0.01. AM iter = 277

DMSO BOOT iter 105 done out of 120

cv : 1 out of 7 done. AM iter = 81

cv : 2 out of 7 done. AM iter = 81

cv : 3 out of 7 done. AM iter = 97

cv : 4 out of 7 done. AM iter = 91

cv : 5 out of 7 done. AM iter = 46

cv : 6 out of 7 done. AM iter = 53

cv : 7 out of 7 done. AM iter = 158

model id done, even death, mu = lambda, lambda* = 0.01. AM iter = 53

DMSO BOOT iter 106 done out of 120

cv : 1 out of 7 done. AM iter = 93

cv : 2 out of 7 done. AM iter = 92

cv : 3 out of 7 done. AM iter = 91

cv : 4 out of 7 done. AM iter = 130

cv : 5 out of 7 done. AM iter = 246

cv : 6 out of 7 done. AM iter = 312

cv : 7 out of 7 done. AM iter = 49

model id done, even death, mu = lambda, lambda* = 0.005. AM iter = 1000

DMSO BOOT iter 107 done out of 120

cv : 1 out of 7 done. AM iter = 83

cv : 2 out of 7 done. AM iter = 83

cv : 3 out of 7 done. AM iter = 89

cv : 4 out of 7 done. AM iter = 71

cv : 5 out of 7 done. AM iter = 1000

cv : 6 out of 7 done. AM iter = 1000

cv : 7 out of 7 done. AM iter = 84

model id done, even death, mu = lambda, lambda* = 0.005. AM iter = 204

DMSO BOOT iter 108 done out of 120

cv : 1 out of 7 done. AM iter = 89

cv : 2 out of 7 done. AM iter = 89

cv : 3 out of 7 done. AM iter = 95

cv : 4 out of 7 done. AM iter = 102

cv : 5 out of 7 done. AM iter = 312

cv : 6 out of 7 done. AM iter = 90

cv : 7 out of 7 done. AM iter = 32

model id done, even death, mu = lambda, lambda* = 0.01. AM iter = 123

DMSO BOOT iter 109 done out of 120

cv : 1 out of 7 done. AM iter = 114

cv : 2 out of 7 done. AM iter = 114

cv : 3 out of 7 done. AM iter = 114

cv : 4 out of 7 done. AM iter = 116

cv : 5 out of 7 done. AM iter = 82

cv : 6 out of 7 done. AM iter = 82

cv : 7 out of 7 done. AM iter = 346

model id done, even death, mu = lambda, lambda* = 0.005. AM iter = 67

DMSO BOOT iter 110 done out of 120

cv : 1 out of 7 done. AM iter = 140

cv : 2 out of 7 done. AM iter = 140

cv : 3 out of 7 done. AM iter = 99

cv : 4 out of 7 done. AM iter = 100

cv : 5 out of 7 done. AM iter = 638

cv : 6 out of 7 done. AM iter = 743

cv : 7 out of 7 done. AM iter = 50

model id done, even death, mu = lambda, lambda* = 0.001. AM iter = 48

DMSO BOOT iter 111 done out of 120

cv : 1 out of 7 done. AM iter = 77

cv : 2 out of 7 done. AM iter = 75

cv : 3 out of 7 done. AM iter = 64

cv : 4 out of 7 done. AM iter = 81

cv : 5 out of 7 done. AM iter = 154

cv : 6 out of 7 done. AM iter = 134

cv : 7 out of 7 done. AM iter = 45

model id done, even death, mu = lambda, lambda* = 0.05. AM iter = 92

DMSO BOOT iter 112 done out of 120

cv : 1 out of 7 done. AM iter = 85

cv : 2 out of 7 done. AM iter = 85

cv : 3 out of 7 done. AM iter = 87

cv : 4 out of 7 done. AM iter = 117

cv : 5 out of 7 done. AM iter = 137

cv : 6 out of 7 done. AM iter = 116

cv : 7 out of 7 done. AM iter = 117

model id done, even death, mu = lambda, lambda* = 0.001. AM iter = 113

DMSO BOOT iter 113 done out of 120

cv : 1 out of 7 done. AM iter = 117

cv : 2 out of 7 done. AM iter = 112

cv : 3 out of 7 done. AM iter = 92

cv : 4 out of 7 done. AM iter = 89

cv : 5 out of 7 done. AM iter = 142

cv : 6 out of 7 done. AM iter = 100

cv : 7 out of 7 done. AM iter = 40

model id done, even death, mu = lambda, lambda* = 0.005. AM iter = 266

DMSO BOOT iter 114 done out of 120

cv : 1 out of 7 done. AM iter = 77

cv : 2 out of 7 done. AM iter = 77

cv : 3 out of 7 done. AM iter = 77

cv : 4 out of 7 done. AM iter = 77

cv : 5 out of 7 done. AM iter = 238

cv : 6 out of 7 done. AM iter = 296

cv : 7 out of 7 done. AM iter = 115

model id done, even death, mu = lambda, lambda* = 0.005. AM iter = 414

DMSO BOOT iter 115 done out of 120

cv : 1 out of 7 done. AM iter = 93

cv : 2 out of 7 done. AM iter = 92

cv : 3 out of 7 done. AM iter = 90

cv : 4 out of 7 done. AM iter = 93

cv : 5 out of 7 done. AM iter = 103

cv : 6 out of 7 done. AM iter = 78

cv : 7 out of 7 done. AM iter = 38

model id done, even death, mu = lambda, lambda* = 0.001. AM iter = 45

DMSO BOOT iter 116 done out of 120

cv : 1 out of 7 done. AM iter = 284

cv : 2 out of 7 done. AM iter = 284

cv : 3 out of 7 done. AM iter = 151

cv : 4 out of 7 done. AM iter = 154

cv : 5 out of 7 done. AM iter = 76

cv : 6 out of 7 done. AM iter = 68

cv : 7 out of 7 done. AM iter = 149

model id done, even death, mu = lambda, lambda* = 0.01. AM iter = 480

DMSO BOOT iter 117 done out of 120

cv : 1 out of 7 done. AM iter = 113

cv : 2 out of 7 done. AM iter = 113

cv : 3 out of 7 done. AM iter = 1000

cv : 4 out of 7 done. AM iter = 250

cv : 5 out of 7 done. AM iter = 43

cv : 6 out of 7 done. AM iter = 43

cv : 7 out of 7 done. AM iter = 191

model id done, even death, mu = lambda, lambda* = 0.01. AM iter = 37

DMSO BOOT iter 118 done out of 120

cv : 1 out of 7 done. AM iter = 68

cv : 2 out of 7 done. AM iter = 68

cv : 3 out of 7 done. AM iter = 74

cv : 4 out of 7 done. AM iter = 125

cv : 5 out of 7 done. AM iter = 41

cv : 6 out of 7 done. AM iter = 36

cv : 7 out of 7 done. AM iter = 180

model id done, even death, mu = lambda, lambda* = 0.01. AM iter = 54

DMSO BOOT iter 119 done out of 120

cv : 1 out of 7 done. AM iter = 161

cv : 2 out of 7 done. AM iter = 161

cv : 3 out of 7 done. AM iter = 140

cv : 4 out of 7 done. AM iter = 76

cv : 5 out of 7 done. AM iter = 83

cv : 6 out of 7 done. AM iter = 90

cv : 7 out of 7 done. AM iter = 68

model id done, even death, mu = lambda, lambda* = 0.01. AM iter = 396

DMSO BOOT iter 120 done out of 120

cv : 1 out of 7 done. AM iter = 92

cv : 2 out of 7 done. AM iter = 90

cv : 3 out of 7 done. AM iter = 126

cv : 4 out of 7 done. AM iter = 77

cv : 5 out of 7 done. AM iter = 64

cv : 6 out of 7 done. AM iter = 52

cv : 7 out of 7 done. AM iter = 10

model id done, even death, mu = lambda, lambda* = 1. AM iter = 111

Tram. BOOT iter 1 done out of 120

cv : 1 out of 7 done. AM iter = 74

cv : 2 out of 7 done. AM iter = 74

cv : 3 out of 7 done. AM iter = 82

cv : 4 out of 7 done. AM iter = 78

cv : 5 out of 7 done. AM iter = 36

cv : 6 out of 7 done. AM iter = 29

cv : 7 out of 7 done. AM iter = 10

model id done, even death, mu = lambda, lambda* = 0.005. AM iter = 64

Tram. BOOT iter 2 done out of 120

cv : 1 out of 7 done. AM iter = 77

cv : 2 out of 7 done. AM iter = 76

cv : 3 out of 7 done. AM iter = 72

cv : 4 out of 7 done. AM iter = 68

cv : 5 out of 7 done. AM iter = 79

cv : 6 out of 7 done. AM iter = 58

cv : 7 out of 7 done. AM iter = 13

model id done, even death, mu = lambda, lambda* = 0.01. AM iter = 77

Tram. BOOT iter 3 done out of 120

cv : 1 out of 7 done. AM iter = 53

cv : 2 out of 7 done. AM iter = 53

cv : 3 out of 7 done. AM iter = 53

cv : 4 out of 7 done. AM iter = 69

cv : 5 out of 7 done. AM iter = 78

cv : 6 out of 7 done. AM iter = 64

cv : 7 out of 7 done. AM iter = 12

model id done, even death, mu = lambda, lambda* = 0.05. AM iter = 86

Tram. BOOT iter 4 done out of 120

cv : 1 out of 7 done. AM iter = 105

cv : 2 out of 7 done. AM iter = 101

cv : 3 out of 7 done. AM iter = 80

cv : 4 out of 7 done. AM iter = 106

cv : 5 out of 7 done. AM iter = 61

cv : 6 out of 7 done. AM iter = 49

cv : 7 out of 7 done. AM iter = 12

model id done, even death, mu = lambda, lambda* = 0.005. AM iter = 63

Tram. BOOT iter 5 done out of 120

cv : 1 out of 7 done. AM iter = 135

cv : 2 out of 7 done. AM iter = 133

cv : 3 out of 7 done. AM iter = 135

cv : 4 out of 7 done. AM iter = 210

cv : 5 out of 7 done. AM iter = 59

cv : 6 out of 7 done. AM iter = 42

cv : 7 out of 7 done. AM iter = 11

model id done, even death, mu = lambda, lambda* = 0.05. AM iter = 74

Tram. BOOT iter 6 done out of 120

cv : 1 out of 7 done. AM iter = 215

cv : 2 out of 7 done. AM iter = 204

cv : 3 out of 7 done. AM iter = 152

cv : 4 out of 7 done. AM iter = 216

cv : 5 out of 7 done. AM iter = 90

cv : 6 out of 7 done. AM iter = 77

cv : 7 out of 7 done. AM iter = 14

model id done, even death, mu = lambda, lambda* = 0.05. AM iter = 238

Tram. BOOT iter 7 done out of 120

cv : 1 out of 7 done. AM iter = 77

cv : 2 out of 7 done. AM iter = 77

cv : 3 out of 7 done. AM iter = 90

cv : 4 out of 7 done. AM iter = 95

cv : 5 out of 7 done. AM iter = 67

cv : 6 out of 7 done. AM iter = 50

cv : 7 out of 7 done. AM iter = 10

model id done, even death, mu = lambda, lambda* = 0.05. AM iter = 110

Tram. BOOT iter 8 done out of 120

cv : 1 out of 7 done. AM iter = 92

cv : 2 out of 7 done. AM iter = 90

cv : 3 out of 7 done. AM iter = 75

cv : 4 out of 7 done. AM iter = 111

cv : 5 out of 7 done. AM iter = 64

cv : 6 out of 7 done. AM iter = 47

cv : 7 out of 7 done. AM iter = 12

model id done, even death, mu = lambda, lambda* = 0.01. AM iter = 68

Tram. BOOT iter 9 done out of 120

cv : 1 out of 7 done. AM iter = 84

cv : 2 out of 7 done. AM iter = 84

cv : 3 out of 7 done. AM iter = 118

cv : 4 out of 7 done. AM iter = 107

cv : 5 out of 7 done. AM iter = 49

cv : 6 out of 7 done. AM iter = 41

cv : 7 out of 7 done. AM iter = 10

model id done, even death, mu = lambda, lambda* = 0.005. AM iter = 107

Tram. BOOT iter 10 done out of 120

cv : 1 out of 7 done. AM iter = 43

cv : 2 out of 7 done. AM iter = 43

cv : 3 out of 7 done. AM iter = 47

cv : 4 out of 7 done. AM iter = 86

cv : 5 out of 7 done. AM iter = 51

cv : 6 out of 7 done. AM iter = 40

cv : 7 out of 7 done. AM iter = 10

model id done, even death, mu = lambda, lambda* = 1. AM iter = 52

Tram. BOOT iter 11 done out of 120

cv : 1 out of 7 done. AM iter = 321

cv : 2 out of 7 done. AM iter = 307

cv : 3 out of 7 done. AM iter = 71

cv : 4 out of 7 done. AM iter = 70

cv : 5 out of 7 done. AM iter = 45

cv : 6 out of 7 done. AM iter = 35

cv : 7 out of 7 done. AM iter = 10

model id done, even death, mu = lambda, lambda* = 0.005. AM iter = 31

Tram. BOOT iter 12 done out of 120

cv : 1 out of 7 done. AM iter = 79

cv : 2 out of 7 done. AM iter = 79

cv : 3 out of 7 done. AM iter = 322

cv : 4 out of 7 done. AM iter = 132

cv : 5 out of 7 done. AM iter = 57

cv : 6 out of 7 done. AM iter = 45

cv : 7 out of 7 done. AM iter = 10

model id done, even death, mu = lambda, lambda* = 0.005. AM iter = 70

Tram. BOOT iter 13 done out of 120

cv : 1 out of 7 done. AM iter = 98

cv : 2 out of 7 done. AM iter = 98

cv : 3 out of 7 done. AM iter = 121

cv : 4 out of 7 done. AM iter = 112

cv : 5 out of 7 done. AM iter = 87

cv : 6 out of 7 done. AM iter = 71

cv : 7 out of 7 done. AM iter = 14

model id done, even death, mu = lambda, lambda* = 0.1. AM iter = 80

Tram. BOOT iter 14 done out of 120

cv : 1 out of 7 done. AM iter = 48

cv : 2 out of 7 done. AM iter = 48

cv : 3 out of 7 done. AM iter = 74

cv : 4 out of 7 done. AM iter = 54

cv : 5 out of 7 done. AM iter = 51

cv : 6 out of 7 done. AM iter = 39

cv : 7 out of 7 done. AM iter = 10

model id done, even death, mu = lambda, lambda* = 0.05. AM iter = 70

Tram. BOOT iter 15 done out of 120

cv : 1 out of 7 done. AM iter = 43

cv : 2 out of 7 done. AM iter = 45

cv : 3 out of 7 done. AM iter = 106

cv : 4 out of 7 done. AM iter = 138

cv : 5 out of 7 done. AM iter = 46

cv : 6 out of 7 done. AM iter = 1000

cv : 7 out of 7 done. AM iter = 11

model id done, even death, mu = lambda, lambda* = 0.05. AM iter = 127

Tram. BOOT iter 16 done out of 120

cv : 1 out of 7 done. AM iter = 65

cv : 2 out of 7 done. AM iter = 87

cv : 3 out of 7 done. AM iter = 71

cv : 4 out of 7 done. AM iter = 80

cv : 5 out of 7 done. AM iter = 42

cv : 6 out of 7 done. AM iter = 30

cv : 7 out of 7 done. AM iter = 11

model id done, even death, mu = lambda, lambda* = 0.01. AM iter = 51

Tram. BOOT iter 17 done out of 120

cv : 1 out of 7 done. AM iter = 642

cv : 2 out of 7 done. AM iter = 440

cv : 3 out of 7 done. AM iter = 280

cv : 4 out of 7 done. AM iter = 142

cv : 5 out of 7 done. AM iter = 57

cv : 6 out of 7 done. AM iter = 42

cv : 7 out of 7 done. AM iter = 13

model id done, even death, mu = lambda, lambda* = 0.005. AM iter = 57

Tram. BOOT iter 18 done out of 120

cv : 1 out of 7 done. AM iter = 68

cv : 2 out of 7 done. AM iter = 68

cv : 3 out of 7 done. AM iter = 94

cv : 4 out of 7 done. AM iter = 124

cv : 5 out of 7 done. AM iter = 86

cv : 6 out of 7 done. AM iter = 70

cv : 7 out of 7 done. AM iter = 13

model id done, even death, mu = lambda, lambda* = 0.5. AM iter = 59

Tram. BOOT iter 19 done out of 120

cv : 1 out of 7 done. AM iter = 117

cv : 2 out of 7 done. AM iter = 142

cv : 3 out of 7 done. AM iter = 101

cv : 4 out of 7 done. AM iter = 115

cv : 5 out of 7 done. AM iter = 58

cv : 6 out of 7 done. AM iter = 45

cv : 7 out of 7 done. AM iter = 13

model id done, even death, mu = lambda, lambda* = 0.05. AM iter = 72

Tram. BOOT iter 20 done out of 120

cv : 1 out of 7 done. AM iter = 63

cv : 2 out of 7 done. AM iter = 62

cv : 3 out of 7 done. AM iter = 61

cv : 4 out of 7 done. AM iter = 239

cv : 5 out of 7 done. AM iter = 102

cv : 6 out of 7 done. AM iter = 89

cv : 7 out of 7 done. AM iter = 13

model id done, even death, mu = lambda, lambda* = 0.1. AM iter = 253

Tram. BOOT iter 21 done out of 120

cv : 1 out of 7 done. AM iter = 134

cv : 2 out of 7 done. AM iter = 128

cv : 3 out of 7 done. AM iter = 125

cv : 4 out of 7 done. AM iter = 88

cv : 5 out of 7 done. AM iter = 47

cv : 6 out of 7 done. AM iter = 33

cv : 7 out of 7 done. AM iter = 12

model id done, even death, mu = lambda, lambda* = 1. AM iter = 90

Tram. BOOT iter 22 done out of 120

cv : 1 out of 7 done. AM iter = 142

cv : 2 out of 7 done. AM iter = 137

cv : 3 out of 7 done. AM iter = 109

cv : 4 out of 7 done. AM iter = 200

cv : 5 out of 7 done. AM iter = 117

cv : 6 out of 7 done. AM iter = 83

cv : 7 out of 7 done. AM iter = 13

model id done, even death, mu = lambda, lambda* = 0.01. AM iter = 125

Tram. BOOT iter 23 done out of 120

cv : 1 out of 7 done. AM iter = 54

cv : 2 out of 7 done. AM iter = 51

cv : 3 out of 7 done. AM iter = 243

cv : 4 out of 7 done. AM iter = 114

cv : 5 out of 7 done. AM iter = 35

cv : 6 out of 7 done. AM iter = 29

cv : 7 out of 7 done. AM iter = 12

model id done, even death, mu = lambda, lambda* = 0.5. AM iter = 57

Tram. BOOT iter 24 done out of 120

cv : 1 out of 7 done. AM iter = 95

cv : 2 out of 7 done. AM iter = 93

cv : 3 out of 7 done. AM iter = 87

cv : 4 out of 7 done. AM iter = 93

cv : 5 out of 7 done. AM iter = 48

cv : 6 out of 7 done. AM iter = 39

cv : 7 out of 7 done. AM iter = 11

model id done, even death, mu = lambda, lambda* = 0.1. AM iter = 71

Tram. BOOT iter 25 done out of 120

cv : 1 out of 7 done. AM iter = 45

cv : 2 out of 7 done. AM iter = 44

cv : 3 out of 7 done. AM iter = 43

cv : 4 out of 7 done. AM iter = 124

cv : 5 out of 7 done. AM iter = 54

cv : 6 out of 7 done. AM iter = 43

cv : 7 out of 7 done. AM iter = 14

model id done, even death, mu = lambda, lambda* = 0.01. AM iter = 84

Tram. BOOT iter 26 done out of 120

cv : 1 out of 7 done. AM iter = 90

cv : 2 out of 7 done. AM iter = 94

cv : 3 out of 7 done. AM iter = 86

cv : 4 out of 7 done. AM iter = 74

cv : 5 out of 7 done. AM iter = 43

cv : 6 out of 7 done. AM iter = 35

cv : 7 out of 7 done. AM iter = 12

model id done, even death, mu = lambda, lambda* = 0.5. AM iter = 83

Tram. BOOT iter 27 done out of 120

cv : 1 out of 7 done. AM iter = 96

cv : 2 out of 7 done. AM iter = 122

cv : 3 out of 7 done. AM iter = 141

cv : 4 out of 7 done. AM iter = 168

cv : 5 out of 7 done. AM iter = 73

cv : 6 out of 7 done. AM iter = 52

cv : 7 out of 7 done. AM iter = 11

model id done, even death, mu = lambda, lambda* = 0.1. AM iter = 218

Tram. BOOT iter 28 done out of 120

cv : 1 out of 7 done. AM iter = 128

cv : 2 out of 7 done. AM iter = 126

cv : 3 out of 7 done. AM iter = 118

cv : 4 out of 7 done. AM iter = 110

cv : 5 out of 7 done. AM iter = 44

cv : 6 out of 7 done. AM iter = 33

cv : 7 out of 7 done. AM iter = 14

model id done, even death, mu = lambda, lambda* = 0.01. AM iter = 65

Tram. BOOT iter 29 done out of 120

cv : 1 out of 7 done. AM iter = 89

cv : 2 out of 7 done. AM iter = 98

cv : 3 out of 7 done. AM iter = 132

cv : 4 out of 7 done. AM iter = 121

cv : 5 out of 7 done. AM iter = 80

cv : 6 out of 7 done. AM iter = 71

cv : 7 out of 7 done. AM iter = 15

model id done, even death, mu = lambda, lambda* = 0.1. AM iter = 86

Tram. BOOT iter 30 done out of 120

cv : 1 out of 7 done. AM iter = 58

cv : 2 out of 7 done. AM iter = 57

cv : 3 out of 7 done. AM iter = 63

cv : 4 out of 7 done. AM iter = 158

cv : 5 out of 7 done. AM iter = 48

cv : 6 out of 7 done. AM iter = 30

cv : 7 out of 7 done. AM iter = 10

model id done, even death, mu = lambda, lambda* = 0.01. AM iter = 87

Tram. BOOT iter 31 done out of 120

cv : 1 out of 7 done. AM iter = 184

cv : 2 out of 7 done. AM iter = 187

cv : 3 out of 7 done. AM iter = 313

cv : 4 out of 7 done. AM iter = 311

cv : 5 out of 7 done. AM iter = 231

cv : 6 out of 7 done. AM iter = 166

cv : 7 out of 7 done. AM iter = 14

model id done, even death, mu = lambda, lambda* = 1. AM iter = 318

Tram. BOOT iter 32 done out of 120

cv : 1 out of 7 done. AM iter = 70

cv : 2 out of 7 done. AM iter = 68

cv : 3 out of 7 done. AM iter = 93

cv : 4 out of 7 done. AM iter = 86

cv : 5 out of 7 done. AM iter = 44

cv : 6 out of 7 done. AM iter = 39

cv : 7 out of 7 done. AM iter = 11

model id done, even death, mu = lambda, lambda* = 0.1. AM iter = 78

Tram. BOOT iter 33 done out of 120

cv : 1 out of 7 done. AM iter = 49

cv : 2 out of 7 done. AM iter = 50

cv : 3 out of 7 done. AM iter = 90

cv : 4 out of 7 done. AM iter = 95

cv : 5 out of 7 done. AM iter = 41

cv : 6 out of 7 done. AM iter = 31

cv : 7 out of 7 done. AM iter = 11

model id done, even death, mu = lambda, lambda* = 0.01. AM iter = 55

Tram. BOOT iter 34 done out of 120

cv : 1 out of 7 done. AM iter = 119

cv : 2 out of 7 done. AM iter = 119

cv : 3 out of 7 done. AM iter = 392

cv : 4 out of 7 done. AM iter = 170

cv : 5 out of 7 done. AM iter = 67

cv : 6 out of 7 done. AM iter = 48

cv : 7 out of 7 done. AM iter = 10

model id done, even death, mu = lambda, lambda* = 0.01. AM iter = 40

Tram. BOOT iter 35 done out of 120

cv : 1 out of 7 done. AM iter = 70

cv : 2 out of 7 done. AM iter = 68

cv : 3 out of 7 done. AM iter = 65

cv : 4 out of 7 done. AM iter = 90

cv : 5 out of 7 done. AM iter = 50

cv : 6 out of 7 done. AM iter = 48

cv : 7 out of 7 done. AM iter = 12

model id done, even death, mu = lambda, lambda* = 0.01. AM iter = 54

Tram. BOOT iter 36 done out of 120

cv : 1 out of 7 done. AM iter = 44

cv : 2 out of 7 done. AM iter = 44

cv : 3 out of 7 done. AM iter = 43

cv : 4 out of 7 done. AM iter = 135

cv : 5 out of 7 done. AM iter = 47

cv : 6 out of 7 done. AM iter = 37

cv : 7 out of 7 done. AM iter = 10

model id done, even death, mu = lambda, lambda* = 0.01. AM iter = 60

Tram. BOOT iter 37 done out of 120

cv : 1 out of 7 done. AM iter = 177

cv : 2 out of 7 done. AM iter = 173

cv : 3 out of 7 done. AM iter = 111

cv : 4 out of 7 done. AM iter = 87

cv : 5 out of 7 done. AM iter = 62

cv : 6 out of 7 done. AM iter = 45

cv : 7 out of 7 done. AM iter = 12

model id done, even death, mu = lambda, lambda* = 0.005. AM iter = 61

Tram. BOOT iter 38 done out of 120

cv : 1 out of 7 done. AM iter = 142

cv : 2 out of 7 done. AM iter = 159

cv : 3 out of 7 done. AM iter = 293

cv : 4 out of 7 done. AM iter = 240

cv : 5 out of 7 done. AM iter = 109

cv : 6 out of 7 done. AM iter = 80

cv : 7 out of 7 done. AM iter = 15

model id done, even death, mu = lambda, lambda* = 0.01. AM iter = 152

Tram. BOOT iter 39 done out of 120

cv : 1 out of 7 done. AM iter = 53

cv : 2 out of 7 done. AM iter = 54

cv : 3 out of 7 done. AM iter = 138

cv : 4 out of 7 done. AM iter = 139

cv : 5 out of 7 done. AM iter = 56

cv : 6 out of 7 done. AM iter = 44

cv : 7 out of 7 done. AM iter = 10

model id done, even death, mu = lambda, lambda* = 0.1. AM iter = 69

Tram. BOOT iter 40 done out of 120

cv : 1 out of 7 done. AM iter = 177

cv : 2 out of 7 done. AM iter = 168

cv : 3 out of 7 done. AM iter = 132

cv : 4 out of 7 done. AM iter = 149

cv : 5 out of 7 done. AM iter = 103

cv : 6 out of 7 done. AM iter = 82

cv : 7 out of 7 done. AM iter = 14

model id done, even death, mu = lambda, lambda* = 0.1. AM iter = 124

Tram. BOOT iter 41 done out of 120

cv : 1 out of 7 done. AM iter = 167

cv : 2 out of 7 done. AM iter = 169

cv : 3 out of 7 done. AM iter = 208

cv : 4 out of 7 done. AM iter = 175

cv : 5 out of 7 done. AM iter = 65

cv : 6 out of 7 done. AM iter = 46

cv : 7 out of 7 done. AM iter = 11

model id done, even death, mu = lambda, lambda* = 0.1. AM iter = 210

Tram. BOOT iter 42 done out of 120

cv : 1 out of 7 done. AM iter = 187

cv : 2 out of 7 done. AM iter = 182

cv : 3 out of 7 done. AM iter = 249

cv : 4 out of 7 done. AM iter = 181

cv : 5 out of 7 done. AM iter = 109

cv : 6 out of 7 done. AM iter = 99

cv : 7 out of 7 done. AM iter = 12

model id done, even death, mu = lambda, lambda* = 0.05. AM iter = 180

Tram. BOOT iter 43 done out of 120

cv : 1 out of 7 done. AM iter = 127

cv : 2 out of 7 done. AM iter = 127

cv : 3 out of 7 done. AM iter = 117

cv : 4 out of 7 done. AM iter = 185

cv : 5 out of 7 done. AM iter = 94

cv : 6 out of 7 done. AM iter = 67

cv : 7 out of 7 done. AM iter = 11

model id done, even death, mu = lambda, lambda* = 0.01. AM iter = 147

Tram. BOOT iter 44 done out of 120

cv : 1 out of 7 done. AM iter = 96

cv : 2 out of 7 done. AM iter = 89

cv : 3 out of 7 done. AM iter = 74

cv : 4 out of 7 done. AM iter = 116

cv : 5 out of 7 done. AM iter = 53

cv : 6 out of 7 done. AM iter = 44

cv : 7 out of 7 done. AM iter = 12

model id done, even death, mu = lambda, lambda* = 0.05. AM iter = 98

Tram. BOOT iter 45 done out of 120

cv : 1 out of 7 done. AM iter = 96

cv : 2 out of 7 done. AM iter = 84

cv : 3 out of 7 done. AM iter = 248

cv : 4 out of 7 done. AM iter = 174

cv : 5 out of 7 done. AM iter = 97

cv : 6 out of 7 done. AM iter = 73

cv : 7 out of 7 done. AM iter = 12

model id done, even death, mu = lambda, lambda* = 0.05. AM iter = 118

Tram. BOOT iter 46 done out of 120

cv : 1 out of 7 done. AM iter = 91

cv : 2 out of 7 done. AM iter = 88

cv : 3 out of 7 done. AM iter = 70

cv : 4 out of 7 done. AM iter = 56

cv : 5 out of 7 done. AM iter = 31

cv : 6 out of 7 done. AM iter = 22

cv : 7 out of 7 done. AM iter = 12

model id done, even death, mu = lambda, lambda* = 1. AM iter = 50

Tram. BOOT iter 47 done out of 120

cv : 1 out of 7 done. AM iter = 128

cv : 2 out of 7 done. AM iter = 126

cv : 3 out of 7 done. AM iter = 149

cv : 4 out of 7 done. AM iter = 185

cv : 5 out of 7 done. AM iter = 66

cv : 6 out of 7 done. AM iter = 57

cv : 7 out of 7 done. AM iter = 13

model id done, even death, mu = lambda, lambda* = 0.1. AM iter = 82

Tram. BOOT iter 48 done out of 120

cv : 1 out of 7 done. AM iter = 82

cv : 2 out of 7 done. AM iter = 84

cv : 3 out of 7 done. AM iter = 144

cv : 4 out of 7 done. AM iter = 115

cv : 5 out of 7 done. AM iter = 107

cv : 6 out of 7 done. AM iter = 89

cv : 7 out of 7 done. AM iter = 12

model id done, even death, mu = lambda, lambda* = 0.005. AM iter = 86

Tram. BOOT iter 49 done out of 120

cv : 1 out of 7 done. AM iter = 131

cv : 2 out of 7 done. AM iter = 95

cv : 3 out of 7 done. AM iter = 169

cv : 4 out of 7 done. AM iter = 139

cv : 5 out of 7 done. AM iter = 98

cv : 6 out of 7 done. AM iter = 73

cv : 7 out of 7 done. AM iter = 11

model id done, even death, mu = lambda, lambda* = 1. AM iter = 80

Tram. BOOT iter 50 done out of 120

cv : 1 out of 7 done. AM iter = 149

cv : 2 out of 7 done. AM iter = 143

cv : 3 out of 7 done. AM iter = 144

cv : 4 out of 7 done. AM iter = 121

cv : 5 out of 7 done. AM iter = 74

cv : 6 out of 7 done. AM iter = 60

cv : 7 out of 7 done. AM iter = 12

model id done, even death, mu = lambda, lambda* = 0.1. AM iter = 1000

Tram. BOOT iter 51 done out of 120

cv : 1 out of 7 done. AM iter = 126

cv : 2 out of 7 done. AM iter = 120

cv : 3 out of 7 done. AM iter = 90

cv : 4 out of 7 done. AM iter = 88

cv : 5 out of 7 done. AM iter = 61

cv : 6 out of 7 done. AM iter = 51

cv : 7 out of 7 done. AM iter = 15

model id done, even death, mu = lambda, lambda* = 0.005. AM iter = 49

Tram. BOOT iter 52 done out of 120

cv : 1 out of 7 done. AM iter = 112

cv : 2 out of 7 done. AM iter = 108

cv : 3 out of 7 done. AM iter = 89

cv : 4 out of 7 done. AM iter = 135

cv : 5 out of 7 done. AM iter = 111

cv : 6 out of 7 done. AM iter = 94

cv : 7 out of 7 done. AM iter = 14

model id done, even death, mu = lambda, lambda* = 0.05. AM iter = 125

Tram. BOOT iter 53 done out of 120

cv : 1 out of 7 done. AM iter = 95

cv : 2 out of 7 done. AM iter = 96

cv : 3 out of 7 done. AM iter = 329

cv : 4 out of 7 done. AM iter = 232

cv : 5 out of 7 done. AM iter = 134

cv : 6 out of 7 done. AM iter = 97

cv : 7 out of 7 done. AM iter = 12

model id done, even death, mu = lambda, lambda* = 0.5. AM iter = 122

Tram. BOOT iter 54 done out of 120

cv : 1 out of 7 done. AM iter = 76

cv : 2 out of 7 done. AM iter = 76

cv : 3 out of 7 done. AM iter = 79

cv : 4 out of 7 done. AM iter = 116

cv : 5 out of 7 done. AM iter = 61

cv : 6 out of 7 done. AM iter = 52

cv : 7 out of 7 done. AM iter = 13

model id done, even death, mu = lambda, lambda* = 0.01. AM iter = 136

Tram. BOOT iter 55 done out of 120

cv : 1 out of 7 done. AM iter = 177

cv : 2 out of 7 done. AM iter = 178

cv : 3 out of 7 done. AM iter = 203

cv : 4 out of 7 done. AM iter = 233

cv : 5 out of 7 done. AM iter = 109

cv : 6 out of 7 done. AM iter = 80

cv : 7 out of 7 done. AM iter = 11

model id done, even death, mu = lambda, lambda* = 0.1. AM iter = 76

Tram. BOOT iter 56 done out of 120

cv : 1 out of 7 done. AM iter = 315

cv : 2 out of 7 done. AM iter = 305

cv : 3 out of 7 done. AM iter = 186

cv : 4 out of 7 done. AM iter = 137

cv : 5 out of 7 done. AM iter = 140

cv : 6 out of 7 done. AM iter = 108

cv : 7 out of 7 done. AM iter = 12

model id done, even death, mu = lambda, lambda* = 0.01. AM iter = 247

Tram. BOOT iter 57 done out of 120

cv : 1 out of 7 done. AM iter = 351

cv : 2 out of 7 done. AM iter = 280

cv : 3 out of 7 done. AM iter = 237

cv : 4 out of 7 done. AM iter = 188

cv : 5 out of 7 done. AM iter = 107

cv : 6 out of 7 done. AM iter = 76

cv : 7 out of 7 done. AM iter = 12

model id done, even death, mu = lambda, lambda* = 0.5. AM iter = 236

Tram. BOOT iter 58 done out of 120

cv : 1 out of 7 done. AM iter = 156

cv : 2 out of 7 done. AM iter = 232

cv : 3 out of 7 done. AM iter = 223

cv : 4 out of 7 done. AM iter = 197

cv : 5 out of 7 done. AM iter = 135

cv : 6 out of 7 done. AM iter = 98

cv : 7 out of 7 done. AM iter = 13

model id done, even death, mu = lambda, lambda* = 1. AM iter = 375

Tram. BOOT iter 59 done out of 120

cv : 1 out of 7 done. AM iter = 57

cv : 2 out of 7 done. AM iter = 57

cv : 3 out of 7 done. AM iter = 182

cv : 4 out of 7 done. AM iter = 63

cv : 5 out of 7 done. AM iter = 49

cv : 6 out of 7 done. AM iter = 41

cv : 7 out of 7 done. AM iter = 11

model id done, even death, mu = lambda, lambda* = 0.01. AM iter = 56

Tram. BOOT iter 60 done out of 120

cv : 1 out of 7 done. AM iter = 90

cv : 2 out of 7 done. AM iter = 88

cv : 3 out of 7 done. AM iter = 74

cv : 4 out of 7 done. AM iter = 80

cv : 5 out of 7 done. AM iter = 47

cv : 6 out of 7 done. AM iter = 33

cv : 7 out of 7 done. AM iter = 11

model id done, even death, mu = lambda, lambda* = 0.01. AM iter = 73

Tram. BOOT iter 61 done out of 120

cv : 1 out of 7 done. AM iter = 234

cv : 2 out of 7 done. AM iter = 250

cv : 3 out of 7 done. AM iter = 209

cv : 4 out of 7 done. AM iter = 187

cv : 5 out of 7 done. AM iter = 132

cv : 6 out of 7 done. AM iter = 95

cv : 7 out of 7 done. AM iter = 13

model id done, even death, mu = lambda, lambda* = 0.1. AM iter = 256

Tram. BOOT iter 62 done out of 120

cv : 1 out of 7 done. AM iter = 153

cv : 2 out of 7 done. AM iter = 147

cv : 3 out of 7 done. AM iter = 120

cv : 4 out of 7 done. AM iter = 118

cv : 5 out of 7 done. AM iter = 56

cv : 6 out of 7 done. AM iter = 45

cv : 7 out of 7 done. AM iter = 12

model id done, even death, mu = lambda, lambda* = 0.005. AM iter = 61

Tram. BOOT iter 63 done out of 120

cv : 1 out of 7 done. AM iter = 84

cv : 2 out of 7 done. AM iter = 80

cv : 3 out of 7 done. AM iter = 58

cv : 4 out of 7 done. AM iter = 86

cv : 5 out of 7 done. AM iter = 42

cv : 6 out of 7 done. AM iter = 32

cv : 7 out of 7 done. AM iter = 12

model id done, even death, mu = lambda, lambda* = 0.05. AM iter = 87

Tram. BOOT iter 64 done out of 120

cv : 1 out of 7 done. AM iter = 231

cv : 2 out of 7 done. AM iter = 216

cv : 3 out of 7 done. AM iter = 147

cv : 4 out of 7 done. AM iter = 134

cv : 5 out of 7 done. AM iter = 82

cv : 6 out of 7 done. AM iter = 56

cv : 7 out of 7 done. AM iter = 16

model id done, even death, mu = lambda, lambda* = 1. AM iter = 111

Tram. BOOT iter 65 done out of 120

cv : 1 out of 7 done. AM iter = 49

cv : 2 out of 7 done. AM iter = 50

cv : 3 out of 7 done. AM iter = 113

cv : 4 out of 7 done. AM iter = 83

cv : 5 out of 7 done. AM iter = 43

cv : 6 out of 7 done. AM iter = 38

cv : 7 out of 7 done. AM iter = 11

model id done, even death, mu = lambda, lambda* = 0.005. AM iter = 58

Tram. BOOT iter 66 done out of 120

cv : 1 out of 7 done. AM iter = 108

cv : 2 out of 7 done. AM iter = 106

cv : 3 out of 7 done. AM iter = 246

cv : 4 out of 7 done. AM iter = 170

cv : 5 out of 7 done. AM iter = 76

cv : 6 out of 7 done. AM iter = 57

cv : 7 out of 7 done. AM iter = 12

model id done, even death, mu = lambda, lambda* = 0.05. AM iter = 141

Tram. BOOT iter 67 done out of 120

cv : 1 out of 7 done. AM iter = 92

cv : 2 out of 7 done. AM iter = 92

cv : 3 out of 7 done. AM iter = 95

cv : 4 out of 7 done. AM iter = 138

cv : 5 out of 7 done. AM iter = 129

cv : 6 out of 7 done. AM iter = 104

cv : 7 out of 7 done. AM iter = 13

model id done, even death, mu = lambda, lambda* = 0.01. AM iter = 79

Tram. BOOT iter 68 done out of 120

cv : 1 out of 7 done. AM iter = 106

cv : 2 out of 7 done. AM iter = 95

cv : 3 out of 7 done. AM iter = 73

cv : 4 out of 7 done. AM iter = 55

cv : 5 out of 7 done. AM iter = 66

cv : 6 out of 7 done. AM iter = 51

cv : 7 out of 7 done. AM iter = 11

model id done, even death, mu = lambda, lambda* = 0.1. AM iter = 70

Tram. BOOT iter 69 done out of 120

cv : 1 out of 7 done. AM iter = 161

cv : 2 out of 7 done. AM iter = 176

cv : 3 out of 7 done. AM iter = 218

cv : 4 out of 7 done. AM iter = 139

cv : 5 out of 7 done. AM iter = 70

cv : 6 out of 7 done. AM iter = 47

cv : 7 out of 7 done. AM iter = 11

model id done, even death, mu = lambda, lambda* = 0.05. AM iter = 179

Tram. BOOT iter 70 done out of 120

cv : 1 out of 7 done. AM iter = 158

cv : 2 out of 7 done. AM iter = 162

cv : 3 out of 7 done. AM iter = 1000

cv : 4 out of 7 done. AM iter = 122

cv : 5 out of 7 done. AM iter = 100

cv : 6 out of 7 done. AM iter = 66

cv : 7 out of 7 done. AM iter = 18

model id done, even death, mu = lambda, lambda* = 0.1. AM iter = 158

Tram. BOOT iter 71 done out of 120

cv : 1 out of 7 done. AM iter = 78

cv : 2 out of 7 done. AM iter = 77

cv : 3 out of 7 done. AM iter = 82

cv : 4 out of 7 done. AM iter = 97

cv : 5 out of 7 done. AM iter = 49

cv : 6 out of 7 done. AM iter = 37

cv : 7 out of 7 done. AM iter = 11

model id done, even death, mu = lambda, lambda* = 1. AM iter = 77

Tram. BOOT iter 72 done out of 120

cv : 1 out of 7 done. AM iter = 216

cv : 2 out of 7 done. AM iter = 162

cv : 3 out of 7 done. AM iter = 206

cv : 4 out of 7 done. AM iter = 110

cv : 5 out of 7 done. AM iter = 61

cv : 6 out of 7 done. AM iter = 42

cv : 7 out of 7 done. AM iter = 14

model id done, even death, mu = lambda, lambda* = 0.1. AM iter = 141

Tram. BOOT iter 73 done out of 120

cv : 1 out of 7 done. AM iter = 51

cv : 2 out of 7 done. AM iter = 52

cv : 3 out of 7 done. AM iter = 107

cv : 4 out of 7 done. AM iter = 193

cv : 5 out of 7 done. AM iter = 57

cv : 6 out of 7 done. AM iter = 44

cv : 7 out of 7 done. AM iter = 12

model id done, even death, mu = lambda, lambda* = 0.1. AM iter = 90

Tram. BOOT iter 74 done out of 120

cv : 1 out of 7 done. AM iter = 57

cv : 2 out of 7 done. AM iter = 56

cv : 3 out of 7 done. AM iter = 50

cv : 4 out of 7 done. AM iter = 84

cv : 5 out of 7 done. AM iter = 45

cv : 6 out of 7 done. AM iter = 34

cv : 7 out of 7 done. AM iter = 12

model id done, even death, mu = lambda, lambda* = 1. AM iter = 51

Tram. BOOT iter 75 done out of 120

cv : 1 out of 7 done. AM iter = 81

cv : 2 out of 7 done. AM iter = 85

cv : 3 out of 7 done. AM iter = 128

cv : 4 out of 7 done. AM iter = 96

cv : 5 out of 7 done. AM iter = 71

cv : 6 out of 7 done. AM iter = 63

cv : 7 out of 7 done. AM iter = 13

model id done, even death, mu = lambda, lambda* = 0.5. AM iter = 97

Tram. BOOT iter 76 done out of 120

cv : 1 out of 7 done. AM iter = 141

cv : 2 out of 7 done. AM iter = 140

cv : 3 out of 7 done. AM iter = 297

cv : 4 out of 7 done. AM iter = 198

cv : 5 out of 7 done. AM iter = 62

cv : 6 out of 7 done. AM iter = 49

cv : 7 out of 7 done. AM iter = 12

model id done, even death, mu = lambda, lambda* = 0.1. AM iter = 187

Tram. BOOT iter 77 done out of 120

cv : 1 out of 7 done. AM iter = 71

cv : 2 out of 7 done. AM iter = 72

cv : 3 out of 7 done. AM iter = 86

cv : 4 out of 7 done. AM iter = 61

cv : 5 out of 7 done. AM iter = 65

cv : 6 out of 7 done. AM iter = 55

cv : 7 out of 7 done. AM iter = 14

model id done, even death, mu = lambda, lambda* = 0.005. AM iter = 44

Tram. BOOT iter 78 done out of 120

cv : 1 out of 7 done. AM iter = 76

cv : 2 out of 7 done. AM iter = 75

cv : 3 out of 7 done. AM iter = 77

cv : 4 out of 7 done. AM iter = 79

cv : 5 out of 7 done. AM iter = 73

cv : 6 out of 7 done. AM iter = 56

cv : 7 out of 7 done. AM iter = 12

model id done, even death, mu = lambda, lambda* = 0.05. AM iter = 114

Tram. BOOT iter 79 done out of 120

cv : 1 out of 7 done. AM iter = 363

cv : 2 out of 7 done. AM iter = 384

cv : 3 out of 7 done. AM iter = 185

cv : 4 out of 7 done. AM iter = 121

cv : 5 out of 7 done. AM iter = 92

cv : 6 out of 7 done. AM iter = 79

cv : 7 out of 7 done. AM iter = 12

model id done, even death, mu = lambda, lambda* = 0.5. AM iter = 121

Tram. BOOT iter 80 done out of 120

cv : 1 out of 7 done. AM iter = 76

cv : 2 out of 7 done. AM iter = 75

cv : 3 out of 7 done. AM iter = 297

cv : 4 out of 7 done. AM iter = 94

cv : 5 out of 7 done. AM iter = 54

cv : 6 out of 7 done. AM iter = 44

cv : 7 out of 7 done. AM iter = 13

model id done, even death, mu = lambda, lambda* = 0.5. AM iter = 87

Tram. BOOT iter 81 done out of 120

cv : 1 out of 7 done. AM iter = 102

cv : 2 out of 7 done. AM iter = 100

cv : 3 out of 7 done. AM iter = 92

cv : 4 out of 7 done. AM iter = 67

cv : 5 out of 7 done. AM iter = 80

cv : 6 out of 7 done. AM iter = 61

cv : 7 out of 7 done. AM iter = 15

model id done, even death, mu = lambda, lambda* = 0.01. AM iter = 145

Tram. BOOT iter 82 done out of 120

cv : 1 out of 7 done. AM iter = 56

cv : 2 out of 7 done. AM iter = 57

cv : 3 out of 7 done. AM iter = 346

cv : 4 out of 7 done. AM iter = 85

cv : 5 out of 7 done. AM iter = 48

cv : 6 out of 7 done. AM iter = 39

cv : 7 out of 7 done. AM iter = 10

model id done, even death, mu = lambda, lambda* = 0.05. AM iter = 163

Tram. BOOT iter 83 done out of 120

cv : 1 out of 7 done. AM iter = 96

cv : 2 out of 7 done. AM iter = 102

cv : 3 out of 7 done. AM iter = 94

cv : 4 out of 7 done. AM iter = 205

cv : 5 out of 7 done. AM iter = 71

cv : 6 out of 7 done. AM iter = 52

cv : 7 out of 7 done. AM iter = 10

model id done, even death, mu = lambda, lambda* = 0.005. AM iter = 101

Tram. BOOT iter 84 done out of 120

cv : 1 out of 7 done. AM iter = 271

cv : 2 out of 7 done. AM iter = 258

cv : 3 out of 7 done. AM iter = 265

cv : 4 out of 7 done. AM iter = 212

cv : 5 out of 7 done. AM iter = 104

cv : 6 out of 7 done. AM iter = 86

cv : 7 out of 7 done. AM iter = 13

model id done, even death, mu = lambda, lambda* = 0.005. AM iter = 81

Tram. BOOT iter 85 done out of 120

cv : 1 out of 7 done. AM iter = 102

cv : 2 out of 7 done. AM iter = 101

cv : 3 out of 7 done. AM iter = 97

cv : 4 out of 7 done. AM iter = 106

cv : 5 out of 7 done. AM iter = 52

cv : 6 out of 7 done. AM iter = 40

cv : 7 out of 7 done. AM iter = 12

model id done, even death, mu = lambda, lambda* = 0.01. AM iter = 75

Tram. BOOT iter 86 done out of 120

cv : 1 out of 7 done. AM iter = 114

cv : 2 out of 7 done. AM iter = 116

cv : 3 out of 7 done. AM iter = 282

cv : 4 out of 7 done. AM iter = 123

cv : 5 out of 7 done. AM iter = 74

cv : 6 out of 7 done. AM iter = 60

cv : 7 out of 7 done. AM iter = 12

model id done, even death, mu = lambda, lambda* = 0.1. AM iter = 93

Tram. BOOT iter 87 done out of 120

cv : 1 out of 7 done. AM iter = 71

cv : 2 out of 7 done. AM iter = 69

cv : 3 out of 7 done. AM iter = 254

cv : 4 out of 7 done. AM iter = 55

cv : 5 out of 7 done. AM iter = 41

cv : 6 out of 7 done. AM iter = 31

cv : 7 out of 7 done. AM iter = 10

model id done, even death, mu = lambda, lambda* = 0.01. AM iter = 53

Tram. BOOT iter 88 done out of 120

cv : 1 out of 7 done. AM iter = 128

cv : 2 out of 7 done. AM iter = 128

cv : 3 out of 7 done. AM iter = 491

cv : 4 out of 7 done. AM iter = 136

cv : 5 out of 7 done. AM iter = 123

cv : 6 out of 7 done. AM iter = 93

cv : 7 out of 7 done. AM iter = 12

model id done, even death, mu = lambda, lambda* = 0.01. AM iter = 91

Tram. BOOT iter 89 done out of 120

cv : 1 out of 7 done. AM iter = 111

cv : 2 out of 7 done. AM iter = 111

cv : 3 out of 7 done. AM iter = 122

cv : 4 out of 7 done. AM iter = 156

cv : 5 out of 7 done. AM iter = 46

cv : 6 out of 7 done. AM iter = 34

cv : 7 out of 7 done. AM iter = 11

model id done, even death, mu = lambda, lambda* = 0.05. AM iter = 113

Tram. BOOT iter 90 done out of 120

cv : 1 out of 7 done. AM iter = 99

cv : 2 out of 7 done. AM iter = 64

cv : 3 out of 7 done. AM iter = 92

cv : 4 out of 7 done. AM iter = 364

cv : 5 out of 7 done. AM iter = 30

cv : 6 out of 7 done. AM iter = 30

cv : 7 out of 7 done. AM iter = 11

model id done, even death, mu = lambda, lambda* = 0.1. AM iter = 76

Tram. BOOT iter 91 done out of 120

cv : 1 out of 7 done. AM iter = 103

cv : 2 out of 7 done. AM iter = 100

cv : 3 out of 7 done. AM iter = 140

cv : 4 out of 7 done. AM iter = 122

cv : 5 out of 7 done. AM iter = 89

cv : 6 out of 7 done. AM iter = 66

cv : 7 out of 7 done. AM iter = 12

model id done, even death, mu = lambda, lambda* = 1. AM iter = 73

Tram. BOOT iter 92 done out of 120

cv : 1 out of 7 done. AM iter = 101

cv : 2 out of 7 done. AM iter = 93

cv : 3 out of 7 done. AM iter = 84

cv : 4 out of 7 done. AM iter = 98

cv : 5 out of 7 done. AM iter = 61

cv : 6 out of 7 done. AM iter = 50

cv : 7 out of 7 done. AM iter = 13

model id done, even death, mu = lambda, lambda* = 0.1. AM iter = 82

Tram. BOOT iter 93 done out of 120

cv : 1 out of 7 done. AM iter = 167

cv : 2 out of 7 done. AM iter = 164

cv : 3 out of 7 done. AM iter = 215

cv : 4 out of 7 done. AM iter = 134

cv : 5 out of 7 done. AM iter = 101

cv : 6 out of 7 done. AM iter = 89

cv : 7 out of 7 done. AM iter = 13

model id done, even death, mu = lambda, lambda* = 0.1. AM iter = 258

Tram. BOOT iter 94 done out of 120

cv : 1 out of 7 done. AM iter = 113

cv : 2 out of 7 done. AM iter = 108

cv : 3 out of 7 done. AM iter = 82

cv : 4 out of 7 done. AM iter = 169

cv : 5 out of 7 done. AM iter = 50

cv : 6 out of 7 done. AM iter = 37

cv : 7 out of 7 done. AM iter = 13

model id done, even death, mu = lambda, lambda* = 0.05. AM iter = 150

Tram. BOOT iter 95 done out of 120

cv : 1 out of 7 done. AM iter = 181

cv : 2 out of 7 done. AM iter = 176

cv : 3 out of 7 done. AM iter = 606

cv : 4 out of 7 done. AM iter = 310

cv : 5 out of 7 done. AM iter = 151

cv : 6 out of 7 done. AM iter = 110

cv : 7 out of 7 done. AM iter = 15

model id done, even death, mu = lambda, lambda* = 0.05. AM iter = 125

Tram. BOOT iter 96 done out of 120

cv : 1 out of 7 done. AM iter = 95

cv : 2 out of 7 done. AM iter = 94

cv : 3 out of 7 done. AM iter = 80

cv : 4 out of 7 done. AM iter = 99

cv : 5 out of 7 done. AM iter = 60

cv : 6 out of 7 done. AM iter = 44

cv : 7 out of 7 done. AM iter = 11

model id done, even death, mu = lambda, lambda* = 0.005. AM iter = 97

Tram. BOOT iter 97 done out of 120

cv : 1 out of 7 done. AM iter = 154

cv : 2 out of 7 done. AM iter = 141

cv : 3 out of 7 done. AM iter = 120

cv : 4 out of 7 done. AM iter = 129

cv : 5 out of 7 done. AM iter = 116

cv : 6 out of 7 done. AM iter = 90

cv : 7 out of 7 done. AM iter = 14

model id done, even death, mu = lambda, lambda* = 1. AM iter = 79

Tram. BOOT iter 98 done out of 120

cv : 1 out of 7 done. AM iter = 118

cv : 2 out of 7 done. AM iter = 113

cv : 3 out of 7 done. AM iter = 87

cv : 4 out of 7 done. AM iter = 71

cv : 5 out of 7 done. AM iter = 46

cv : 6 out of 7 done. AM iter = 40

cv : 7 out of 7 done. AM iter = 11

model id done, even death, mu = lambda, lambda* = 1. AM iter = 68

Tram. BOOT iter 99 done out of 120

cv : 1 out of 7 done. AM iter = 88

cv : 2 out of 7 done. AM iter = 84

cv : 3 out of 7 done. AM iter = 89

cv : 4 out of 7 done. AM iter = 76

cv : 5 out of 7 done. AM iter = 60

cv : 6 out of 7 done. AM iter = 48

cv : 7 out of 7 done. AM iter = 12

model id done, even death, mu = lambda, lambda* = 0.1. AM iter = 81

Tram. BOOT iter 100 done out of 120

cv : 1 out of 7 done. AM iter = 456

cv : 2 out of 7 done. AM iter = 214

cv : 3 out of 7 done. AM iter = 126

cv : 4 out of 7 done. AM iter = 107

cv : 5 out of 7 done. AM iter = 75

cv : 6 out of 7 done. AM iter = 64

cv : 7 out of 7 done. AM iter = 12

model id done, even death, mu = lambda, lambda* = 0.5. AM iter = 74

Tram. BOOT iter 101 done out of 120

cv : 1 out of 7 done. AM iter = 201

cv : 2 out of 7 done. AM iter = 182

cv : 3 out of 7 done. AM iter = 181

cv : 4 out of 7 done. AM iter = 119

cv : 5 out of 7 done. AM iter = 67

cv : 6 out of 7 done. AM iter = 51

cv : 7 out of 7 done. AM iter = 11

model id done, even death, mu = lambda, lambda* = 0.01. AM iter = 89

Tram. BOOT iter 102 done out of 120

cv : 1 out of 7 done. AM iter = 196

cv : 2 out of 7 done. AM iter = 190

cv : 3 out of 7 done. AM iter = 159

cv : 4 out of 7 done. AM iter = 142

cv : 5 out of 7 done. AM iter = 79

cv : 6 out of 7 done. AM iter = 58

cv : 7 out of 7 done. AM iter = 11

model id done, even death, mu = lambda, lambda* = 1. AM iter = 125

Tram. BOOT iter 103 done out of 120

cv : 1 out of 7 done. AM iter = 102

cv : 2 out of 7 done. AM iter = 98

cv : 3 out of 7 done. AM iter = 88

cv : 4 out of 7 done. AM iter = 82

cv : 5 out of 7 done. AM iter = 71

cv : 6 out of 7 done. AM iter = 51

cv : 7 out of 7 done. AM iter = 14

model id done, even death, mu = lambda, lambda* = 0.005. AM iter = 68

Tram. BOOT iter 104 done out of 120

cv : 1 out of 7 done. AM iter = 73

cv : 2 out of 7 done. AM iter = 73

cv : 3 out of 7 done. AM iter = 96

cv : 4 out of 7 done. AM iter = 143

cv : 5 out of 7 done. AM iter = 42

cv : 6 out of 7 done. AM iter = 36

cv : 7 out of 7 done. AM iter = 10

model id done, even death, mu = lambda, lambda* = 0.01. AM iter = 75

Tram. BOOT iter 105 done out of 120

cv : 1 out of 7 done. AM iter = 76

cv : 2 out of 7 done. AM iter = 76

cv : 3 out of 7 done. AM iter = 110

cv : 4 out of 7 done. AM iter = 66

cv : 5 out of 7 done. AM iter = 39

cv : 6 out of 7 done. AM iter = 30

cv : 7 out of 7 done. AM iter = 11

model id done, even death, mu = lambda, lambda* = 0.01. AM iter = 59

Tram. BOOT iter 106 done out of 120

cv : 1 out of 7 done. AM iter = 54

cv : 2 out of 7 done. AM iter = 54

cv : 3 out of 7 done. AM iter = 91

cv : 4 out of 7 done. AM iter = 97

cv : 5 out of 7 done. AM iter = 64

cv : 6 out of 7 done. AM iter = 61

cv : 7 out of 7 done. AM iter = 11

model id done, even death, mu = lambda, lambda* = 0.5. AM iter = 72

Tram. BOOT iter 107 done out of 120

cv : 1 out of 7 done. AM iter = 80

cv : 2 out of 7 done. AM iter = 82

cv : 3 out of 7 done. AM iter = 345

cv : 4 out of 7 done. AM iter = 165

cv : 5 out of 7 done. AM iter = 81

cv : 6 out of 7 done. AM iter = 58

cv : 7 out of 7 done. AM iter = 12

model id done, even death, mu = lambda, lambda* = 0.01. AM iter = 118

Tram. BOOT iter 108 done out of 120

cv : 1 out of 7 done. AM iter = 104

cv : 2 out of 7 done. AM iter = 184

cv : 3 out of 7 done. AM iter = 572

cv : 4 out of 7 done. AM iter = 105

cv : 5 out of 7 done. AM iter = 61

cv : 6 out of 7 done. AM iter = 48

cv : 7 out of 7 done. AM iter = 12

model id done, even death, mu = lambda, lambda* = 0.05. AM iter = 114

Tram. BOOT iter 109 done out of 120

cv : 1 out of 7 done. AM iter = 105

cv : 2 out of 7 done. AM iter = 108

cv : 3 out of 7 done. AM iter = 191

cv : 4 out of 7 done. AM iter = 143

cv : 5 out of 7 done. AM iter = 69

cv : 6 out of 7 done. AM iter = 56

cv : 7 out of 7 done. AM iter = 11

model id done, even death, mu = lambda, lambda* = 0.1. AM iter = 137

Tram. BOOT iter 110 done out of 120

cv : 1 out of 7 done. AM iter = 76

cv : 2 out of 7 done. AM iter = 74

cv : 3 out of 7 done. AM iter = 65

cv : 4 out of 7 done. AM iter = 69

cv : 5 out of 7 done. AM iter = 51

cv : 6 out of 7 done. AM iter = 38

cv : 7 out of 7 done. AM iter = 11

model id done, even death, mu = lambda, lambda* = 0.005. AM iter = 50

Tram. BOOT iter 111 done out of 120

cv : 1 out of 7 done. AM iter = 124

cv : 2 out of 7 done. AM iter = 118

cv : 3 out of 7 done. AM iter = 86

cv : 4 out of 7 done. AM iter = 81

cv : 5 out of 7 done. AM iter = 52

cv : 6 out of 7 done. AM iter = 47

cv : 7 out of 7 done. AM iter = 11

model id done, even death, mu = lambda, lambda* = 1. AM iter = 61

Tram. BOOT iter 112 done out of 120

cv : 1 out of 7 done. AM iter = 389

cv : 2 out of 7 done. AM iter = 329

cv : 3 out of 7 done. AM iter = 259

cv : 4 out of 7 done. AM iter = 195

cv : 5 out of 7 done. AM iter = 130

cv : 6 out of 7 done. AM iter = 87

cv : 7 out of 7 done. AM iter = 12

model id done, even death, mu = lambda, lambda* = 1. AM iter = 495

Tram. BOOT iter 113 done out of 120

cv : 1 out of 7 done. AM iter = 74

cv : 2 out of 7 done. AM iter = 80

cv : 3 out of 7 done. AM iter = 132

cv : 4 out of 7 done. AM iter = 229

cv : 5 out of 7 done. AM iter = 86

cv : 6 out of 7 done. AM iter = 74

cv : 7 out of 7 done. AM iter = 14

model id done, even death, mu = lambda, lambda* = 0.5. AM iter = 87

Tram. BOOT iter 114 done out of 120

cv : 1 out of 7 done. AM iter = 175

cv : 2 out of 7 done. AM iter = 163

cv : 3 out of 7 done. AM iter = 106

cv : 4 out of 7 done. AM iter = 81

cv : 5 out of 7 done. AM iter = 68

cv : 6 out of 7 done. AM iter = 57

cv : 7 out of 7 done. AM iter = 12

model id done, even death, mu = lambda, lambda* = 0.05. AM iter = 133

Tram. BOOT iter 115 done out of 120

cv : 1 out of 7 done. AM iter = 63

cv : 2 out of 7 done. AM iter = 67

cv : 3 out of 7 done. AM iter = 326

cv : 4 out of 7 done. AM iter = 159

cv : 5 out of 7 done. AM iter = 72

cv : 6 out of 7 done. AM iter = 55

cv : 7 out of 7 done. AM iter = 13

model id done, even death, mu = lambda, lambda* = 0.5. AM iter = 68

Tram. BOOT iter 116 done out of 120

cv : 1 out of 7 done. AM iter = 89

cv : 2 out of 7 done. AM iter = 88

cv : 3 out of 7 done. AM iter = 94

cv : 4 out of 7 done. AM iter = 82

cv : 5 out of 7 done. AM iter = 41

cv : 6 out of 7 done. AM iter = 35

cv : 7 out of 7 done. AM iter = 11

model id done, even death, mu = lambda, lambda* = 0.1. AM iter = 89

Tram. BOOT iter 117 done out of 120

cv : 1 out of 7 done. AM iter = 73

cv : 2 out of 7 done. AM iter = 95

cv : 3 out of 7 done. AM iter = 145

cv : 4 out of 7 done. AM iter = 194

cv : 5 out of 7 done. AM iter = 103

cv : 6 out of 7 done. AM iter = 70

cv : 7 out of 7 done. AM iter = 14

model id done, even death, mu = lambda, lambda* = 0.01. AM iter = 124

Tram. BOOT iter 118 done out of 120

cv : 1 out of 7 done. AM iter = 54

cv : 2 out of 7 done. AM iter = 54

cv : 3 out of 7 done. AM iter = 716

cv : 4 out of 7 done. AM iter = 229

cv : 5 out of 7 done. AM iter = 79

cv : 6 out of 7 done. AM iter = 64

cv : 7 out of 7 done. AM iter = 12

model id done, even death, mu = lambda, lambda* = 0.01. AM iter = 50

Tram. BOOT iter 119 done out of 120

cv : 1 out of 7 done. AM iter = 117

cv : 2 out of 7 done. AM iter = 113

cv : 3 out of 7 done. AM iter = 90

cv : 4 out of 7 done. AM iter = 73

cv : 5 out of 7 done. AM iter = 34

cv : 6 out of 7 done. AM iter = 31

cv : 7 out of 7 done. AM iter = 11

model id done, even death, mu = lambda, lambda* = 1. AM iter = 110

Tram. BOOT iter 120 done out of 120

cv : 1 out of 7 done. AM iter = 62

cv : 2 out of 7 done. AM iter = 62

cv : 3 out of 7 done. AM iter = 61

cv : 4 out of 7 done. AM iter = 59

cv : 5 out of 7 done. AM iter = 48

cv : 6 out of 7 done. AM iter = 32

cv : 7 out of 7 done. AM iter = 11

model id done, even death, mu = lambda, lambda* = 0.01. AM iter = 62

BEZ BOOT iter 1 done out of 120

cv : 1 out of 7 done. AM iter = 60

cv : 2 out of 7 done. AM iter = 59

cv : 3 out of 7 done. AM iter = 57

cv : 4 out of 7 done. AM iter = 54

cv : 5 out of 7 done. AM iter = 44

cv : 6 out of 7 done. AM iter = 25

cv : 7 out of 7 done. AM iter = 11

model id done, even death, mu = lambda, lambda* = 0.005. AM iter = 32

BEZ BOOT iter 2 done out of 120

cv : 1 out of 7 done. AM iter = 65

cv : 2 out of 7 done. AM iter = 65

cv : 3 out of 7 done. AM iter = 63

cv : 4 out of 7 done. AM iter = 111

cv : 5 out of 7 done. AM iter = 47

cv : 6 out of 7 done. AM iter = 35

cv : 7 out of 7 done. AM iter = 12

model id done, even death, mu = lambda, lambda* = 0.05. AM iter = 155

BEZ BOOT iter 3 done out of 120

cv : 1 out of 7 done. AM iter = 147

cv : 2 out of 7 done. AM iter = 144

cv : 3 out of 7 done. AM iter = 129

cv : 4 out of 7 done. AM iter = 109

cv : 5 out of 7 done. AM iter = 48

cv : 6 out of 7 done. AM iter = 35

cv : 7 out of 7 done. AM iter = 12

model id done, even death, mu = lambda, lambda* = 0.005. AM iter = 38

BEZ BOOT iter 4 done out of 120

cv : 1 out of 7 done. AM iter = 76

cv : 2 out of 7 done. AM iter = 77

cv : 3 out of 7 done. AM iter = 72

cv : 4 out of 7 done. AM iter = 200

cv : 5 out of 7 done. AM iter = 42

cv : 6 out of 7 done. AM iter = 26

cv : 7 out of 7 done. AM iter = 12

model id done, even death, mu = lambda, lambda* = 0.01. AM iter = 59

BEZ BOOT iter 5 done out of 120

cv : 1 out of 7 done. AM iter = 75

cv : 2 out of 7 done. AM iter = 77

cv : 3 out of 7 done. AM iter = 98

cv : 4 out of 7 done. AM iter = 254

cv : 5 out of 7 done. AM iter = 64

cv : 6 out of 7 done. AM iter = 53

cv : 7 out of 7 done. AM iter = 13

model id done, even death, mu = lambda, lambda* = 0.005. AM iter = 92

BEZ BOOT iter 6 done out of 120

cv : 1 out of 7 done. AM iter = 102

cv : 2 out of 7 done. AM iter = 102

cv : 3 out of 7 done. AM iter = 143

cv : 4 out of 7 done. AM iter = 152

cv : 5 out of 7 done. AM iter = 54

cv : 6 out of 7 done. AM iter = 29

cv : 7 out of 7 done. AM iter = 12

model id done, even death, mu = lambda, lambda* = 0.005. AM iter = 28

BEZ BOOT iter 7 done out of 120

cv : 1 out of 7 done. AM iter = 103

cv : 2 out of 7 done. AM iter = 102

cv : 3 out of 7 done. AM iter = 94

cv : 4 out of 7 done. AM iter = 85

cv : 5 out of 7 done. AM iter = 51

cv : 6 out of 7 done. AM iter = 27

cv : 7 out of 7 done. AM iter = 11

model id done, even death, mu = lambda, lambda* = 0.01. AM iter = 88

BEZ BOOT iter 8 done out of 120

cv : 1 out of 7 done. AM iter = 75

cv : 2 out of 7 done. AM iter = 75

cv : 3 out of 7 done. AM iter = 75

cv : 4 out of 7 done. AM iter = 83

cv : 5 out of 7 done. AM iter = 65

cv : 6 out of 7 done. AM iter = 26

cv : 7 out of 7 done. AM iter = 11

model id done, even death, mu = lambda, lambda* = 0.05. AM iter = 104

BEZ BOOT iter 9 done out of 120

cv : 1 out of 7 done. AM iter = 138

cv : 2 out of 7 done. AM iter = 138

cv : 3 out of 7 done. AM iter = 133

cv : 4 out of 7 done. AM iter = 130

cv : 5 out of 7 done. AM iter = 46

cv : 6 out of 7 done. AM iter = 30

cv : 7 out of 7 done. AM iter = 11

model id done, even death, mu = lambda, lambda* = 0.005. AM iter = 42

BEZ BOOT iter 10 done out of 120

cv : 1 out of 7 done. AM iter = 211

cv : 2 out of 7 done. AM iter = 197

cv : 3 out of 7 done. AM iter = 154

cv : 4 out of 7 done. AM iter = 115

cv : 5 out of 7 done. AM iter = 71

cv : 6 out of 7 done. AM iter = 38

cv : 7 out of 7 done. AM iter = 11

model id done, even death, mu = lambda, lambda* = 0.005. AM iter = 67

BEZ BOOT iter 11 done out of 120

cv : 1 out of 7 done. AM iter = 52

cv : 2 out of 7 done. AM iter = 49

cv : 3 out of 7 done. AM iter = 71

cv : 4 out of 7 done. AM iter = 57

cv : 5 out of 7 done. AM iter = 35

cv : 6 out of 7 done. AM iter = 26

cv : 7 out of 7 done. AM iter = 10

model id done, even death, mu = lambda, lambda* = 0.01. AM iter = 45

BEZ BOOT iter 12 done out of 120

cv : 1 out of 7 done. AM iter = 69

cv : 2 out of 7 done. AM iter = 69

cv : 3 out of 7 done. AM iter = 67

cv : 4 out of 7 done. AM iter = 103

cv : 5 out of 7 done. AM iter = 74

cv : 6 out of 7 done. AM iter = 35

cv : 7 out of 7 done. AM iter = 12

model id done, even death, mu = lambda, lambda* = 0.005. AM iter = 35

BEZ BOOT iter 13 done out of 120

cv : 1 out of 7 done. AM iter = 170

cv : 2 out of 7 done. AM iter = 169

cv : 3 out of 7 done. AM iter = 160

cv : 4 out of 7 done. AM iter = 208

cv : 5 out of 7 done. AM iter = 41

cv : 6 out of 7 done. AM iter = 32

cv : 7 out of 7 done. AM iter = 13

model id done, even death, mu = lambda, lambda* = 0.1. AM iter = 134

BEZ BOOT iter 14 done out of 120

cv : 1 out of 7 done. AM iter = 98

cv : 2 out of 7 done. AM iter = 61

cv : 3 out of 7 done. AM iter = 95

cv : 4 out of 7 done. AM iter = 224

cv : 5 out of 7 done. AM iter = 35

cv : 6 out of 7 done. AM iter = 20

cv : 7 out of 7 done. AM iter = 11

model id done, even death, mu = lambda, lambda* = 0.005. AM iter = 28

BEZ BOOT iter 15 done out of 120

cv : 1 out of 7 done. AM iter = 114

cv : 2 out of 7 done. AM iter = 113

cv : 3 out of 7 done. AM iter = 106

cv : 4 out of 7 done. AM iter = 95

cv : 5 out of 7 done. AM iter = 45

cv : 6 out of 7 done. AM iter = 37

cv : 7 out of 7 done. AM iter = 12

model id done, even death, mu = lambda, lambda* = 0.01. AM iter = 51

BEZ BOOT iter 16 done out of 120

cv : 1 out of 7 done. AM iter = 90

cv : 2 out of 7 done. AM iter = 89

cv : 3 out of 7 done. AM iter = 83

cv : 4 out of 7 done. AM iter = 96

cv : 5 out of 7 done. AM iter = 60

cv : 6 out of 7 done. AM iter = 1000

cv : 7 out of 7 done. AM iter = 13

model id done, even death, mu = lambda, lambda* = 0.01. AM iter = 73

BEZ BOOT iter 17 done out of 120

cv : 1 out of 7 done. AM iter = 47

cv : 2 out of 7 done. AM iter = 47

cv : 3 out of 7 done. AM iter = 42

cv : 4 out of 7 done. AM iter = 49

cv : 5 out of 7 done. AM iter = 24

cv : 6 out of 7 done. AM iter = 18

cv : 7 out of 7 done. AM iter = 10

model id done, even death, mu = lambda, lambda* = 0.01. AM iter = 39

BEZ BOOT iter 18 done out of 120

cv : 1 out of 7 done. AM iter = 129

cv : 2 out of 7 done. AM iter = 129

cv : 3 out of 7 done. AM iter = 129

cv : 4 out of 7 done. AM iter = 131

cv : 5 out of 7 done. AM iter = 57

cv : 6 out of 7 done. AM iter = 41

cv : 7 out of 7 done. AM iter = 12

model id done, even death, mu = lambda, lambda* = 0.01. AM iter = 51

BEZ BOOT iter 19 done out of 120

cv : 1 out of 7 done. AM iter = 92

cv : 2 out of 7 done. AM iter = 94

cv : 3 out of 7 done. AM iter = 120

cv : 4 out of 7 done. AM iter = 196

cv : 5 out of 7 done. AM iter = 46

cv : 6 out of 7 done. AM iter = 30

cv : 7 out of 7 done. AM iter = 12

model id done, even death, mu = lambda, lambda* = 0.01. AM iter = 56

BEZ BOOT iter 20 done out of 120

cv : 1 out of 7 done. AM iter = 48

cv : 2 out of 7 done. AM iter = 47

cv : 3 out of 7 done. AM iter = 53

cv : 4 out of 7 done. AM iter = 82

cv : 5 out of 7 done. AM iter = 41

cv : 6 out of 7 done. AM iter = 28

cv : 7 out of 7 done. AM iter = 11

model id done, even death, mu = lambda, lambda* = 0.01. AM iter = 106

BEZ BOOT iter 21 done out of 120

cv : 1 out of 7 done. AM iter = 204

cv : 2 out of 7 done. AM iter = 201

cv : 3 out of 7 done. AM iter = 175

cv : 4 out of 7 done. AM iter = 121

cv : 5 out of 7 done. AM iter = 76

cv : 6 out of 7 done. AM iter = 58

cv : 7 out of 7 done. AM iter = 13

model id done, even death, mu = lambda, lambda* = 0.01. AM iter = 100

BEZ BOOT iter 22 done out of 120

cv : 1 out of 7 done. AM iter = 165

cv : 2 out of 7 done. AM iter = 164

cv : 3 out of 7 done. AM iter = 156

cv : 4 out of 7 done. AM iter = 149

cv : 5 out of 7 done. AM iter = 43

cv : 6 out of 7 done. AM iter = 28

cv : 7 out of 7 done. AM iter = 11

model id done, even death, mu = lambda, lambda* = 0.005. AM iter = 46

BEZ BOOT iter 23 done out of 120

cv : 1 out of 7 done. AM iter = 85

cv : 2 out of 7 done. AM iter = 84

cv : 3 out of 7 done. AM iter = 81

cv : 4 out of 7 done. AM iter = 78

cv : 5 out of 7 done. AM iter = 45

cv : 6 out of 7 done. AM iter = 45

cv : 7 out of 7 done. AM iter = 13

model id done, even death, mu = lambda, lambda* = 0.01. AM iter = 50

BEZ BOOT iter 24 done out of 120

cv : 1 out of 7 done. AM iter = 135

cv : 2 out of 7 done. AM iter = 134

cv : 3 out of 7 done. AM iter = 167

cv : 4 out of 7 done. AM iter = 102

cv : 5 out of 7 done. AM iter = 64

cv : 6 out of 7 done. AM iter = 43

cv : 7 out of 7 done. AM iter = 12

model id done, even death, mu = lambda, lambda* = 0.01. AM iter = 49

BEZ BOOT iter 25 done out of 120

cv : 1 out of 7 done. AM iter = 86

cv : 2 out of 7 done. AM iter = 85

cv : 3 out of 7 done. AM iter = 74

cv : 4 out of 7 done. AM iter = 64

cv : 5 out of 7 done. AM iter = 72

cv : 6 out of 7 done. AM iter = 51

cv : 7 out of 7 done. AM iter = 12

model id done, even death, mu = lambda, lambda* = 0.01. AM iter = 120

BEZ BOOT iter 26 done out of 120

cv : 1 out of 7 done. AM iter = 99

cv : 2 out of 7 done. AM iter = 100

cv : 3 out of 7 done. AM iter = 345

cv : 4 out of 7 done. AM iter = 153

cv : 5 out of 7 done. AM iter = 64

cv : 6 out of 7 done. AM iter = 36

cv : 7 out of 7 done. AM iter = 12

model id done, even death, mu = lambda, lambda* = 0.01. AM iter = 66

BEZ BOOT iter 27 done out of 120

cv : 1 out of 7 done. AM iter = 59

cv : 2 out of 7 done. AM iter = 59

cv : 3 out of 7 done. AM iter = 58

cv : 4 out of 7 done. AM iter = 75

cv : 5 out of 7 done. AM iter = 64

cv : 6 out of 7 done. AM iter = 40

cv : 7 out of 7 done. AM iter = 14

model id done, even death, mu = lambda, lambda* = 0.005. AM iter = 49

BEZ BOOT iter 28 done out of 120

cv : 1 out of 7 done. AM iter = 74

cv : 2 out of 7 done. AM iter = 74

cv : 3 out of 7 done. AM iter = 73

cv : 4 out of 7 done. AM iter = 155

cv : 5 out of 7 done. AM iter = 80

cv : 6 out of 7 done. AM iter = 50

cv : 7 out of 7 done. AM iter = 13

model id done, even death, mu = lambda, lambda* = 0.01. AM iter = 66

BEZ BOOT iter 29 done out of 120

cv : 1 out of 7 done. AM iter = 138

cv : 2 out of 7 done. AM iter = 135

cv : 3 out of 7 done. AM iter = 106

cv : 4 out of 7 done. AM iter = 121

cv : 5 out of 7 done. AM iter = 53

cv : 6 out of 7 done. AM iter = 42

cv : 7 out of 7 done. AM iter = 11

model id done, even death, mu = lambda, lambda* = 0.05. AM iter = 173

BEZ BOOT iter 30 done out of 120

cv : 1 out of 7 done. AM iter = 175

cv : 2 out of 7 done. AM iter = 168

cv : 3 out of 7 done. AM iter = 140

cv : 4 out of 7 done. AM iter = 138

cv : 5 out of 7 done. AM iter = 49

cv : 6 out of 7 done. AM iter = 35

cv : 7 out of 7 done. AM iter = 12

model id done, even death, mu = lambda, lambda* = 0.05. AM iter = 107

BEZ BOOT iter 31 done out of 120

cv : 1 out of 7 done. AM iter = 75

cv : 2 out of 7 done. AM iter = 75

cv : 3 out of 7 done. AM iter = 69

cv : 4 out of 7 done. AM iter = 65

cv : 5 out of 7 done. AM iter = 43

cv : 6 out of 7 done. AM iter = 26

cv : 7 out of 7 done. AM iter = 11

model id done, even death, mu = lambda, lambda* = 0.01. AM iter = 57

BEZ BOOT iter 32 done out of 120

cv : 1 out of 7 done. AM iter = 99

cv : 2 out of 7 done. AM iter = 98

cv : 3 out of 7 done. AM iter = 85

cv : 4 out of 7 done. AM iter = 76

cv : 5 out of 7 done. AM iter = 32

cv : 6 out of 7 done. AM iter = 22

cv : 7 out of 7 done. AM iter = 11

model id done, even death, mu = lambda, lambda* = 0.005. AM iter = 26

BEZ BOOT iter 33 done out of 120

cv : 1 out of 7 done. AM iter = 269

cv : 2 out of 7 done. AM iter = 251

cv : 3 out of 7 done. AM iter = 169

cv : 4 out of 7 done. AM iter = 114

cv : 5 out of 7 done. AM iter = 65

cv : 6 out of 7 done. AM iter = 33

cv : 7 out of 7 done. AM iter = 12

model id done, even death, mu = lambda, lambda* = 0.005. AM iter = 39

BEZ BOOT iter 34 done out of 120

cv : 1 out of 7 done. AM iter = 78

cv : 2 out of 7 done. AM iter = 78

cv : 3 out of 7 done. AM iter = 74

cv : 4 out of 7 done. AM iter = 69

cv : 5 out of 7 done. AM iter = 45

cv : 6 out of 7 done. AM iter = 35

cv : 7 out of 7 done. AM iter = 12

model id done, even death, mu = lambda, lambda* = 0.01. AM iter = 53

BEZ BOOT iter 35 done out of 120

cv : 1 out of 7 done. AM iter = 72

cv : 2 out of 7 done. AM iter = 73

cv : 3 out of 7 done. AM iter = 71

cv : 4 out of 7 done. AM iter = 71

cv : 5 out of 7 done. AM iter = 52

cv : 6 out of 7 done. AM iter = 38

cv : 7 out of 7 done. AM iter = 12

model id done, even death, mu = lambda, lambda* = 0.005. AM iter = 38

BEZ BOOT iter 36 done out of 120

cv : 1 out of 7 done. AM iter = 109

cv : 2 out of 7 done. AM iter = 108

cv : 3 out of 7 done. AM iter = 101

cv : 4 out of 7 done. AM iter = 94

cv : 5 out of 7 done. AM iter = 42

cv : 6 out of 7 done. AM iter = 38

cv : 7 out of 7 done. AM iter = 12

model id done, even death, mu = lambda, lambda* = 0.01. AM iter = 35

BEZ BOOT iter 37 done out of 120

cv : 1 out of 7 done. AM iter = 125

cv : 2 out of 7 done. AM iter = 125

cv : 3 out of 7 done. AM iter = 124

cv : 4 out of 7 done. AM iter = 131

cv : 5 out of 7 done. AM iter = 67

cv : 6 out of 7 done. AM iter = 57

cv : 7 out of 7 done. AM iter = 13

model id done, even death, mu = lambda, lambda* = 0.05. AM iter = 167

BEZ BOOT iter 38 done out of 120

cv : 1 out of 7 done. AM iter = 72

cv : 2 out of 7 done. AM iter = 72

cv : 3 out of 7 done. AM iter = 75

cv : 4 out of 7 done. AM iter = 182

cv : 5 out of 7 done. AM iter = 41

cv : 6 out of 7 done. AM iter = 38

cv : 7 out of 7 done. AM iter = 13

model id done, even death, mu = lambda, lambda* = 0.01. AM iter = 57

BEZ BOOT iter 39 done out of 120

cv : 1 out of 7 done. AM iter = 132

cv : 2 out of 7 done. AM iter = 132

cv : 3 out of 7 done. AM iter = 126

cv : 4 out of 7 done. AM iter = 121

cv : 5 out of 7 done. AM iter = 40

cv : 6 out of 7 done. AM iter = 31

cv : 7 out of 7 done. AM iter = 12

model id done, even death, mu = lambda, lambda* = 0.01. AM iter = 47

BEZ BOOT iter 40 done out of 120

cv : 1 out of 7 done. AM iter = 59

cv : 2 out of 7 done. AM iter = 59

cv : 3 out of 7 done. AM iter = 56

cv : 4 out of 7 done. AM iter = 53

cv : 5 out of 7 done. AM iter = 40

cv : 6 out of 7 done. AM iter = 26

cv : 7 out of 7 done. AM iter = 10

model id done, even death, mu = lambda, lambda* = 0.01. AM iter = 56

BEZ BOOT iter 41 done out of 120

cv : 1 out of 7 done. AM iter = 76

cv : 2 out of 7 done. AM iter = 75

cv : 3 out of 7 done. AM iter = 73

cv : 4 out of 7 done. AM iter = 74

cv : 5 out of 7 done. AM iter = 43

cv : 6 out of 7 done. AM iter = 25

cv : 7 out of 7 done. AM iter = 12

model id done, even death, mu = lambda, lambda* = 0.01. AM iter = 61

BEZ BOOT iter 42 done out of 120

cv : 1 out of 7 done. AM iter = 108

cv : 2 out of 7 done. AM iter = 97

cv : 3 out of 7 done. AM iter = 76

cv : 4 out of 7 done. AM iter = 69

cv : 5 out of 7 done. AM iter = 49

cv : 6 out of 7 done. AM iter = 29

cv : 7 out of 7 done. AM iter = 11

model id done, even death, mu = lambda, lambda* = 0.01. AM iter = 56

BEZ BOOT iter 43 done out of 120

cv : 1 out of 7 done. AM iter = 107

cv : 2 out of 7 done. AM iter = 107

cv : 3 out of 7 done. AM iter = 117

cv : 4 out of 7 done. AM iter = 115

cv : 5 out of 7 done. AM iter = 39

cv : 6 out of 7 done. AM iter = 29

cv : 7 out of 7 done. AM iter = 12

model id done, even death, mu = lambda, lambda* = 0.01. AM iter = 52

BEZ BOOT iter 44 done out of 120

cv : 1 out of 7 done. AM iter = 146

cv : 2 out of 7 done. AM iter = 145

cv : 3 out of 7 done. AM iter = 143

cv : 4 out of 7 done. AM iter = 135

cv : 5 out of 7 done. AM iter = 40

cv : 6 out of 7 done. AM iter = 27

cv : 7 out of 7 done. AM iter = 11

model id done, even death, mu = lambda, lambda* = 0.01. AM iter = 43

BEZ BOOT iter 45 done out of 120

cv : 1 out of 7 done. AM iter = 71

cv : 2 out of 7 done. AM iter = 70

cv : 3 out of 7 done. AM iter = 70

cv : 4 out of 7 done. AM iter = 139

cv : 5 out of 7 done. AM iter = 39

cv : 6 out of 7 done. AM iter = 28

cv : 7 out of 7 done. AM iter = 11

model id done, even death, mu = lambda, lambda* = 0.01. AM iter = 42

BEZ BOOT iter 46 done out of 120

cv : 1 out of 7 done. AM iter = 179

cv : 2 out of 7 done. AM iter = 174

cv : 3 out of 7 done. AM iter = 157

cv : 4 out of 7 done. AM iter = 117

cv : 5 out of 7 done. AM iter = 85

cv : 6 out of 7 done. AM iter = 61

cv : 7 out of 7 done. AM iter = 13

model id done, even death, mu = lambda, lambda* = 0.01. AM iter = 78

BEZ BOOT iter 47 done out of 120

cv : 1 out of 7 done. AM iter = 83

cv : 2 out of 7 done. AM iter = 83

cv : 3 out of 7 done. AM iter = 79

cv : 4 out of 7 done. AM iter = 131

cv : 5 out of 7 done. AM iter = 61

cv : 6 out of 7 done. AM iter = 40

cv : 7 out of 7 done. AM iter = 11

model id done, even death, mu = lambda, lambda* = 0.05. AM iter = 144

BEZ BOOT iter 48 done out of 120

cv : 1 out of 7 done. AM iter = 144

cv : 2 out of 7 done. AM iter = 144

cv : 3 out of 7 done. AM iter = 146

cv : 4 out of 7 done. AM iter = 168

cv : 5 out of 7 done. AM iter = 85

cv : 6 out of 7 done. AM iter = 33

cv : 7 out of 7 done. AM iter = 13

model id done, even death, mu = lambda, lambda* = 0.005. AM iter = 44

BEZ BOOT iter 49 done out of 120

cv : 1 out of 7 done. AM iter = 116

cv : 2 out of 7 done. AM iter = 115

cv : 3 out of 7 done. AM iter = 102

cv : 4 out of 7 done. AM iter = 90

cv : 5 out of 7 done. AM iter = 54

cv : 6 out of 7 done. AM iter = 25

cv : 7 out of 7 done. AM iter = 11

model id done, even death, mu = lambda, lambda* = 0.01. AM iter = 57

BEZ BOOT iter 50 done out of 120

cv : 1 out of 7 done. AM iter = 246

cv : 2 out of 7 done. AM iter = 241

cv : 3 out of 7 done. AM iter = 203

cv : 4 out of 7 done. AM iter = 167

cv : 5 out of 7 done. AM iter = 63

cv : 6 out of 7 done. AM iter = 37

cv : 7 out of 7 done. AM iter = 12

model id done, even death, mu = lambda, lambda* = 0.01. AM iter = 112

BEZ BOOT iter 51 done out of 120

cv : 1 out of 7 done. AM iter = 33

cv : 2 out of 7 done. AM iter = 43

cv : 3 out of 7 done. AM iter = 80

cv : 4 out of 7 done. AM iter = 130

cv : 5 out of 7 done. AM iter = 50

cv : 6 out of 7 done. AM iter = 37

cv : 7 out of 7 done. AM iter = 12

model id done, even death, mu = lambda, lambda* = 0.05. AM iter = 99

BEZ BOOT iter 52 done out of 120

cv : 1 out of 7 done. AM iter = 108

cv : 2 out of 7 done. AM iter = 110

cv : 3 out of 7 done. AM iter = 199

cv : 4 out of 7 done. AM iter = 120

cv : 5 out of 7 done. AM iter = 52

cv : 6 out of 7 done. AM iter = 36

cv : 7 out of 7 done. AM iter = 12

model id done, even death, mu = lambda, lambda* = 0.01. AM iter = 40

BEZ BOOT iter 53 done out of 120

cv : 1 out of 7 done. AM iter = 95

cv : 2 out of 7 done. AM iter = 94

cv : 3 out of 7 done. AM iter = 92

cv : 4 out of 7 done. AM iter = 169

cv : 5 out of 7 done. AM iter = 47

cv : 6 out of 7 done. AM iter = 33

cv : 7 out of 7 done. AM iter = 12

model id done, even death, mu = lambda, lambda* = 0.01. AM iter = 59

BEZ BOOT iter 54 done out of 120

cv : 1 out of 7 done. AM iter = 94

cv : 2 out of 7 done. AM iter = 94

cv : 3 out of 7 done. AM iter = 95

cv : 4 out of 7 done. AM iter = 119

cv : 5 out of 7 done. AM iter = 39

cv : 6 out of 7 done. AM iter = 27

cv : 7 out of 7 done. AM iter = 11

model id done, even death, mu = lambda, lambda* = 0.01. AM iter = 94

BEZ BOOT iter 55 done out of 120

cv : 1 out of 7 done. AM iter = 55

cv : 2 out of 7 done. AM iter = 54

cv : 3 out of 7 done. AM iter = 51

cv : 4 out of 7 done. AM iter = 47

cv : 5 out of 7 done. AM iter = 36

cv : 6 out of 7 done. AM iter = 29

cv : 7 out of 7 done. AM iter = 10

model id done, even death, mu = lambda, lambda* = 0.01. AM iter = 48

BEZ BOOT iter 56 done out of 120

cv : 1 out of 7 done. AM iter = 104

cv : 2 out of 7 done. AM iter = 99

cv : 3 out of 7 done. AM iter = 85

cv : 4 out of 7 done. AM iter = 113

cv : 5 out of 7 done. AM iter = 67

cv : 6 out of 7 done. AM iter = 44

cv : 7 out of 7 done. AM iter = 12

model id done, even death, mu = lambda, lambda* = 0.01. AM iter = 68

BEZ BOOT iter 57 done out of 120

cv : 1 out of 7 done. AM iter = 106

cv : 2 out of 7 done. AM iter = 105

cv : 3 out of 7 done. AM iter = 101

cv : 4 out of 7 done. AM iter = 112

cv : 5 out of 7 done. AM iter = 61

cv : 6 out of 7 done. AM iter = 35

cv : 7 out of 7 done. AM iter = 11

model id done, even death, mu = lambda, lambda* = 0.1. AM iter = 80

BEZ BOOT iter 58 done out of 120

cv : 1 out of 7 done. AM iter = 59

cv : 2 out of 7 done. AM iter = 59

cv : 3 out of 7 done. AM iter = 59

cv : 4 out of 7 done. AM iter = 60

cv : 5 out of 7 done. AM iter = 61

cv : 6 out of 7 done. AM iter = 39

cv : 7 out of 7 done. AM iter = 13

model id done, even death, mu = lambda, lambda* = 0.01. AM iter = 66

BEZ BOOT iter 59 done out of 120

cv : 1 out of 7 done. AM iter = 95

cv : 2 out of 7 done. AM iter = 96

cv : 3 out of 7 done. AM iter = 106

cv : 4 out of 7 done. AM iter = 102

cv : 5 out of 7 done. AM iter = 56

cv : 6 out of 7 done. AM iter = 44

cv : 7 out of 7 done. AM iter = 13

model id done, even death, mu = lambda, lambda* = 0.005. AM iter = 47

BEZ BOOT iter 60 done out of 120

cv : 1 out of 7 done. AM iter = 182

cv : 2 out of 7 done. AM iter = 181

cv : 3 out of 7 done. AM iter = 173

cv : 4 out of 7 done. AM iter = 283

cv : 5 out of 7 done. AM iter = 51

cv : 6 out of 7 done. AM iter = 48

cv : 7 out of 7 done. AM iter = 12

model id done, even death, mu = lambda, lambda* = 0.01. AM iter = 83

BEZ BOOT iter 61 done out of 120

cv : 1 out of 7 done. AM iter = 95

cv : 2 out of 7 done. AM iter = 94

cv : 3 out of 7 done. AM iter = 87

cv : 4 out of 7 done. AM iter = 81

cv : 5 out of 7 done. AM iter = 33

cv : 6 out of 7 done. AM iter = 24

cv : 7 out of 7 done. AM iter = 11

model id done, even death, mu = lambda, lambda* = 0.01. AM iter = 40

BEZ BOOT iter 62 done out of 120

cv : 1 out of 7 done. AM iter = 93

cv : 2 out of 7 done. AM iter = 93

cv : 3 out of 7 done. AM iter = 92

cv : 4 out of 7 done. AM iter = 111

cv : 5 out of 7 done. AM iter = 58

cv : 6 out of 7 done. AM iter = 46

cv : 7 out of 7 done. AM iter = 14

model id done, even death, mu = lambda, lambda* = 0.01. AM iter = 56

BEZ BOOT iter 63 done out of 120

cv : 1 out of 7 done. AM iter = 63

cv : 2 out of 7 done. AM iter = 63

cv : 3 out of 7 done. AM iter = 66

cv : 4 out of 7 done. AM iter = 213

cv : 5 out of 7 done. AM iter = 34

cv : 6 out of 7 done. AM iter = 27

cv : 7 out of 7 done. AM iter = 11

model id done, even death, mu = lambda, lambda* = 0.1. AM iter = 90

BEZ BOOT iter 64 done out of 120

cv : 1 out of 7 done. AM iter = 49

cv : 2 out of 7 done. AM iter = 50

cv : 3 out of 7 done. AM iter = 114

cv : 4 out of 7 done. AM iter = 99

cv : 5 out of 7 done. AM iter = 36

cv : 6 out of 7 done. AM iter = 26

cv : 7 out of 7 done. AM iter = 11

model id done, even death, mu = lambda, lambda* = 0.01. AM iter = 44

BEZ BOOT iter 65 done out of 120

cv : 1 out of 7 done. AM iter = 158

cv : 2 out of 7 done. AM iter = 157

cv : 3 out of 7 done. AM iter = 165

cv : 4 out of 7 done. AM iter = 204

cv : 5 out of 7 done. AM iter = 34

cv : 6 out of 7 done. AM iter = 27

cv : 7 out of 7 done. AM iter = 11

model id done, even death, mu = lambda, lambda* = 0.005. AM iter = 42

BEZ BOOT iter 66 done out of 120

cv : 1 out of 7 done. AM iter = 121

cv : 2 out of 7 done. AM iter = 122

cv : 3 out of 7 done. AM iter = 119

cv : 4 out of 7 done. AM iter = 139

cv : 5 out of 7 done. AM iter = 52

cv : 6 out of 7 done. AM iter = 37

cv : 7 out of 7 done. AM iter = 12

model id done, even death, mu = lambda, lambda* = 0.1. AM iter = 78

BEZ BOOT iter 67 done out of 120

cv : 1 out of 7 done. AM iter = 89

cv : 2 out of 7 done. AM iter = 88

cv : 3 out of 7 done. AM iter = 217

cv : 4 out of 7 done. AM iter = 98

cv : 5 out of 7 done. AM iter = 43

cv : 6 out of 7 done. AM iter = 32

cv : 7 out of 7 done. AM iter = 13

model id done, even death, mu = lambda, lambda* = 0.01. AM iter = 51

BEZ BOOT iter 68 done out of 120

cv : 1 out of 7 done. AM iter = 93

cv : 2 out of 7 done. AM iter = 92

cv : 3 out of 7 done. AM iter = 91

cv : 4 out of 7 done. AM iter = 60

cv : 5 out of 7 done. AM iter = 34

cv : 6 out of 7 done. AM iter = 31

cv : 7 out of 7 done. AM iter = 11

model id done, even death, mu = lambda, lambda* = 0.005. AM iter = 46

BEZ BOOT iter 69 done out of 120

cv : 1 out of 7 done. AM iter = 117

cv : 2 out of 7 done. AM iter = 117

cv : 3 out of 7 done. AM iter = 115

cv : 4 out of 7 done. AM iter = 113

cv : 5 out of 7 done. AM iter = 29

cv : 6 out of 7 done. AM iter = 23

cv : 7 out of 7 done. AM iter = 11

model id done, even death, mu = lambda, lambda* = 0.01. AM iter = 37

BEZ BOOT iter 70 done out of 120

cv : 1 out of 7 done. AM iter = 90

cv : 2 out of 7 done. AM iter = 90

cv : 3 out of 7 done. AM iter = 142

cv : 4 out of 7 done. AM iter = 121

cv : 5 out of 7 done. AM iter = 42

cv : 6 out of 7 done. AM iter = 25

cv : 7 out of 7 done. AM iter = 12

model id done, even death, mu = lambda, lambda* = 0.005. AM iter = 37

BEZ BOOT iter 71 done out of 120

cv : 1 out of 7 done. AM iter = 346

cv : 2 out of 7 done. AM iter = 308

cv : 3 out of 7 done. AM iter = 177

cv : 4 out of 7 done. AM iter = 130

cv : 5 out of 7 done. AM iter = 55

cv : 6 out of 7 done. AM iter = 26

cv : 7 out of 7 done. AM iter = 11

model id done, even death, mu = lambda, lambda* = 0.005. AM iter = 24

BEZ BOOT iter 72 done out of 120

cv : 1 out of 7 done. AM iter = 90

cv : 2 out of 7 done. AM iter = 89

cv : 3 out of 7 done. AM iter = 85

cv : 4 out of 7 done. AM iter = 82

cv : 5 out of 7 done. AM iter = 36

cv : 6 out of 7 done. AM iter = 30

cv : 7 out of 7 done. AM iter = 12

model id done, even death, mu = lambda, lambda* = 0.01. AM iter = 46

BEZ BOOT iter 73 done out of 120

cv : 1 out of 7 done. AM iter = 79

cv : 2 out of 7 done. AM iter = 77

cv : 3 out of 7 done. AM iter = 74

cv : 4 out of 7 done. AM iter = 75

cv : 5 out of 7 done. AM iter = 64

cv : 6 out of 7 done. AM iter = 45

cv : 7 out of 7 done. AM iter = 13

model id done, even death, mu = lambda, lambda* = 0.005. AM iter = 50

BEZ BOOT iter 74 done out of 120

cv : 1 out of 7 done. AM iter = 50

cv : 2 out of 7 done. AM iter = 53

cv : 3 out of 7 done. AM iter = 59

cv : 4 out of 7 done. AM iter = 60

cv : 5 out of 7 done. AM iter = 43

cv : 6 out of 7 done. AM iter = 22

cv : 7 out of 7 done. AM iter = 11

model id done, even death, mu = lambda, lambda* = 0.01. AM iter = 50

BEZ BOOT iter 75 done out of 120

cv : 1 out of 7 done. AM iter = 107

cv : 2 out of 7 done. AM iter = 108

cv : 3 out of 7 done. AM iter = 123

cv : 4 out of 7 done. AM iter = 177

cv : 5 out of 7 done. AM iter = 59

cv : 6 out of 7 done. AM iter = 31

cv : 7 out of 7 done. AM iter = 12

model id done, even death, mu = lambda, lambda* = 0.01. AM iter = 49

BEZ BOOT iter 76 done out of 120

cv : 1 out of 7 done. AM iter = 106

cv : 2 out of 7 done. AM iter = 106

cv : 3 out of 7 done. AM iter = 106

cv : 4 out of 7 done. AM iter = 147

cv : 5 out of 7 done. AM iter = 68

cv : 6 out of 7 done. AM iter = 39

cv : 7 out of 7 done. AM iter = 13

model id done, even death, mu = lambda, lambda* = 0.005. AM iter = 37

BEZ BOOT iter 77 done out of 120

cv : 1 out of 7 done. AM iter = 735

cv : 2 out of 7 done. AM iter = 164

cv : 3 out of 7 done. AM iter = 153

cv : 4 out of 7 done. AM iter = 254

cv : 5 out of 7 done. AM iter = 65

cv : 6 out of 7 done. AM iter = 53

cv : 7 out of 7 done. AM iter = 13

model id done, even death, mu = lambda, lambda* = 0.01. AM iter = 71

BEZ BOOT iter 78 done out of 120

cv : 1 out of 7 done. AM iter = 56

cv : 2 out of 7 done. AM iter = 56

cv : 3 out of 7 done. AM iter = 231

cv : 4 out of 7 done. AM iter = 200

cv : 5 out of 7 done. AM iter = 66

cv : 6 out of 7 done. AM iter = 37

cv : 7 out of 7 done. AM iter = 12

model id done, even death, mu = lambda, lambda* = 0.005. AM iter = 54

BEZ BOOT iter 79 done out of 120

cv : 1 out of 7 done. AM iter = 1000

cv : 2 out of 7 done. AM iter = 948

cv : 3 out of 7 done. AM iter = 247

cv : 4 out of 7 done. AM iter = 158

cv : 5 out of 7 done. AM iter = 69

cv : 6 out of 7 done. AM iter = 50

cv : 7 out of 7 done. AM iter = 12

model id done, even death, mu = lambda, lambda* = 0.005. AM iter = 41

BEZ BOOT iter 80 done out of 120

cv : 1 out of 7 done. AM iter = 85

cv : 2 out of 7 done. AM iter = 85

cv : 3 out of 7 done. AM iter = 161

cv : 4 out of 7 done. AM iter = 105

cv : 5 out of 7 done. AM iter = 40

cv : 6 out of 7 done. AM iter = 28

cv : 7 out of 7 done. AM iter = 12

model id done, even death, mu = lambda, lambda* = 0.01. AM iter = 48

BEZ BOOT iter 81 done out of 120

cv : 1 out of 7 done. AM iter = 157

cv : 2 out of 7 done. AM iter = 152

cv : 3 out of 7 done. AM iter = 125

cv : 4 out of 7 done. AM iter = 105

cv : 5 out of 7 done. AM iter = 41

cv : 6 out of 7 done. AM iter = 29

cv : 7 out of 7 done. AM iter = 12

model id done, even death, mu = lambda, lambda* = 0.05. AM iter = 143

BEZ BOOT iter 82 done out of 120

cv : 1 out of 7 done. AM iter = 624

cv : 2 out of 7 done. AM iter = 1000

cv : 3 out of 7 done. AM iter = 395

cv : 4 out of 7 done. AM iter = 244

cv : 5 out of 7 done. AM iter = 58

cv : 6 out of 7 done. AM iter = 39

cv : 7 out of 7 done. AM iter = 13

model id done, even death, mu = lambda, lambda* = 0.01. AM iter = 98

BEZ BOOT iter 83 done out of 120

cv : 1 out of 7 done. AM iter = 202

cv : 2 out of 7 done. AM iter = 202

cv : 3 out of 7 done. AM iter = 221

cv : 4 out of 7 done. AM iter = 197

cv : 5 out of 7 done. AM iter = 38

cv : 6 out of 7 done. AM iter = 27

cv : 7 out of 7 done. AM iter = 11

model id done, even death, mu = lambda, lambda* = 0.01. AM iter = 52

BEZ BOOT iter 84 done out of 120

cv : 1 out of 7 done. AM iter = 83

cv : 2 out of 7 done. AM iter = 83

cv : 3 out of 7 done. AM iter = 80

cv : 4 out of 7 done. AM iter = 77

cv : 5 out of 7 done. AM iter = 26

cv : 6 out of 7 done. AM iter = 19

cv : 7 out of 7 done. AM iter = 10

model id done, even death, mu = lambda, lambda* = 0.01. AM iter = 34

BEZ BOOT iter 85 done out of 120

cv : 1 out of 7 done. AM iter = 74

cv : 2 out of 7 done. AM iter = 74

cv : 3 out of 7 done. AM iter = 70

cv : 4 out of 7 done. AM iter = 66

cv : 5 out of 7 done. AM iter = 27

cv : 6 out of 7 done. AM iter = 21

cv : 7 out of 7 done. AM iter = 11

model id done, even death, mu = lambda, lambda* = 0.01. AM iter = 33

BEZ BOOT iter 86 done out of 120

cv : 1 out of 7 done. AM iter = 73

cv : 2 out of 7 done. AM iter = 72

cv : 3 out of 7 done. AM iter = 69

cv : 4 out of 7 done. AM iter = 155

cv : 5 out of 7 done. AM iter = 38

cv : 6 out of 7 done. AM iter = 25

cv : 7 out of 7 done. AM iter = 10

model id done, even death, mu = lambda, lambda* = 0.01. AM iter = 43

BEZ BOOT iter 87 done out of 120

cv : 1 out of 7 done. AM iter = 91

cv : 2 out of 7 done. AM iter = 91

cv : 3 out of 7 done. AM iter = 94

cv : 4 out of 7 done. AM iter = 104

cv : 5 out of 7 done. AM iter = 43

cv : 6 out of 7 done. AM iter = 29

cv : 7 out of 7 done. AM iter = 12

model id done, even death, mu = lambda, lambda* = 0.01. AM iter = 42

BEZ BOOT iter 88 done out of 120

cv : 1 out of 7 done. AM iter = 65

cv : 2 out of 7 done. AM iter = 65

cv : 3 out of 7 done. AM iter = 147

cv : 4 out of 7 done. AM iter = 148

cv : 5 out of 7 done. AM iter = 53

cv : 6 out of 7 done. AM iter = 42

cv : 7 out of 7 done. AM iter = 12

model id done, even death, mu = lambda, lambda* = 0.01. AM iter = 91

BEZ BOOT iter 89 done out of 120

cv : 1 out of 7 done. AM iter = 53

cv : 2 out of 7 done. AM iter = 63

cv : 3 out of 7 done. AM iter = 48

cv : 4 out of 7 done. AM iter = 37

cv : 5 out of 7 done. AM iter = 26

cv : 6 out of 7 done. AM iter = 17

cv : 7 out of 7 done. AM iter = 10

model id done, even death, mu = lambda, lambda* = 0.01. AM iter = 36

BEZ BOOT iter 90 done out of 120

cv : 1 out of 7 done. AM iter = 66

cv : 2 out of 7 done. AM iter = 65

cv : 3 out of 7 done. AM iter = 63

cv : 4 out of 7 done. AM iter = 87

cv : 5 out of 7 done. AM iter = 41

cv : 6 out of 7 done. AM iter = 26

cv : 7 out of 7 done. AM iter = 10

model id done, even death, mu = lambda, lambda* = 0.005. AM iter = 32

BEZ BOOT iter 91 done out of 120

cv : 1 out of 7 done. AM iter = 48

cv : 2 out of 7 done. AM iter = 49

cv : 3 out of 7 done. AM iter = 52

cv : 4 out of 7 done. AM iter = 48

cv : 5 out of 7 done. AM iter = 45

cv : 6 out of 7 done. AM iter = 42

cv : 7 out of 7 done. AM iter = 11

model id done, even death, mu = lambda, lambda* = 0.01. AM iter = 48

BEZ BOOT iter 92 done out of 120

cv : 1 out of 7 done. AM iter = 96

cv : 2 out of 7 done. AM iter = 95

cv : 3 out of 7 done. AM iter = 78

cv : 4 out of 7 done. AM iter = 83

cv : 5 out of 7 done. AM iter = 40

cv : 6 out of 7 done. AM iter = 33

cv : 7 out of 7 done. AM iter = 13

model id done, even death, mu = lambda, lambda* = 0.01. AM iter = 51

BEZ BOOT iter 93 done out of 120

cv : 1 out of 7 done. AM iter = 138

cv : 2 out of 7 done. AM iter = 141

cv : 3 out of 7 done. AM iter = 183

cv : 4 out of 7 done. AM iter = 61

cv : 5 out of 7 done. AM iter = 37

cv : 6 out of 7 done. AM iter = 24

cv : 7 out of 7 done. AM iter = 12

model id done, even death, mu = lambda, lambda* = 0.01. AM iter = 38

BEZ BOOT iter 94 done out of 120

cv : 1 out of 7 done. AM iter = 129

cv : 2 out of 7 done. AM iter = 118

cv : 3 out of 7 done. AM iter = 107

cv : 4 out of 7 done. AM iter = 47

cv : 5 out of 7 done. AM iter = 35

cv : 6 out of 7 done. AM iter = 23

cv : 7 out of 7 done. AM iter = 13

model id done, even death, mu = lambda, lambda* = 0.005. AM iter = 37

BEZ BOOT iter 95 done out of 120

cv : 1 out of 7 done. AM iter = 95

cv : 2 out of 7 done. AM iter = 93

cv : 3 out of 7 done. AM iter = 96

cv : 4 out of 7 done. AM iter = 93

cv : 5 out of 7 done. AM iter = 36

cv : 6 out of 7 done. AM iter = 25

cv : 7 out of 7 done. AM iter = 11

model id done, even death, mu = lambda, lambda* = 0.01. AM iter = 43

BEZ BOOT iter 96 done out of 120

cv : 1 out of 7 done. AM iter = 259

cv : 2 out of 7 done. AM iter = 259

cv : 3 out of 7 done. AM iter = 365

cv : 4 out of 7 done. AM iter = 263

cv : 5 out of 7 done. AM iter = 59

cv : 6 out of 7 done. AM iter = 36

cv : 7 out of 7 done. AM iter = 11

model id done, even death, mu = lambda, lambda* = 0.01. AM iter = 113

BEZ BOOT iter 97 done out of 120

cv : 1 out of 7 done. AM iter = 95

cv : 2 out of 7 done. AM iter = 94

cv : 3 out of 7 done. AM iter = 86

cv : 4 out of 7 done. AM iter = 75

cv : 5 out of 7 done. AM iter = 51

cv : 6 out of 7 done. AM iter = 30

cv : 7 out of 7 done. AM iter = 12

model id done, even death, mu = lambda, lambda* = 0.005. AM iter = 37

BEZ BOOT iter 98 done out of 120

cv : 1 out of 7 done. AM iter = 122

cv : 2 out of 7 done. AM iter = 126

cv : 3 out of 7 done. AM iter = 366

cv : 4 out of 7 done. AM iter = 163

cv : 5 out of 7 done. AM iter = 72

cv : 6 out of 7 done. AM iter = 55

cv : 7 out of 7 done. AM iter = 15

model id done, even death, mu = lambda, lambda* = 0.01. AM iter = 79

BEZ BOOT iter 99 done out of 120

cv : 1 out of 7 done. AM iter = 163

cv : 2 out of 7 done. AM iter = 161

cv : 3 out of 7 done. AM iter = 106

cv : 4 out of 7 done. AM iter = 146

cv : 5 out of 7 done. AM iter = 64

cv : 6 out of 7 done. AM iter = 49

cv : 7 out of 7 done. AM iter = 13

model id done, even death, mu = lambda, lambda* = 0.01. AM iter = 53

BEZ BOOT iter 100 done out of 120

cv : 1 out of 7 done. AM iter = 122

cv : 2 out of 7 done. AM iter = 121

cv : 3 out of 7 done. AM iter = 115

cv : 4 out of 7 done. AM iter = 127

cv : 5 out of 7 done. AM iter = 61

cv : 6 out of 7 done. AM iter = 45

cv : 7 out of 7 done. AM iter = 12

model id done, even death, mu = lambda, lambda* = 0.01. AM iter = 64

BEZ BOOT iter 101 done out of 120

cv : 1 out of 7 done. AM iter = 138

cv : 2 out of 7 done. AM iter = 143

cv : 3 out of 7 done. AM iter = 113

cv : 4 out of 7 done. AM iter = 117

cv : 5 out of 7 done. AM iter = 50

cv : 6 out of 7 done. AM iter = 43

cv : 7 out of 7 done. AM iter = 12

model id done, even death, mu = lambda, lambda* = 0.01. AM iter = 45

BEZ BOOT iter 102 done out of 120

cv : 1 out of 7 done. AM iter = 111

cv : 2 out of 7 done. AM iter = 110

cv : 3 out of 7 done. AM iter = 102

cv : 4 out of 7 done. AM iter = 118

cv : 5 out of 7 done. AM iter = 43

cv : 6 out of 7 done. AM iter = 35

cv : 7 out of 7 done. AM iter = 12

model id done, even death, mu = lambda, lambda* = 0.01. AM iter = 64

BEZ BOOT iter 103 done out of 120

cv : 1 out of 7 done. AM iter = 74

cv : 2 out of 7 done. AM iter = 68

cv : 3 out of 7 done. AM iter = 55

cv : 4 out of 7 done. AM iter = 64

cv : 5 out of 7 done. AM iter = 51

cv : 6 out of 7 done. AM iter = 37

cv : 7 out of 7 done. AM iter = 12

model id done, even death, mu = lambda, lambda* = 0.01. AM iter = 50

BEZ BOOT iter 104 done out of 120

cv : 1 out of 7 done. AM iter = 48

cv : 2 out of 7 done. AM iter = 48

cv : 3 out of 7 done. AM iter = 122

cv : 4 out of 7 done. AM iter = 105

cv : 5 out of 7 done. AM iter = 46

cv : 6 out of 7 done. AM iter = 31

cv : 7 out of 7 done. AM iter = 11

model id done, even death, mu = lambda, lambda* = 0.05. AM iter = 143

BEZ BOOT iter 105 done out of 120

cv : 1 out of 7 done. AM iter = 97

cv : 2 out of 7 done. AM iter = 95

cv : 3 out of 7 done. AM iter = 80

cv : 4 out of 7 done. AM iter = 202

cv : 5 out of 7 done. AM iter = 49

cv : 6 out of 7 done. AM iter = 27

cv : 7 out of 7 done. AM iter = 12

model id done, even death, mu = lambda, lambda* = 0.05. AM iter = 157

BEZ BOOT iter 106 done out of 120

cv : 1 out of 7 done. AM iter = 158

cv : 2 out of 7 done. AM iter = 151

cv : 3 out of 7 done. AM iter = 115

cv : 4 out of 7 done. AM iter = 220

cv : 5 out of 7 done. AM iter = 44

cv : 6 out of 7 done. AM iter = 28

cv : 7 out of 7 done. AM iter = 12

model id done, even death, mu = lambda, lambda* = 1. AM iter = 261

BEZ BOOT iter 107 done out of 120

cv : 1 out of 7 done. AM iter = 49

cv : 2 out of 7 done. AM iter = 49

cv : 3 out of 7 done. AM iter = 41

cv : 4 out of 7 done. AM iter = 39

cv : 5 out of 7 done. AM iter = 32

cv : 6 out of 7 done. AM iter = 25

cv : 7 out of 7 done. AM iter = 11

model id done, even death, mu = lambda, lambda* = 0.005. AM iter = 40

BEZ BOOT iter 108 done out of 120

cv : 1 out of 7 done. AM iter = 114

cv : 2 out of 7 done. AM iter = 114

cv : 3 out of 7 done. AM iter = 89

cv : 4 out of 7 done. AM iter = 86

cv : 5 out of 7 done. AM iter = 50

cv : 6 out of 7 done. AM iter = 28

cv : 7 out of 7 done. AM iter = 10

model id done, even death, mu = lambda, lambda* = 0.005. AM iter = 40

BEZ BOOT iter 109 done out of 120

cv : 1 out of 7 done. AM iter = 252

cv : 2 out of 7 done. AM iter = 250

cv : 3 out of 7 done. AM iter = 245

cv : 4 out of 7 done. AM iter = 470

cv : 5 out of 7 done. AM iter = 57

cv : 6 out of 7 done. AM iter = 44

cv : 7 out of 7 done. AM iter = 13

model id done, even death, mu = lambda, lambda* = 0.01. AM iter = 93

BEZ BOOT iter 110 done out of 120

cv : 1 out of 7 done. AM iter = 74

cv : 2 out of 7 done. AM iter = 73

cv : 3 out of 7 done. AM iter = 73

cv : 4 out of 7 done. AM iter = 73

cv : 5 out of 7 done. AM iter = 43

cv : 6 out of 7 done. AM iter = 30

cv : 7 out of 7 done. AM iter = 11

model id done, even death, mu = lambda, lambda* = 0.01. AM iter = 50

BEZ BOOT iter 111 done out of 120

cv : 1 out of 7 done. AM iter = 70

cv : 2 out of 7 done. AM iter = 69

cv : 3 out of 7 done. AM iter = 63

cv : 4 out of 7 done. AM iter = 58

cv : 5 out of 7 done. AM iter = 36

cv : 6 out of 7 done. AM iter = 26

cv : 7 out of 7 done. AM iter = 12

model id done, even death, mu = lambda, lambda* = 0.01. AM iter = 38

BEZ BOOT iter 112 done out of 120

cv : 1 out of 7 done. AM iter = 131

cv : 2 out of 7 done. AM iter = 128

cv : 3 out of 7 done. AM iter = 100

cv : 4 out of 7 done. AM iter = 89

cv : 5 out of 7 done. AM iter = 46

cv : 6 out of 7 done. AM iter = 25

cv : 7 out of 7 done. AM iter = 11

model id done, even death, mu = lambda, lambda* = 0.01. AM iter = 58

BEZ BOOT iter 113 done out of 120

cv : 1 out of 7 done. AM iter = 201

cv : 2 out of 7 done. AM iter = 194

cv : 3 out of 7 done. AM iter = 165

cv : 4 out of 7 done. AM iter = 126

cv : 5 out of 7 done. AM iter = 50

cv : 6 out of 7 done. AM iter = 40

cv : 7 out of 7 done. AM iter = 13

model id done, even death, mu = lambda, lambda* = 0.01. AM iter = 41

BEZ BOOT iter 114 done out of 120

cv : 1 out of 7 done. AM iter = 118

cv : 2 out of 7 done. AM iter = 117

cv : 3 out of 7 done. AM iter = 109

cv : 4 out of 7 done. AM iter = 101

cv : 5 out of 7 done. AM iter = 44

cv : 6 out of 7 done. AM iter = 41

cv : 7 out of 7 done. AM iter = 12

model id done, even death, mu = lambda, lambda* = 0.005. AM iter = 44

BEZ BOOT iter 115 done out of 120

cv : 1 out of 7 done. AM iter = 113

cv : 2 out of 7 done. AM iter = 113

cv : 3 out of 7 done. AM iter = 116

cv : 4 out of 7 done. AM iter = 127

cv : 5 out of 7 done. AM iter = 53

cv : 6 out of 7 done. AM iter = 43

cv : 7 out of 7 done. AM iter = 12

model id done, even death, mu = lambda, lambda* = 0.05. AM iter = 132

BEZ BOOT iter 116 done out of 120

cv : 1 out of 7 done. AM iter = 389

cv : 2 out of 7 done. AM iter = 429

cv : 3 out of 7 done. AM iter = 84

cv : 4 out of 7 done. AM iter = 122

cv : 5 out of 7 done. AM iter = 47

cv : 6 out of 7 done. AM iter = 33

cv : 7 out of 7 done. AM iter = 11

model id done, even death, mu = lambda, lambda* = 0.005. AM iter = 66

BEZ BOOT iter 117 done out of 120

cv : 1 out of 7 done. AM iter = 228

cv : 2 out of 7 done. AM iter = 215

cv : 3 out of 7 done. AM iter = 152

cv : 4 out of 7 done. AM iter = 104

cv : 5 out of 7 done. AM iter = 48

cv : 6 out of 7 done. AM iter = 25

cv : 7 out of 7 done. AM iter = 12

model id done, even death, mu = lambda, lambda* = 0.005. AM iter = 46

BEZ BOOT iter 118 done out of 120

cv : 1 out of 7 done. AM iter = 279

cv : 2 out of 7 done. AM iter = 199

cv : 3 out of 7 done. AM iter = 137

cv : 4 out of 7 done. AM iter = 152

cv : 5 out of 7 done. AM iter = 44

cv : 6 out of 7 done. AM iter = 30

cv : 7 out of 7 done. AM iter = 11

model id done, even death, mu = lambda, lambda* = 0.005. AM iter = 40

BEZ BOOT iter 119 done out of 120

cv : 1 out of 7 done. AM iter = 80

cv : 2 out of 7 done. AM iter = 79

cv : 3 out of 7 done. AM iter = 77

cv : 4 out of 7 done. AM iter = 116

cv : 5 out of 7 done. AM iter = 67

cv : 6 out of 7 done. AM iter = 45

cv : 7 out of 7 done. AM iter = 13

model id done, even death, mu = lambda, lambda* = 0.05. AM iter = 132

BEZ BOOT iter 120 done out of 120

cv : 1 out of 7 done. AM iter = 60

cv : 2 out of 7 done. AM iter = 60

cv : 3 out of 7 done. AM iter = 172

cv : 4 out of 7 done. AM iter = 108

cv : 5 out of 7 done. AM iter = 39

cv : 6 out of 7 done. AM iter = 26

cv : 7 out of 7 done. AM iter = 14

model id done, even death, mu = lambda, lambda* = 0.05. AM iter = 48

Comb. BOOT iter 1 done out of 120

cv : 1 out of 7 done. AM iter = 72

cv : 2 out of 7 done. AM iter = 74

cv : 3 out of 7 done. AM iter = 51

cv : 4 out of 7 done. AM iter = 32

cv : 5 out of 7 done. AM iter = 30

cv : 6 out of 7 done. AM iter = 23

cv : 7 out of 7 done. AM iter = 15

model id done, even death, mu = lambda, lambda* = 0.1. AM iter = 74

Comb. BOOT iter 2 done out of 120

cv : 1 out of 7 done. AM iter = 519

cv : 2 out of 7 done. AM iter = 370

cv : 3 out of 7 done. AM iter = 174

cv : 4 out of 7 done. AM iter = 125

cv : 5 out of 7 done. AM iter = 48

cv : 6 out of 7 done. AM iter = 33

cv : 7 out of 7 done. AM iter = 17

model id done, even death, mu = lambda, lambda* = 0.05. AM iter = 56

Comb. BOOT iter 3 done out of 120

cv : 1 out of 7 done. AM iter = 54

cv : 2 out of 7 done. AM iter = 68

cv : 3 out of 7 done. AM iter = 68

cv : 4 out of 7 done. AM iter = 52

cv : 5 out of 7 done. AM iter = 31

cv : 6 out of 7 done. AM iter = 22

cv : 7 out of 7 done. AM iter = 16

model id done, even death, mu = lambda, lambda* = 0.1. AM iter = 36

Comb. BOOT iter 4 done out of 120

cv : 1 out of 7 done. AM iter = 49

cv : 2 out of 7 done. AM iter = 43

cv : 3 out of 7 done. AM iter = 138

cv : 4 out of 7 done. AM iter = 101

cv : 5 out of 7 done. AM iter = 39

cv : 6 out of 7 done. AM iter = 27

cv : 7 out of 7 done. AM iter = 15

model id done, even death, mu = lambda, lambda* = 0.05. AM iter = 49

Comb. BOOT iter 5 done out of 120

cv : 1 out of 7 done. AM iter = 115

cv : 2 out of 7 done. AM iter = 101

cv : 3 out of 7 done. AM iter = 59

cv : 4 out of 7 done. AM iter = 57

cv : 5 out of 7 done. AM iter = 37

cv : 6 out of 7 done. AM iter = 28

cv : 7 out of 7 done. AM iter = 15

model id done, even death, mu = lambda, lambda* = 0.1. AM iter = 35

Comb. BOOT iter 6 done out of 120

cv : 1 out of 7 done. AM iter = 352

cv : 2 out of 7 done. AM iter = 419

cv : 3 out of 7 done. AM iter = 251

cv : 4 out of 7 done. AM iter = 130

cv : 5 out of 7 done. AM iter = 41

cv : 6 out of 7 done. AM iter = 27

cv : 7 out of 7 done. AM iter = 15

model id done, even death, mu = lambda, lambda* = 0.01. AM iter = 29

Comb. BOOT iter 7 done out of 120

cv : 1 out of 7 done. AM iter = 36

cv : 2 out of 7 done. AM iter = 38

cv : 3 out of 7 done. AM iter = 51

cv : 4 out of 7 done. AM iter = 45

cv : 5 out of 7 done. AM iter = 31

cv : 6 out of 7 done. AM iter = 26

cv : 7 out of 7 done. AM iter = 15

model id done, even death, mu = lambda, lambda* = 0.1. AM iter = 85

Comb. BOOT iter 8 done out of 120

cv : 1 out of 7 done. AM iter = 168

cv : 2 out of 7 done. AM iter = 144

cv : 3 out of 7 done. AM iter = 87

cv : 4 out of 7 done. AM iter = 98

cv : 5 out of 7 done. AM iter = 42

cv : 6 out of 7 done. AM iter = 31

cv : 7 out of 7 done. AM iter = 16

model id done, even death, mu = lambda, lambda* = 0.01. AM iter = 26

Comb. BOOT iter 9 done out of 120

cv : 1 out of 7 done. AM iter = 98

cv : 2 out of 7 done. AM iter = 128

cv : 3 out of 7 done. AM iter = 67

cv : 4 out of 7 done. AM iter = 75

cv : 5 out of 7 done. AM iter = 32

cv : 6 out of 7 done. AM iter = 26

cv : 7 out of 7 done. AM iter = 15

model id done, even death, mu = lambda, lambda* = 0.05. AM iter = 46

Comb. BOOT iter 10 done out of 120

cv : 1 out of 7 done. AM iter = 134

cv : 2 out of 7 done. AM iter = 133

cv : 3 out of 7 done. AM iter = 112

cv : 4 out of 7 done. AM iter = 79

cv : 5 out of 7 done. AM iter = 43

cv : 6 out of 7 done. AM iter = 30

cv : 7 out of 7 done. AM iter = 16

model id done, even death, mu = lambda, lambda* = 0.01. AM iter = 31

Comb. BOOT iter 11 done out of 120

cv : 1 out of 7 done. AM iter = 33

cv : 2 out of 7 done. AM iter = 32

cv : 3 out of 7 done. AM iter = 39

cv : 4 out of 7 done. AM iter = 61

cv : 5 out of 7 done. AM iter = 34

cv : 6 out of 7 done. AM iter = 25

cv : 7 out of 7 done. AM iter = 15

model id done, even death, mu = lambda, lambda* = 0.05. AM iter = 33

Comb. BOOT iter 12 done out of 120

cv : 1 out of 7 done. AM iter = 34

cv : 2 out of 7 done. AM iter = 36

cv : 3 out of 7 done. AM iter = 98

cv : 4 out of 7 done. AM iter = 48

cv : 5 out of 7 done. AM iter = 32

cv : 6 out of 7 done. AM iter = 25

cv : 7 out of 7 done. AM iter = 16

model id done, even death, mu = lambda, lambda* = 0.05. AM iter = 34

Comb. BOOT iter 13 done out of 120

cv : 1 out of 7 done. AM iter = 88

cv : 2 out of 7 done. AM iter = 87

cv : 3 out of 7 done. AM iter = 58

cv : 4 out of 7 done. AM iter = 53

cv : 5 out of 7 done. AM iter = 40

cv : 6 out of 7 done. AM iter = 27

cv : 7 out of 7 done. AM iter = 17

model id done, even death, mu = lambda, lambda* = 0.05. AM iter = 40

Comb. BOOT iter 14 done out of 120

cv : 1 out of 7 done. AM iter = 47

cv : 2 out of 7 done. AM iter = 72

cv : 3 out of 7 done. AM iter = 74

cv : 4 out of 7 done. AM iter = 63

cv : 5 out of 7 done. AM iter = 34

cv : 6 out of 7 done. AM iter = 27

cv : 7 out of 7 done. AM iter = 16

model id done, even death, mu = lambda, lambda* = 0.01. AM iter = 25

Comb. BOOT iter 15 done out of 120

cv : 1 out of 7 done. AM iter = 99

cv : 2 out of 7 done. AM iter = 167

cv : 3 out of 7 done. AM iter = 95

cv : 4 out of 7 done. AM iter = 48

cv : 5 out of 7 done. AM iter = 35

cv : 6 out of 7 done. AM iter = 26

cv : 7 out of 7 done. AM iter = 16

model id done, even death, mu = lambda, lambda* = 0.01. AM iter = 26

Comb. BOOT iter 16 done out of 120

cv : 1 out of 7 done. AM iter = 28

cv : 2 out of 7 done. AM iter = 29

cv : 3 out of 7 done. AM iter = 43

cv : 4 out of 7 done. AM iter = 54

cv : 5 out of 7 done. AM iter = 32

cv : 6 out of 7 done. AM iter = 25

cv : 7 out of 7 done. AM iter = 16

model id done, even death, mu = lambda, lambda* = 0.05. AM iter = 41

Comb. BOOT iter 17 done out of 120

cv : 1 out of 7 done. AM iter = 141

cv : 2 out of 7 done. AM iter = 99

cv : 3 out of 7 done. AM iter = 44

cv : 4 out of 7 done. AM iter = 42

cv : 5 out of 7 done. AM iter = 27

cv : 6 out of 7 done. AM iter = 22

cv : 7 out of 7 done. AM iter = 16

model id done, even death, mu = lambda, lambda* = 0.01. AM iter = 21

Comb. BOOT iter 18 done out of 120

cv : 1 out of 7 done. AM iter = 102

cv : 2 out of 7 done. AM iter = 97

cv : 3 out of 7 done. AM iter = 71

cv : 4 out of 7 done. AM iter = 44

cv : 5 out of 7 done. AM iter = 33

cv : 6 out of 7 done. AM iter = 25

cv : 7 out of 7 done. AM iter = 16

model id done, even death, mu = lambda, lambda* = 0.05. AM iter = 26

Comb. BOOT iter 19 done out of 120

cv : 1 out of 7 done. AM iter = 96

cv : 2 out of 7 done. AM iter = 120

cv : 3 out of 7 done. AM iter = 72

cv : 4 out of 7 done. AM iter = 49

cv : 5 out of 7 done. AM iter = 36

cv : 6 out of 7 done. AM iter = 21

cv : 7 out of 7 done. AM iter = 17

model id done, even death, mu = lambda, lambda* = 0.05. AM iter = 64

Comb. BOOT iter 20 done out of 120

cv : 1 out of 7 done. AM iter = 103

cv : 2 out of 7 done. AM iter = 115

cv : 3 out of 7 done. AM iter = 161

cv : 4 out of 7 done. AM iter = 73

cv : 5 out of 7 done. AM iter = 47

cv : 6 out of 7 done. AM iter = 34

cv : 7 out of 7 done. AM iter = 16

model id done, even death, mu = lambda, lambda* = 0.01. AM iter = 26

Comb. BOOT iter 21 done out of 120

cv : 1 out of 7 done. AM iter = 98

cv : 2 out of 7 done. AM iter = 114

cv : 3 out of 7 done. AM iter = 62

cv : 4 out of 7 done. AM iter = 55

cv : 5 out of 7 done. AM iter = 44

cv : 6 out of 7 done. AM iter = 31

cv : 7 out of 7 done. AM iter = 18

model id done, even death, mu = lambda, lambda* = 0.05. AM iter = 34

Comb. BOOT iter 22 done out of 120

cv : 1 out of 7 done. AM iter = 66

cv : 2 out of 7 done. AM iter = 67

cv : 3 out of 7 done. AM iter = 66

cv : 4 out of 7 done. AM iter = 61

cv : 5 out of 7 done. AM iter = 40

cv : 6 out of 7 done. AM iter = 30

cv : 7 out of 7 done. AM iter = 18

model id done, even death, mu = lambda, lambda* = 0.05. AM iter = 33

Comb. BOOT iter 23 done out of 120

cv : 1 out of 7 done. AM iter = 100

cv : 2 out of 7 done. AM iter = 90

cv : 3 out of 7 done. AM iter = 58

cv : 4 out of 7 done. AM iter = 65

cv : 5 out of 7 done. AM iter = 28

cv : 6 out of 7 done. AM iter = 22

cv : 7 out of 7 done. AM iter = 14

model id done, even death, mu = lambda, lambda* = 0.01. AM iter = 21

Comb. BOOT iter 24 done out of 120

cv : 1 out of 7 done. AM iter = 63

cv : 2 out of 7 done. AM iter = 66

cv : 3 out of 7 done. AM iter = 137

cv : 4 out of 7 done. AM iter = 66

cv : 5 out of 7 done. AM iter = 38

cv : 6 out of 7 done. AM iter = 27

cv : 7 out of 7 done. AM iter = 17

model id done, even death, mu = lambda, lambda* = 0.05. AM iter = 49

Comb. BOOT iter 25 done out of 120

cv : 1 out of 7 done. AM iter = 74

cv : 2 out of 7 done. AM iter = 219

cv : 3 out of 7 done. AM iter = 81

cv : 4 out of 7 done. AM iter = 64

cv : 5 out of 7 done. AM iter = 38

cv : 6 out of 7 done. AM iter = 29

cv : 7 out of 7 done. AM iter = 16

model id done, even death, mu = lambda, lambda* = 0.05. AM iter = 47

Comb. BOOT iter 26 done out of 120

cv : 1 out of 7 done. AM iter = 52

cv : 2 out of 7 done. AM iter = 78

cv : 3 out of 7 done. AM iter = 99

cv : 4 out of 7 done. AM iter = 57

cv : 5 out of 7 done. AM iter = 38

cv : 6 out of 7 done. AM iter = 28

cv : 7 out of 7 done. AM iter = 17

model id done, even death, mu = lambda, lambda* = 0.01. AM iter = 27

Comb. BOOT iter 27 done out of 120

cv : 1 out of 7 done. AM iter = 53

cv : 2 out of 7 done. AM iter = 53

cv : 3 out of 7 done. AM iter = 76

cv : 4 out of 7 done. AM iter = 68

cv : 5 out of 7 done. AM iter = 43

cv : 6 out of 7 done. AM iter = 30

cv : 7 out of 7 done. AM iter = 16

model id done, even death, mu = lambda, lambda* = 0.01. AM iter = 32

Comb. BOOT iter 28 done out of 120

cv : 1 out of 7 done. AM iter = 53

cv : 2 out of 7 done. AM iter = 56

cv : 3 out of 7 done. AM iter = 187

cv : 4 out of 7 done. AM iter = 83

cv : 5 out of 7 done. AM iter = 35

cv : 6 out of 7 done. AM iter = 27

cv : 7 out of 7 done. AM iter = 16

model id done, even death, mu = lambda, lambda* = 0.05. AM iter = 53

Comb. BOOT iter 29 done out of 120

cv : 1 out of 7 done. AM iter = 79

cv : 2 out of 7 done. AM iter = 79

cv : 3 out of 7 done. AM iter = 106

cv : 4 out of 7 done. AM iter = 74

cv : 5 out of 7 done. AM iter = 50

cv : 6 out of 7 done. AM iter = 33

cv : 7 out of 7 done. AM iter = 16

model id done, even death, mu = lambda, lambda* = 0.01. AM iter = 31

Comb. BOOT iter 30 done out of 120

cv : 1 out of 7 done. AM iter = 467

cv : 2 out of 7 done. AM iter = 135

cv : 3 out of 7 done. AM iter = 116

cv : 4 out of 7 done. AM iter = 108

cv : 5 out of 7 done. AM iter = 48

cv : 6 out of 7 done. AM iter = 34

cv : 7 out of 7 done. AM iter = 17

model id done, even death, mu = lambda, lambda* = 0.05. AM iter = 39

Comb. BOOT iter 31 done out of 120

cv : 1 out of 7 done. AM iter = 120

cv : 2 out of 7 done. AM iter = 115

cv : 3 out of 7 done. AM iter = 64

cv : 4 out of 7 done. AM iter = 53

cv : 5 out of 7 done. AM iter = 33

cv : 6 out of 7 done. AM iter = 26

cv : 7 out of 7 done. AM iter = 16

model id done, even death, mu = lambda, lambda* = 0.01. AM iter = 27

Comb. BOOT iter 32 done out of 120

cv : 1 out of 7 done. AM iter = 52

cv : 2 out of 7 done. AM iter = 54

cv : 3 out of 7 done. AM iter = 88

cv : 4 out of 7 done. AM iter = 62

cv : 5 out of 7 done. AM iter = 46

cv : 6 out of 7 done. AM iter = 31

cv : 7 out of 7 done. AM iter = 16

model id done, even death, mu = lambda, lambda* = 0.01. AM iter = 30

Comb. BOOT iter 33 done out of 120

cv : 1 out of 7 done. AM iter = 80

cv : 2 out of 7 done. AM iter = 98

cv : 3 out of 7 done. AM iter = 103

cv : 4 out of 7 done. AM iter = 79

cv : 5 out of 7 done. AM iter = 35

cv : 6 out of 7 done. AM iter = 26

cv : 7 out of 7 done. AM iter = 15

model id done, even death, mu = lambda, lambda* = 0.01. AM iter = 26

Comb. BOOT iter 34 done out of 120

cv : 1 out of 7 done. AM iter = 121

cv : 2 out of 7 done. AM iter = 115

cv : 3 out of 7 done. AM iter = 84

cv : 4 out of 7 done. AM iter = 113

cv : 5 out of 7 done. AM iter = 35

cv : 6 out of 7 done. AM iter = 25

cv : 7 out of 7 done. AM iter = 15

model id done, even death, mu = lambda, lambda* = 0.01. AM iter = 25

Comb. BOOT iter 35 done out of 120

cv : 1 out of 7 done. AM iter = 70

cv : 2 out of 7 done. AM iter = 67

cv : 3 out of 7 done. AM iter = 54

cv : 4 out of 7 done. AM iter = 35

cv : 5 out of 7 done. AM iter = 32

cv : 6 out of 7 done. AM iter = 24

cv : 7 out of 7 done. AM iter = 15

model id done, even death, mu = lambda, lambda* = 0.01. AM iter = 25

Comb. BOOT iter 36 done out of 120

cv : 1 out of 7 done. AM iter = 85

cv : 2 out of 7 done. AM iter = 85

cv : 3 out of 7 done. AM iter = 117

cv : 4 out of 7 done. AM iter = 88

cv : 5 out of 7 done. AM iter = 36

cv : 6 out of 7 done. AM iter = 27

cv : 7 out of 7 done. AM iter = 16

model id done, even death, mu = lambda, lambda* = 0.1. AM iter = 82

Comb. BOOT iter 37 done out of 120

cv : 1 out of 7 done. AM iter = 100

cv : 2 out of 7 done. AM iter = 209

cv : 3 out of 7 done. AM iter = 103

cv : 4 out of 7 done. AM iter = 103

cv : 5 out of 7 done. AM iter = 47

cv : 6 out of 7 done. AM iter = 32

cv : 7 out of 7 done. AM iter = 16

model id done, even death, mu = lambda, lambda* = 0.05. AM iter = 44

Comb. BOOT iter 38 done out of 120

cv : 1 out of 7 done. AM iter = 113

cv : 2 out of 7 done. AM iter = 113

cv : 3 out of 7 done. AM iter = 90

cv : 4 out of 7 done. AM iter = 82

cv : 5 out of 7 done. AM iter = 46

cv : 6 out of 7 done. AM iter = 32

cv : 7 out of 7 done. AM iter = 17

model id done, even death, mu = lambda, lambda* = 0.01. AM iter = 31

Comb. BOOT iter 39 done out of 120

cv : 1 out of 7 done. AM iter = 76

cv : 2 out of 7 done. AM iter = 76

cv : 3 out of 7 done. AM iter = 107

cv : 4 out of 7 done. AM iter = 329

cv : 5 out of 7 done. AM iter = 67

cv : 6 out of 7 done. AM iter = 42

cv : 7 out of 7 done. AM iter = 18

model id done, even death, mu = lambda, lambda* = 0.05. AM iter = 68

Comb. BOOT iter 40 done out of 120

cv : 1 out of 7 done. AM iter = 184

cv : 2 out of 7 done. AM iter = 161

cv : 3 out of 7 done. AM iter = 83

cv : 4 out of 7 done. AM iter = 57

cv : 5 out of 7 done. AM iter = 38

cv : 6 out of 7 done. AM iter = 26

cv : 7 out of 7 done. AM iter = 14

model id done, even death, mu = lambda, lambda* = 0.01. AM iter = 25

Comb. BOOT iter 41 done out of 120

cv : 1 out of 7 done. AM iter = 92

cv : 2 out of 7 done. AM iter = 128

cv : 3 out of 7 done. AM iter = 97

cv : 4 out of 7 done. AM iter = 77

cv : 5 out of 7 done. AM iter = 40

cv : 6 out of 7 done. AM iter = 29

cv : 7 out of 7 done. AM iter = 16

model id done, even death, mu = lambda, lambda* = 0.05. AM iter = 45

Comb. BOOT iter 42 done out of 120

cv : 1 out of 7 done. AM iter = 53

cv : 2 out of 7 done. AM iter = 55

cv : 3 out of 7 done. AM iter = 83

cv : 4 out of 7 done. AM iter = 72

cv : 5 out of 7 done. AM iter = 33

cv : 6 out of 7 done. AM iter = 26

cv : 7 out of 7 done. AM iter = 16

model id done, even death, mu = lambda, lambda* = 0.01. AM iter = 25

Comb. BOOT iter 43 done out of 120

cv : 1 out of 7 done. AM iter = 103

cv : 2 out of 7 done. AM iter = 107

cv : 3 out of 7 done. AM iter = 114

cv : 4 out of 7 done. AM iter = 79

cv : 5 out of 7 done. AM iter = 39

cv : 6 out of 7 done. AM iter = 30

cv : 7 out of 7 done. AM iter = 18

model id done, even death, mu = lambda, lambda* = 0.05. AM iter = 35

Comb. BOOT iter 44 done out of 120

cv : 1 out of 7 done. AM iter = 94

cv : 2 out of 7 done. AM iter = 90

cv : 3 out of 7 done. AM iter = 289

cv : 4 out of 7 done. AM iter = 88

cv : 5 out of 7 done. AM iter = 32

cv : 6 out of 7 done. AM iter = 24

cv : 7 out of 7 done. AM iter = 15

model id done, even death, mu = lambda, lambda* = 0.05. AM iter = 42

Comb. BOOT iter 45 done out of 120

cv : 1 out of 7 done. AM iter = 43

cv : 2 out of 7 done. AM iter = 44

cv : 3 out of 7 done. AM iter = 71

cv : 4 out of 7 done. AM iter = 67

cv : 5 out of 7 done. AM iter = 32

cv : 6 out of 7 done. AM iter = 24

cv : 7 out of 7 done. AM iter = 14

model id done, even death, mu = lambda, lambda* = 0.01. AM iter = 25

Comb. BOOT iter 46 done out of 120

cv : 1 out of 7 done. AM iter = 67

cv : 2 out of 7 done. AM iter = 89

cv : 3 out of 7 done. AM iter = 79

cv : 4 out of 7 done. AM iter = 55

cv : 5 out of 7 done. AM iter = 38

cv : 6 out of 7 done. AM iter = 31

cv : 7 out of 7 done. AM iter = 16

model id done, even death, mu = lambda, lambda* = 0.01. AM iter = 27

Comb. BOOT iter 47 done out of 120

cv : 1 out of 7 done. AM iter = 80

cv : 2 out of 7 done. AM iter = 105

cv : 3 out of 7 done. AM iter = 85

cv : 4 out of 7 done. AM iter = 61

cv : 5 out of 7 done. AM iter = 46

cv : 6 out of 7 done. AM iter = 33

cv : 7 out of 7 done. AM iter = 19

model id done, even death, mu = lambda, lambda* = 0.05. AM iter = 33

Comb. BOOT iter 48 done out of 120

cv : 1 out of 7 done. AM iter = 128

cv : 2 out of 7 done. AM iter = 136

cv : 3 out of 7 done. AM iter = 234

cv : 4 out of 7 done. AM iter = 512

cv : 5 out of 7 done. AM iter = 45

cv : 6 out of 7 done. AM iter = 30

cv : 7 out of 7 done. AM iter = 16

model id done, even death, mu = lambda, lambda* = 0.01. AM iter = 28

Comb. BOOT iter 49 done out of 120

cv : 1 out of 7 done. AM iter = 135

cv : 2 out of 7 done. AM iter = 141

cv : 3 out of 7 done. AM iter = 96

cv : 4 out of 7 done. AM iter = 75

cv : 5 out of 7 done. AM iter = 36

cv : 6 out of 7 done. AM iter = 25

cv : 7 out of 7 done. AM iter = 14

model id done, even death, mu = lambda, lambda* = 0.05. AM iter = 55

Comb. BOOT iter 50 done out of 120

cv : 1 out of 7 done. AM iter = 105

cv : 2 out of 7 done. AM iter = 202

cv : 3 out of 7 done. AM iter = 149

cv : 4 out of 7 done. AM iter = 153

cv : 5 out of 7 done. AM iter = 42

cv : 6 out of 7 done. AM iter = 29

cv : 7 out of 7 done. AM iter = 16

model id done, even death, mu = lambda, lambda* = 0.05. AM iter = 53

Comb. BOOT iter 51 done out of 120

cv : 1 out of 7 done. AM iter = 110

cv : 2 out of 7 done. AM iter = 241

cv : 3 out of 7 done. AM iter = 84

cv : 4 out of 7 done. AM iter = 77

cv : 5 out of 7 done. AM iter = 42

cv : 6 out of 7 done. AM iter = 32

cv : 7 out of 7 done. AM iter = 16

model id done, even death, mu = lambda, lambda* = 0.05. AM iter = 35

Comb. BOOT iter 52 done out of 120

cv : 1 out of 7 done. AM iter = 40

cv : 2 out of 7 done. AM iter = 39

cv : 3 out of 7 done. AM iter = 35

cv : 4 out of 7 done. AM iter = 35

cv : 5 out of 7 done. AM iter = 25

cv : 6 out of 7 done. AM iter = 21

cv : 7 out of 7 done. AM iter = 14

model id done, even death, mu = lambda, lambda* = 0.05. AM iter = 50

Comb. BOOT iter 53 done out of 120

cv : 1 out of 7 done. AM iter = 108

cv : 2 out of 7 done. AM iter = 147

cv : 3 out of 7 done. AM iter = 58

cv : 4 out of 7 done. AM iter = 31

cv : 5 out of 7 done. AM iter = 27

cv : 6 out of 7 done. AM iter = 22

cv : 7 out of 7 done. AM iter = 15

model id done, even death, mu = lambda, lambda* = 0.01. AM iter = 22

Comb. BOOT iter 54 done out of 120

cv : 1 out of 7 done. AM iter = 111

cv : 2 out of 7 done. AM iter = 107

cv : 3 out of 7 done. AM iter = 95

cv : 4 out of 7 done. AM iter = 128

cv : 5 out of 7 done. AM iter = 40

cv : 6 out of 7 done. AM iter = 33

cv : 7 out of 7 done. AM iter = 15

model id done, even death, mu = lambda, lambda* = 0.05. AM iter = 46

Comb. BOOT iter 55 done out of 120

cv : 1 out of 7 done. AM iter = 77

cv : 2 out of 7 done. AM iter = 76

cv : 3 out of 7 done. AM iter = 64

cv : 4 out of 7 done. AM iter = 88

cv : 5 out of 7 done. AM iter = 37

cv : 6 out of 7 done. AM iter = 26

cv : 7 out of 7 done. AM iter = 14

model id done, even death, mu = lambda, lambda* = 0.01. AM iter = 22

Comb. BOOT iter 56 done out of 120

cv : 1 out of 7 done. AM iter = 557

cv : 2 out of 7 done. AM iter = 783

cv : 3 out of 7 done. AM iter = 166

cv : 4 out of 7 done. AM iter = 108

cv : 5 out of 7 done. AM iter = 42

cv : 6 out of 7 done. AM iter = 28

cv : 7 out of 7 done. AM iter = 17

model id done, even death, mu = lambda, lambda* = 0.01. AM iter = 29

Comb. BOOT iter 57 done out of 120

cv : 1 out of 7 done. AM iter = 37

cv : 2 out of 7 done. AM iter = 39

cv : 3 out of 7 done. AM iter = 79

cv : 4 out of 7 done. AM iter = 70

cv : 5 out of 7 done. AM iter = 42

cv : 6 out of 7 done. AM iter = 29

cv : 7 out of 7 done. AM iter = 17

model id done, even death, mu = lambda, lambda* = 0.05. AM iter = 66

Comb. BOOT iter 58 done out of 120

cv : 1 out of 7 done. AM iter = 103

cv : 2 out of 7 done. AM iter = 104

cv : 3 out of 7 done. AM iter = 95

cv : 4 out of 7 done. AM iter = 75

cv : 5 out of 7 done. AM iter = 50

cv : 6 out of 7 done. AM iter = 34

cv : 7 out of 7 done. AM iter = 20

model id done, even death, mu = lambda, lambda* = 0.05. AM iter = 60

Comb. BOOT iter 59 done out of 120

cv : 1 out of 7 done. AM iter = 78

cv : 2 out of 7 done. AM iter = 83

cv : 3 out of 7 done. AM iter = 58

cv : 4 out of 7 done. AM iter = 52

cv : 5 out of 7 done. AM iter = 42

cv : 6 out of 7 done. AM iter = 31

cv : 7 out of 7 done. AM iter = 16

model id done, even death, mu = lambda, lambda* = 0.01. AM iter = 26

Comb. BOOT iter 60 done out of 120

cv : 1 out of 7 done. AM iter = 73

cv : 2 out of 7 done. AM iter = 73

cv : 3 out of 7 done. AM iter = 72

cv : 4 out of 7 done. AM iter = 89

cv : 5 out of 7 done. AM iter = 49

cv : 6 out of 7 done. AM iter = 32

cv : 7 out of 7 done. AM iter = 17

model id done, even death, mu = lambda, lambda* = 0.01. AM iter = 32

Comb. BOOT iter 61 done out of 120

cv : 1 out of 7 done. AM iter = 110

cv : 2 out of 7 done. AM iter = 102

cv : 3 out of 7 done. AM iter = 90

cv : 4 out of 7 done. AM iter = 83

cv : 5 out of 7 done. AM iter = 49

cv : 6 out of 7 done. AM iter = 28

cv : 7 out of 7 done. AM iter = 14

model id done, even death, mu = lambda, lambda* = 0.01. AM iter = 29

Comb. BOOT iter 62 done out of 120

cv : 1 out of 7 done. AM iter = 56

cv : 2 out of 7 done. AM iter = 65

cv : 3 out of 7 done. AM iter = 49

cv : 4 out of 7 done. AM iter = 33

cv : 5 out of 7 done. AM iter = 30

cv : 6 out of 7 done. AM iter = 24

cv : 7 out of 7 done. AM iter = 15

model id done, even death, mu = lambda, lambda* = 0.1. AM iter = 44

Comb. BOOT iter 63 done out of 120

cv : 1 out of 7 done. AM iter = 111

cv : 2 out of 7 done. AM iter = 107

cv : 3 out of 7 done. AM iter = 82

cv : 4 out of 7 done. AM iter = 67

cv : 5 out of 7 done. AM iter = 31

cv : 6 out of 7 done. AM iter = 23

cv : 7 out of 7 done. AM iter = 14

model id done, even death, mu = lambda, lambda* = 0.01. AM iter = 24

Comb. BOOT iter 64 done out of 120

cv : 1 out of 7 done. AM iter = 77

cv : 2 out of 7 done. AM iter = 72

cv : 3 out of 7 done. AM iter = 68

cv : 4 out of 7 done. AM iter = 66

cv : 5 out of 7 done. AM iter = 34

cv : 6 out of 7 done. AM iter = 24

cv : 7 out of 7 done. AM iter = 14

model id done, even death, mu = lambda, lambda* = 0.01. AM iter = 22

Comb. BOOT iter 65 done out of 120

cv : 1 out of 7 done. AM iter = 167

cv : 2 out of 7 done. AM iter = 164

cv : 3 out of 7 done. AM iter = 103

cv : 4 out of 7 done. AM iter = 78

cv : 5 out of 7 done. AM iter = 42

cv : 6 out of 7 done. AM iter = 30

cv : 7 out of 7 done. AM iter = 15

model id done, even death, mu = lambda, lambda* = 0.01. AM iter = 27

Comb. BOOT iter 66 done out of 120

cv : 1 out of 7 done. AM iter = 103

cv : 2 out of 7 done. AM iter = 98

cv : 3 out of 7 done. AM iter = 79

cv : 4 out of 7 done. AM iter = 80

cv : 5 out of 7 done. AM iter = 40

cv : 6 out of 7 done. AM iter = 27

cv : 7 out of 7 done. AM iter = 14

model id done, even death, mu = lambda, lambda* = 0.05. AM iter = 69

Comb. BOOT iter 67 done out of 120

cv : 1 out of 7 done. AM iter = 60

cv : 2 out of 7 done. AM iter = 68

cv : 3 out of 7 done. AM iter = 57

cv : 4 out of 7 done. AM iter = 44

cv : 5 out of 7 done. AM iter = 36

cv : 6 out of 7 done. AM iter = 27

cv : 7 out of 7 done. AM iter = 15

model id done, even death, mu = lambda, lambda* = 0.05. AM iter = 37

Comb. BOOT iter 68 done out of 120

cv : 1 out of 7 done. AM iter = 57

cv : 2 out of 7 done. AM iter = 136

cv : 3 out of 7 done. AM iter = 85

cv : 4 out of 7 done. AM iter = 48

cv : 5 out of 7 done. AM iter = 29

cv : 6 out of 7 done. AM iter = 22

cv : 7 out of 7 done. AM iter = 14

model id done, even death, mu = lambda, lambda* = 0.05. AM iter = 42

Comb. BOOT iter 69 done out of 120

cv : 1 out of 7 done. AM iter = 159

cv : 2 out of 7 done. AM iter = 142

cv : 3 out of 7 done. AM iter = 80

cv : 4 out of 7 done. AM iter = 54

cv : 5 out of 7 done. AM iter = 37

cv : 6 out of 7 done. AM iter = 25

cv : 7 out of 7 done. AM iter = 16

model id done, even death, mu = lambda, lambda* = 0.05. AM iter = 29

Comb. BOOT iter 70 done out of 120

cv : 1 out of 7 done. AM iter = 135

cv : 2 out of 7 done. AM iter = 130

cv : 3 out of 7 done. AM iter = 96

cv : 4 out of 7 done. AM iter = 90

cv : 5 out of 7 done. AM iter = 41

cv : 6 out of 7 done. AM iter = 29

cv : 7 out of 7 done. AM iter = 17

model id done, even death, mu = lambda, lambda* = 0.01. AM iter = 29

Comb. BOOT iter 71 done out of 120

cv : 1 out of 7 done. AM iter = 47

cv : 2 out of 7 done. AM iter = 48

cv : 3 out of 7 done. AM iter = 69

cv : 4 out of 7 done. AM iter = 61

cv : 5 out of 7 done. AM iter = 34

cv : 6 out of 7 done. AM iter = 26

cv : 7 out of 7 done. AM iter = 15

model id done, even death, mu = lambda, lambda* = 0.1. AM iter = 33

Comb. BOOT iter 72 done out of 120

cv : 1 out of 7 done. AM iter = 125

cv : 2 out of 7 done. AM iter = 124

cv : 3 out of 7 done. AM iter = 117

cv : 4 out of 7 done. AM iter = 100

cv : 5 out of 7 done. AM iter = 51

cv : 6 out of 7 done. AM iter = 35

cv : 7 out of 7 done. AM iter = 18

model id done, even death, mu = lambda, lambda* = 0.01. AM iter = 29

Comb. BOOT iter 73 done out of 120

cv : 1 out of 7 done. AM iter = 82

cv : 2 out of 7 done. AM iter = 77

cv : 3 out of 7 done. AM iter = 82

cv : 4 out of 7 done. AM iter = 82

cv : 5 out of 7 done. AM iter = 42

cv : 6 out of 7 done. AM iter = 29

cv : 7 out of 7 done. AM iter = 16

model id done, even death, mu = lambda, lambda* = 0.05. AM iter = 47

Comb. BOOT iter 74 done out of 120

cv : 1 out of 7 done. AM iter = 71

cv : 2 out of 7 done. AM iter = 71

cv : 3 out of 7 done. AM iter = 150

cv : 4 out of 7 done. AM iter = 135

cv : 5 out of 7 done. AM iter = 50

cv : 6 out of 7 done. AM iter = 34

cv : 7 out of 7 done. AM iter = 17

model id done, even death, mu = lambda, lambda* = 0.05. AM iter = 50

Comb. BOOT iter 75 done out of 120

cv : 1 out of 7 done. AM iter = 129

cv : 2 out of 7 done. AM iter = 120

cv : 3 out of 7 done. AM iter = 68

cv : 4 out of 7 done. AM iter = 40

cv : 5 out of 7 done. AM iter = 35

cv : 6 out of 7 done. AM iter = 27

cv : 7 out of 7 done. AM iter = 16

model id done, even death, mu = lambda, lambda* = 0.01. AM iter = 25

Comb. BOOT iter 76 done out of 120

cv : 1 out of 7 done. AM iter = 44

cv : 2 out of 7 done. AM iter = 58

cv : 3 out of 7 done. AM iter = 163

cv : 4 out of 7 done. AM iter = 79

cv : 5 out of 7 done. AM iter = 38

cv : 6 out of 7 done. AM iter = 29

cv : 7 out of 7 done. AM iter = 16

model id done, even death, mu = lambda, lambda* = 0.05. AM iter = 45

Comb. BOOT iter 77 done out of 120

cv : 1 out of 7 done. AM iter = 61

cv : 2 out of 7 done. AM iter = 63

cv : 3 out of 7 done. AM iter = 85

cv : 4 out of 7 done. AM iter = 98

cv : 5 out of 7 done. AM iter = 35

cv : 6 out of 7 done. AM iter = 26

cv : 7 out of 7 done. AM iter = 16

model id done, even death, mu = lambda, lambda* = 0.5. AM iter = 36

Comb. BOOT iter 78 done out of 120

cv : 1 out of 7 done. AM iter = 52

cv : 2 out of 7 done. AM iter = 54

cv : 3 out of 7 done. AM iter = 48

cv : 4 out of 7 done. AM iter = 43

cv : 5 out of 7 done. AM iter = 34

cv : 6 out of 7 done. AM iter = 27

cv : 7 out of 7 done. AM iter = 16

model id done, even death, mu = lambda, lambda* = 0.01. AM iter = 25

Comb. BOOT iter 79 done out of 120

cv : 1 out of 7 done. AM iter = 135

cv : 2 out of 7 done. AM iter = 120

cv : 3 out of 7 done. AM iter = 107

cv : 4 out of 7 done. AM iter = 56

cv : 5 out of 7 done. AM iter = 38

cv : 6 out of 7 done. AM iter = 28

cv : 7 out of 7 done. AM iter = 17

model id done, even death, mu = lambda, lambda* = 0.05. AM iter = 406

Comb. BOOT iter 80 done out of 120

cv : 1 out of 7 done. AM iter = 252

cv : 2 out of 7 done. AM iter = 221

cv : 3 out of 7 done. AM iter = 120

cv : 4 out of 7 done. AM iter = 73

cv : 5 out of 7 done. AM iter = 41

cv : 6 out of 7 done. AM iter = 30

cv : 7 out of 7 done. AM iter = 17

model id done, even death, mu = lambda, lambda* = 0.01. AM iter = 28

Comb. BOOT iter 81 done out of 120

cv : 1 out of 7 done. AM iter = 41

cv : 2 out of 7 done. AM iter = 46

cv : 3 out of 7 done. AM iter = 110

cv : 4 out of 7 done. AM iter = 40

cv : 5 out of 7 done. AM iter = 27

cv : 6 out of 7 done. AM iter = 20

cv : 7 out of 7 done. AM iter = 14

model id done, even death, mu = lambda, lambda* = 0.01. AM iter = 21

Comb. BOOT iter 82 done out of 120

cv : 1 out of 7 done. AM iter = 54

cv : 2 out of 7 done. AM iter = 55

cv : 3 out of 7 done. AM iter = 71

cv : 4 out of 7 done. AM iter = 75

cv : 5 out of 7 done. AM iter = 36

cv : 6 out of 7 done. AM iter = 27

cv : 7 out of 7 done. AM iter = 18

model id done, even death, mu = lambda, lambda* = 0.05. AM iter = 49

Comb. BOOT iter 83 done out of 120

cv : 1 out of 7 done. AM iter = 184

cv : 2 out of 7 done. AM iter = 173

cv : 3 out of 7 done. AM iter = 146

cv : 4 out of 7 done. AM iter = 105

cv : 5 out of 7 done. AM iter = 56

cv : 6 out of 7 done. AM iter = 38

cv : 7 out of 7 done. AM iter = 18

model id done, even death, mu = lambda, lambda* = 0.01. AM iter = 29

Comb. BOOT iter 84 done out of 120

cv : 1 out of 7 done. AM iter = 47

cv : 2 out of 7 done. AM iter = 48

cv : 3 out of 7 done. AM iter = 47

cv : 4 out of 7 done. AM iter = 33

cv : 5 out of 7 done. AM iter = 31

cv : 6 out of 7 done. AM iter = 23

cv : 7 out of 7 done. AM iter = 15

model id done, even death, mu = lambda, lambda* = 0.05. AM iter = 30

Comb. BOOT iter 85 done out of 120

cv : 1 out of 7 done. AM iter = 82

cv : 2 out of 7 done. AM iter = 82

cv : 3 out of 7 done. AM iter = 80

cv : 4 out of 7 done. AM iter = 84

cv : 5 out of 7 done. AM iter = 39

cv : 6 out of 7 done. AM iter = 26

cv : 7 out of 7 done. AM iter = 14

model id done, even death, mu = lambda, lambda* = 0.01. AM iter = 29

Comb. BOOT iter 86 done out of 120

cv : 1 out of 7 done. AM iter = 115

cv : 2 out of 7 done. AM iter = 112

cv : 3 out of 7 done. AM iter = 91

cv : 4 out of 7 done. AM iter = 115

cv : 5 out of 7 done. AM iter = 34

cv : 6 out of 7 done. AM iter = 24

cv : 7 out of 7 done. AM iter = 14

model id done, even death, mu = lambda, lambda* = 0.01. AM iter = 25

Comb. BOOT iter 87 done out of 120

cv : 1 out of 7 done. AM iter = 85

cv : 2 out of 7 done. AM iter = 85

cv : 3 out of 7 done. AM iter = 82

cv : 4 out of 7 done. AM iter = 79

cv : 5 out of 7 done. AM iter = 45

cv : 6 out of 7 done. AM iter = 32

cv : 7 out of 7 done. AM iter = 17

model id done, even death, mu = lambda, lambda* = 0.01. AM iter = 27

Comb. BOOT iter 88 done out of 120

cv : 1 out of 7 done. AM iter = 75

cv : 2 out of 7 done. AM iter = 91

cv : 3 out of 7 done. AM iter = 61

cv : 4 out of 7 done. AM iter = 52

cv : 5 out of 7 done. AM iter = 39

cv : 6 out of 7 done. AM iter = 30

cv : 7 out of 7 done. AM iter = 18

model id done, even death, mu = lambda, lambda* = 0.01. AM iter = 28

Comb. BOOT iter 89 done out of 120

cv : 1 out of 7 done. AM iter = 53

cv : 2 out of 7 done. AM iter = 55

cv : 3 out of 7 done. AM iter = 88

cv : 4 out of 7 done. AM iter = 65

cv : 5 out of 7 done. AM iter = 29

cv : 6 out of 7 done. AM iter = 23

cv : 7 out of 7 done. AM iter = 15

model id done, even death, mu = lambda, lambda* = 0.01. AM iter = 23

Comb. BOOT iter 90 done out of 120

cv : 1 out of 7 done. AM iter = 71

cv : 2 out of 7 done. AM iter = 72

cv : 3 out of 7 done. AM iter = 94

cv : 4 out of 7 done. AM iter = 85

cv : 5 out of 7 done. AM iter = 46

cv : 6 out of 7 done. AM iter = 31

cv : 7 out of 7 done. AM iter = 17

model id done, even death, mu = lambda, lambda* = 0.01. AM iter = 29

Comb. BOOT iter 91 done out of 120

cv : 1 out of 7 done. AM iter = 103

cv : 2 out of 7 done. AM iter = 88

cv : 3 out of 7 done. AM iter = 81

cv : 4 out of 7 done. AM iter = 71

cv : 5 out of 7 done. AM iter = 36

cv : 6 out of 7 done. AM iter = 28

cv : 7 out of 7 done. AM iter = 18

model id done, even death, mu = lambda, lambda* = 0.05. AM iter = 41

Comb. BOOT iter 92 done out of 120

cv : 1 out of 7 done. AM iter = 100

cv : 2 out of 7 done. AM iter = 125

cv : 3 out of 7 done. AM iter = 154

cv : 4 out of 7 done. AM iter = 123

cv : 5 out of 7 done. AM iter = 45

cv : 6 out of 7 done. AM iter = 30

cv : 7 out of 7 done. AM iter = 16

model id done, even death, mu = lambda, lambda* = 0.01. AM iter = 29

Comb. BOOT iter 93 done out of 120

cv : 1 out of 7 done. AM iter = 129

cv : 2 out of 7 done. AM iter = 125

cv : 3 out of 7 done. AM iter = 87

cv : 4 out of 7 done. AM iter = 73

cv : 5 out of 7 done. AM iter = 40

cv : 6 out of 7 done. AM iter = 29

cv : 7 out of 7 done. AM iter = 16

model id done, even death, mu = lambda, lambda* = 0.05. AM iter = 40

Comb. BOOT iter 94 done out of 120

cv : 1 out of 7 done. AM iter = 59

cv : 2 out of 7 done. AM iter = 58

cv : 3 out of 7 done. AM iter = 62

cv : 4 out of 7 done. AM iter = 59

cv : 5 out of 7 done. AM iter = 42

cv : 6 out of 7 done. AM iter = 31

cv : 7 out of 7 done. AM iter = 16

model id done, even death, mu = lambda, lambda* = 0.05. AM iter = 38

Comb. BOOT iter 95 done out of 120

cv : 1 out of 7 done. AM iter = 97

cv : 2 out of 7 done. AM iter = 102

cv : 3 out of 7 done. AM iter = 112

cv : 4 out of 7 done. AM iter = 137

cv : 5 out of 7 done. AM iter = 57

cv : 6 out of 7 done. AM iter = 37

cv : 7 out of 7 done. AM iter = 17

model id done, even death, mu = lambda, lambda* = 0.05. AM iter = 78

Comb. BOOT iter 96 done out of 120

cv : 1 out of 7 done. AM iter = 47

cv : 2 out of 7 done. AM iter = 47

cv : 3 out of 7 done. AM iter = 78

cv : 4 out of 7 done. AM iter = 66

cv : 5 out of 7 done. AM iter = 42

cv : 6 out of 7 done. AM iter = 34

cv : 7 out of 7 done. AM iter = 18

model id done, even death, mu = lambda, lambda* = 0.05. AM iter = 45

Comb. BOOT iter 97 done out of 120

cv : 1 out of 7 done. AM iter = 567

cv : 2 out of 7 done. AM iter = 212

cv : 3 out of 7 done. AM iter = 67

cv : 4 out of 7 done. AM iter = 43

cv : 5 out of 7 done. AM iter = 32

cv : 6 out of 7 done. AM iter = 24

cv : 7 out of 7 done. AM iter = 15

model id done, even death, mu = lambda, lambda* = 0.01. AM iter = 24

Comb. BOOT iter 98 done out of 120

cv : 1 out of 7 done. AM iter = 166

cv : 2 out of 7 done. AM iter = 189

cv : 3 out of 7 done. AM iter = 114

cv : 4 out of 7 done. AM iter = 84

cv : 5 out of 7 done. AM iter = 38

cv : 6 out of 7 done. AM iter = 28

cv : 7 out of 7 done. AM iter = 15

model id done, even death, mu = lambda, lambda* = 0.05. AM iter = 39

Comb. BOOT iter 99 done out of 120

cv : 1 out of 7 done. AM iter = 34

cv : 2 out of 7 done. AM iter = 35

cv : 3 out of 7 done. AM iter = 40

cv : 4 out of 7 done. AM iter = 90

cv : 5 out of 7 done. AM iter = 32

cv : 6 out of 7 done. AM iter = 24

cv : 7 out of 7 done. AM iter = 16

model id done, even death, mu = lambda, lambda* = 0.01. AM iter = 25

Comb. BOOT iter 100 done out of 120

cv : 1 out of 7 done. AM iter = 43

cv : 2 out of 7 done. AM iter = 60

cv : 3 out of 7 done. AM iter = 73

cv : 4 out of 7 done. AM iter = 125

cv : 5 out of 7 done. AM iter = 38

cv : 6 out of 7 done. AM iter = 28

cv : 7 out of 7 done. AM iter = 16

model id done, even death, mu = lambda, lambda* = 0.05. AM iter = 70

Comb. BOOT iter 101 done out of 120

cv : 1 out of 7 done. AM iter = 56

cv : 2 out of 7 done. AM iter = 55

cv : 3 out of 7 done. AM iter = 53

cv : 4 out of 7 done. AM iter = 74

cv : 5 out of 7 done. AM iter = 45

cv : 6 out of 7 done. AM iter = 33

cv : 7 out of 7 done. AM iter = 18

model id done, even death, mu = lambda, lambda* = 0.1. AM iter = 52

Comb. BOOT iter 102 done out of 120

cv : 1 out of 7 done. AM iter = 80

cv : 2 out of 7 done. AM iter = 85

cv : 3 out of 7 done. AM iter = 121

cv : 4 out of 7 done. AM iter = 128

cv : 5 out of 7 done. AM iter = 41

cv : 6 out of 7 done. AM iter = 26

cv : 7 out of 7 done. AM iter = 14

model id done, even death, mu = lambda, lambda* = 0.01. AM iter = 25

Comb. BOOT iter 103 done out of 120

cv : 1 out of 7 done. AM iter = 61

cv : 2 out of 7 done. AM iter = 60

cv : 3 out of 7 done. AM iter = 104

cv : 4 out of 7 done. AM iter = 169

cv : 5 out of 7 done. AM iter = 53

cv : 6 out of 7 done. AM iter = 37

cv : 7 out of 7 done. AM iter = 19

model id done, even death, mu = lambda, lambda* = 0.05. AM iter = 51

Comb. BOOT iter 104 done out of 120

cv : 1 out of 7 done. AM iter = 58

cv : 2 out of 7 done. AM iter = 60

cv : 3 out of 7 done. AM iter = 68

cv : 4 out of 7 done. AM iter = 59

cv : 5 out of 7 done. AM iter = 57

cv : 6 out of 7 done. AM iter = 38

cv : 7 out of 7 done. AM iter = 19

model id done, even death, mu = lambda, lambda* = 0.05. AM iter = 48

Comb. BOOT iter 105 done out of 120

cv : 1 out of 7 done. AM iter = 89

cv : 2 out of 7 done. AM iter = 110

cv : 3 out of 7 done. AM iter = 95

cv : 4 out of 7 done. AM iter = 117

cv : 5 out of 7 done. AM iter = 46

cv : 6 out of 7 done. AM iter = 33

cv : 7 out of 7 done. AM iter = 17

model id done, even death, mu = lambda, lambda* = 0.01. AM iter = 32

Comb. BOOT iter 106 done out of 120

cv : 1 out of 7 done. AM iter = 103

cv : 2 out of 7 done. AM iter = 98

cv : 3 out of 7 done. AM iter = 88

cv : 4 out of 7 done. AM iter = 90

cv : 5 out of 7 done. AM iter = 40

cv : 6 out of 7 done. AM iter = 27

cv : 7 out of 7 done. AM iter = 14

model id done, even death, mu = lambda, lambda* = 0.05. AM iter = 56

Comb. BOOT iter 107 done out of 120

cv : 1 out of 7 done. AM iter = 75

cv : 2 out of 7 done. AM iter = 92

cv : 3 out of 7 done. AM iter = 51

cv : 4 out of 7 done. AM iter = 39

cv : 5 out of 7 done. AM iter = 33

cv : 6 out of 7 done. AM iter = 25

cv : 7 out of 7 done. AM iter = 16

model id done, even death, mu = lambda, lambda* = 0.01. AM iter = 22

Comb. BOOT iter 108 done out of 120

cv : 1 out of 7 done. AM iter = 129

cv : 2 out of 7 done. AM iter = 93

cv : 3 out of 7 done. AM iter = 48

cv : 4 out of 7 done. AM iter = 45

cv : 5 out of 7 done. AM iter = 35

cv : 6 out of 7 done. AM iter = 25

cv : 7 out of 7 done. AM iter = 17

model id done, even death, mu = lambda, lambda* = 0.05. AM iter = 36

Comb. BOOT iter 109 done out of 120

cv : 1 out of 7 done. AM iter = 93

cv : 2 out of 7 done. AM iter = 89

cv : 3 out of 7 done. AM iter = 62

cv : 4 out of 7 done. AM iter = 50

cv : 5 out of 7 done. AM iter = 39

cv : 6 out of 7 done. AM iter = 30

cv : 7 out of 7 done. AM iter = 18

model id done, even death, mu = lambda, lambda* = 0.05. AM iter = 51

Comb. BOOT iter 110 done out of 120

cv : 1 out of 7 done. AM iter = 28

cv : 2 out of 7 done. AM iter = 45

cv : 3 out of 7 done. AM iter = 35

cv : 4 out of 7 done. AM iter = 41

cv : 5 out of 7 done. AM iter = 27

cv : 6 out of 7 done. AM iter = 19

cv : 7 out of 7 done. AM iter = 14

model id done, even death, mu = lambda, lambda* = 0.01. AM iter = 23

Comb. BOOT iter 111 done out of 120

cv : 1 out of 7 done. AM iter = 125

cv : 2 out of 7 done. AM iter = 160

cv : 3 out of 7 done. AM iter = 88

cv : 4 out of 7 done. AM iter = 119

cv : 5 out of 7 done. AM iter = 41

cv : 6 out of 7 done. AM iter = 31

cv : 7 out of 7 done. AM iter = 17

model id done, even death, mu = lambda, lambda* = 0.01. AM iter = 30

Comb. BOOT iter 112 done out of 120

cv : 1 out of 7 done. AM iter = 42

cv : 2 out of 7 done. AM iter = 45

cv : 3 out of 7 done. AM iter = 44

cv : 4 out of 7 done. AM iter = 32

cv : 5 out of 7 done. AM iter = 34

cv : 6 out of 7 done. AM iter = 22

cv : 7 out of 7 done. AM iter = 16

model id done, even death, mu = lambda, lambda* = 0.05. AM iter = 28

Comb. BOOT iter 113 done out of 120

cv : 1 out of 7 done. AM iter = 176

cv : 2 out of 7 done. AM iter = 179

cv : 3 out of 7 done. AM iter = 361

cv : 4 out of 7 done. AM iter = 94

cv : 5 out of 7 done. AM iter = 37

cv : 6 out of 7 done. AM iter = 28

cv : 7 out of 7 done. AM iter = 16

model id done, even death, mu = lambda, lambda* = 0.01. AM iter = 26

Comb. BOOT iter 114 done out of 120

cv : 1 out of 7 done. AM iter = 33

cv : 2 out of 7 done. AM iter = 44

cv : 3 out of 7 done. AM iter = 117

cv : 4 out of 7 done. AM iter = 78

cv : 5 out of 7 done. AM iter = 41

cv : 6 out of 7 done. AM iter = 25

cv : 7 out of 7 done. AM iter = 17

model id done, even death, mu = lambda, lambda* = 0.05. AM iter = 71

Comb. BOOT iter 115 done out of 120

cv : 1 out of 7 done. AM iter = 228

cv : 2 out of 7 done. AM iter = 67

cv : 3 out of 7 done. AM iter = 100

cv : 4 out of 7 done. AM iter = 71

cv : 5 out of 7 done. AM iter = 46

cv : 6 out of 7 done. AM iter = 31

cv : 7 out of 7 done. AM iter = 16

model id done, even death, mu = lambda, lambda* = 0.01. AM iter = 28

Comb. BOOT iter 116 done out of 120

cv : 1 out of 7 done. AM iter = 62

cv : 2 out of 7 done. AM iter = 105

cv : 3 out of 7 done. AM iter = 64

cv : 4 out of 7 done. AM iter = 39

cv : 5 out of 7 done. AM iter = 28

cv : 6 out of 7 done. AM iter = 22

cv : 7 out of 7 done. AM iter = 14

model id done, even death, mu = lambda, lambda* = 0.05. AM iter = 27

Comb. BOOT iter 117 done out of 120

cv : 1 out of 7 done. AM iter = 119

cv : 2 out of 7 done. AM iter = 115

cv : 3 out of 7 done. AM iter = 110

cv : 4 out of 7 done. AM iter = 95

cv : 5 out of 7 done. AM iter = 49

cv : 6 out of 7 done. AM iter = 33

cv : 7 out of 7 done. AM iter = 17

model id done, even death, mu = lambda, lambda* = 0.05. AM iter = 97

Comb. BOOT iter 118 done out of 120

cv : 1 out of 7 done. AM iter = 84

cv : 2 out of 7 done. AM iter = 74

cv : 3 out of 7 done. AM iter = 115

cv : 4 out of 7 done. AM iter = 62

cv : 5 out of 7 done. AM iter = 43

cv : 6 out of 7 done. AM iter = 33

cv : 7 out of 7 done. AM iter = 18

model id done, even death, mu = lambda, lambda* = 0.5. AM iter = 58

Comb. BOOT iter 119 done out of 120

cv : 1 out of 7 done. AM iter = 137

cv : 2 out of 7 done. AM iter = 142

cv : 3 out of 7 done. AM iter = 144

cv : 4 out of 7 done. AM iter = 154

cv : 5 out of 7 done. AM iter = 64

cv : 6 out of 7 done. AM iter = 41

cv : 7 out of 7 done. AM iter = 20

model id done, even death, mu = lambda, lambda* = 0.05. AM iter = 68

Comb. BOOT iter 120 done out of 120

DMSO: max # AM iter = 1000, hit 4x out of 120 total.

Tram.: max # AM iter = 1000, hit 1x out of 120 total.

BEZ: max # AM iter = 261, hit 1x out of 120 total.

Comb.: max # AM iter = 406, hit 1x out of 120 total.

>>
