# Supplementary figures and images for: Modeling differentiation-state transitions linked to therapeutic escape in triple-negative breast cancer
Source: PLoS Comput Biol. 2019 Mar 11;15(3):e1006840. doi: 10.1371/journal.pcbi.1006840 (PMC6428348; doi:10.1371/journal.pcbi.1006840)

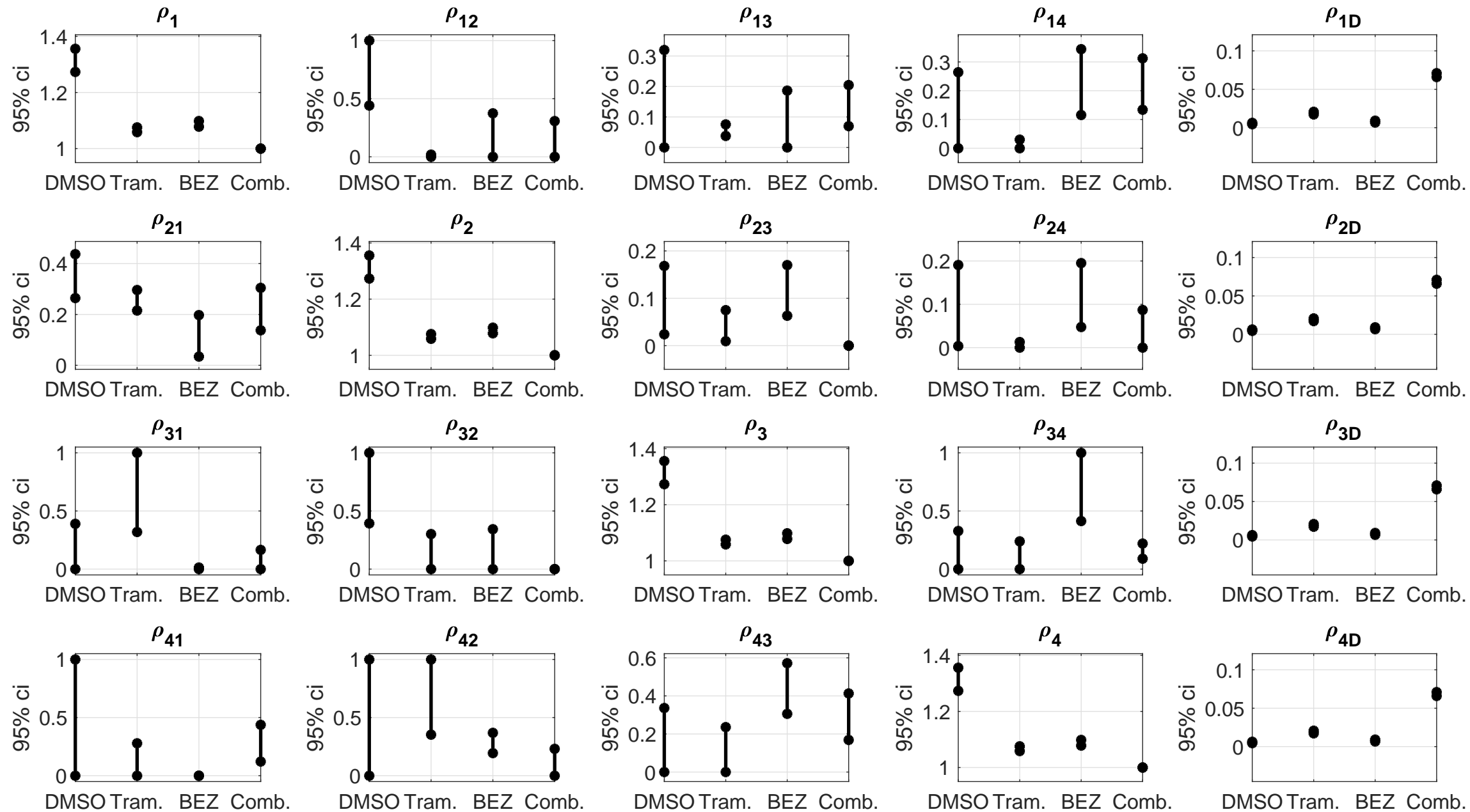

Supplement: S1 Code/Training Data/Test Data — The experimental data and code used to generate the computational results of this paper are provided. MATLAB (The MathWorks, Inc.) and CVX software [52] are required. The raw training data is in the file Timeseries_Raw_15wells.xlsx, and the raw test data is in the file Timeseries_Raw_4wells.xlsx. (ZIP) [file pcbi.1006840.s001.zip › S1_Code_TrainData_TestData/Results_Summer_2017/bootstrap/ci_K14K19VIM_oct302018.pdf]

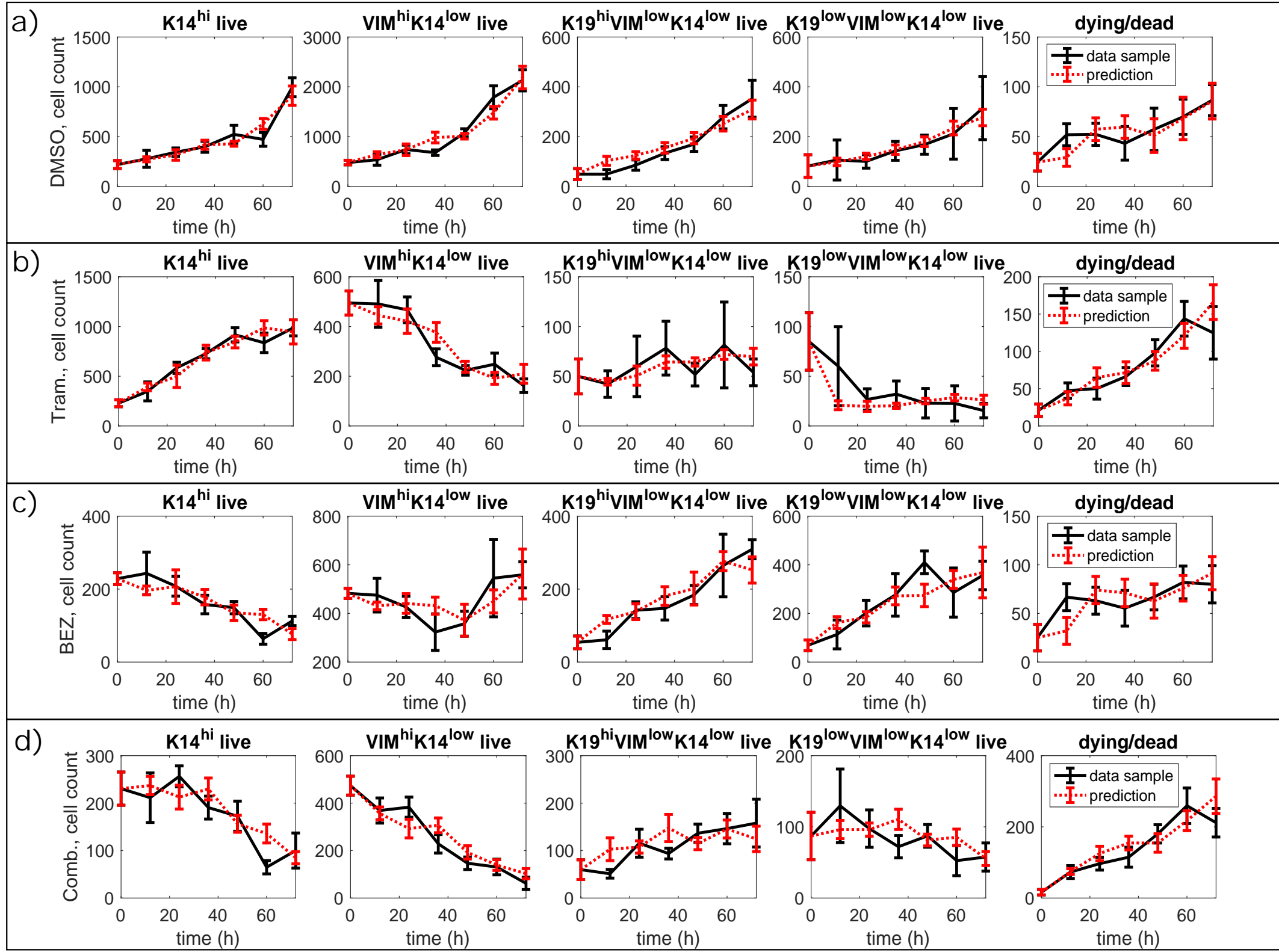

Supplement: S1 Code/Training Data/Test Data — The experimental data and code used to generate the computational results of this paper are provided. MATLAB (The MathWorks, Inc.) and CVX software [52] are required. The raw training data is in the file Timeseries_Raw_15wells.xlsx, and the raw test data is in the file Timeseries_Raw_4wells.xlsx. (ZIP) [file pcbi.1006840.s001.zip › S1_Code_TrainData_TestData/Revisions_Fall2018/Ensemble_Modeling/BEFOREENSEMBLE_ModelPred_and_TrainingData/alldrugs_pred_and_train.pdf]

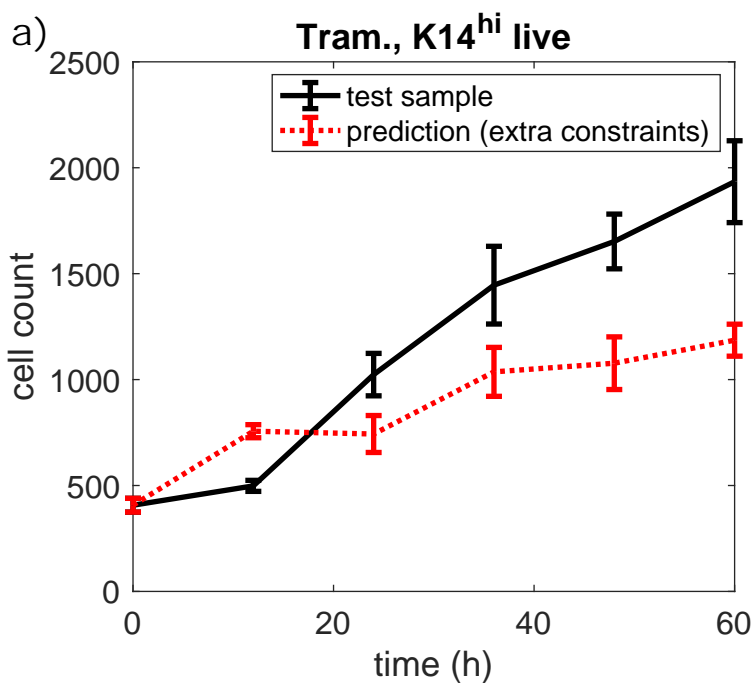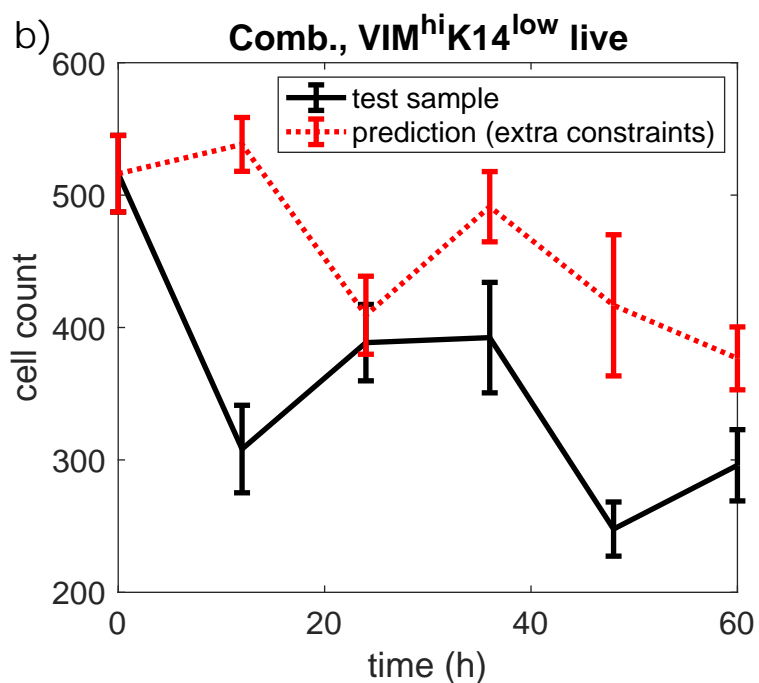

Supplement: S1 Code/Training Data/Test Data — The experimental data and code used to generate the computational results of this paper are provided. MATLAB (The MathWorks, Inc.) and CVX software [52] are required. The raw training data is in the file Timeseries_Raw_15wells.xlsx, and the raw test data is in the file Timeseries_Raw_4wells.xlsx. (ZIP) [file pcbi.1006840.s001.zip › S1_Code_TrainData_TestData/Trying_Out_Hyp_Results_Fall2017/figs5and6.pdf]

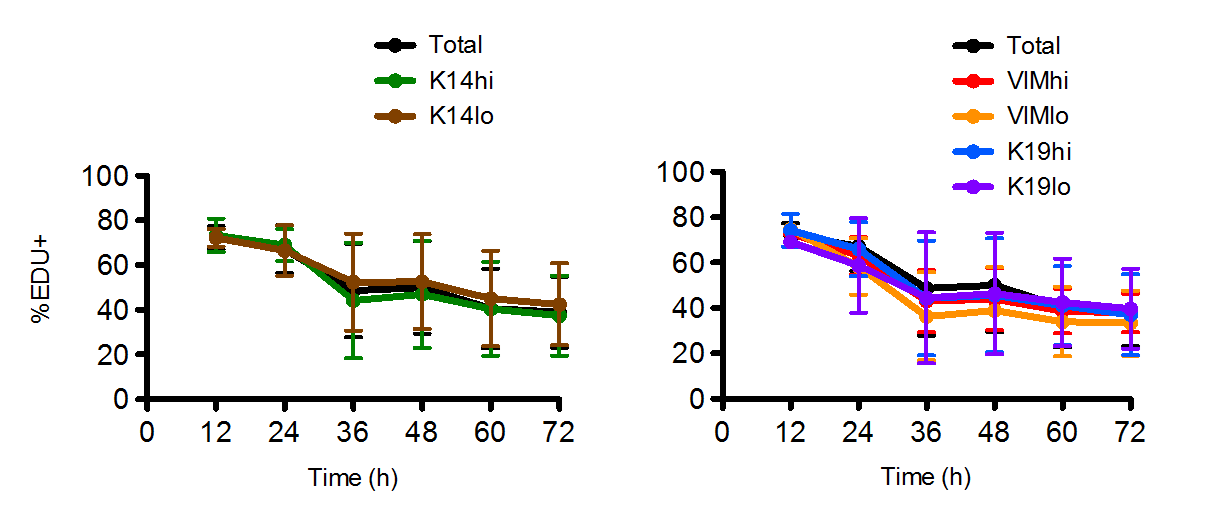

Supplement: S1 Figure — The percentages of EdU-positive DMSO-treated HCC1143 cells for each differentiation-state marker expression level are shown. The data were collected via the cell cycle analysis methods of [18]. (TIF) [file pcbi.1006840.s003.tif]

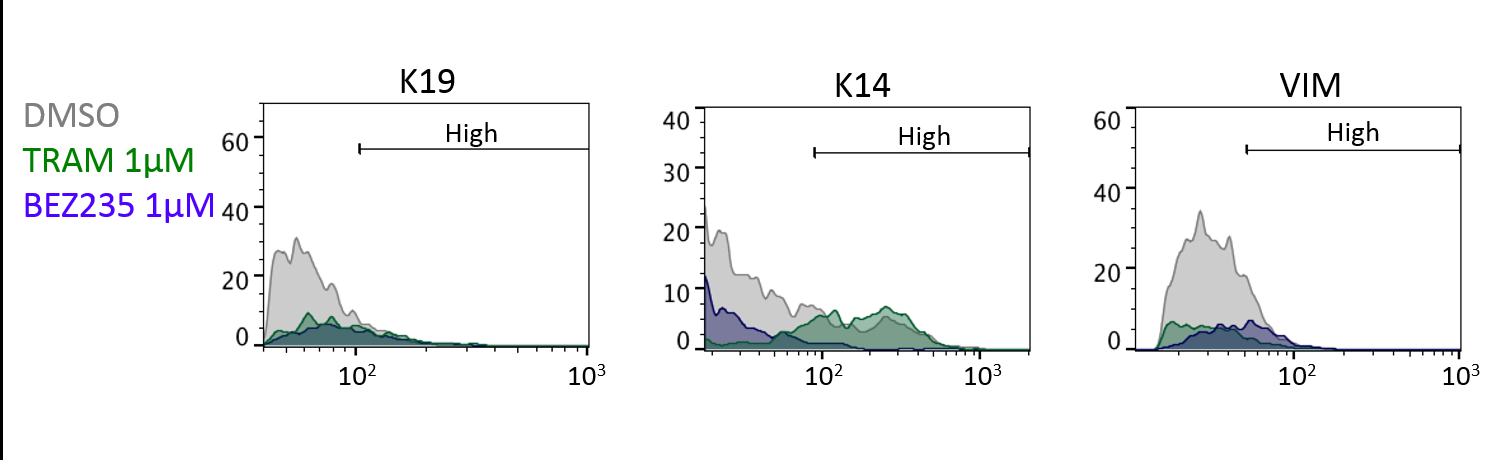

Supplement: S2 Figure — HCC1143 cells were treated with either DMSO (gray), 1μM Trametinib (green), or 1μM BEZ235 (blue) for 72h, then fixed and stained with antibodies against Cytokeratin 19 (K19), Cytokeratin 14 (K14), and Vimentin (VIM). Cells were imaged and single-cell mean-fluorescent intensities were calculated using image cytometry software [18] and displayed in a histogram. (TIF) [file pcbi.1006840.s004.tif]
