# Supplementary material for: Modeling differentiation-state transitions linked to therapeutic escape in triple-negative breast cancer
Source: PLoS Comput Biol. 2019 Mar 11;15(3):e1006840. doi: 10.1371/journal.pcbi.1006840 (PMC6428348; doi:10.1371/journal.pcbi.1006840)
Supplement: S1 Appendix — This document provides additional details on the mathematical and numerical methods. (PDF) [file pcbi.1006840.s007.pdf]

Mathematical and Numerical Methods

Supporting Information for Chapman *et al.*

1 Contents

This document provides details on the mathematical and numerical methods: model (Sec. 2), data (Sec. 3), merging the phenotype and death data (Sec. 4), system identification with preliminary analysis (Sec. 5), model testing (Sec. 6), and uncertainty analysis (Sec. 7).

2 Mathematical Model

The mathematical model represents how $x \in \mathbb{R}^5$, the cell type vector, evolves over time in response to drug treatment. x_1 is the number of $\mathrm{K14^{hi}}$ live cells, x_2 is the number of $\mathrm{VIM^{hi}K14^{low}}$ live cells, x_3 is the number of $\mathrm{K19^{hi}VIM^{low}K14^{low}}$ live cells, x_4 is the number of $\mathrm{K19^{low}VIM^{low}K14^{low}}$ live cells, and x_5 is the number of dead or dying cells. The model assumes that a live cell can do one of three mutually exclusive actions—divide, transition, or die—during any 12h interval. The dynamics matrix $A_\delta \in \mathbb{R}^{5\times5}$ dictates the evolution of the cell type vector x and encodes how often division, transition, and death occur on average in a cancer cell population following treatment with drug δ. Recall from the main manuscript that ρ_i is the division parameter of phenotypic state i, ρ_{ij} is the transition gain from phenotypic state i to phenotypic state j, and $\rho_{i\mathrm{D}}$ is the death gain of phenotypic state i. The division parameters, transition gains, and death gains are called *dynamics parameters.* Each dynamics parameter depends on drug δ. For example, $\rho_1 = \rho_1(\delta)$, $\rho_{34} = \rho_{34}(\delta)$, and $\rho_{2\mathrm{D}} = \rho_{2\mathrm{D}}(\delta)$. The dynamics matrix A_δ takes the form,

$$A_\delta = \begin{bmatrix} \alpha_1 & \rho_{21} & \rho_{31} & \rho_{41} & 0 \\ \rho_{12} & \alpha_2 & \rho_{32} & \rho_{42} & 0 \\ \rho_{13} & \rho_{23} & \alpha_3 & \rho_{43} & 0 \\ \rho_{14} & \rho_{24} & \rho_{34} & \alpha_4 & 0 \\ \rho_{1\mathrm{D}} & \rho_{2\mathrm{D}} & \rho_{3\mathrm{D}} & \rho_{4\mathrm{D}} & 1 \end{bmatrix}$$
$$\alpha_i = \rho_i - \rho_{i\mathrm{D}} - \textstyle\sum_{s=1,s\neq i}^{4} \rho_{is} \qquad i = 1,\dots,4. \tag{1}$$

For example, $\alpha_2 = \rho_2 - \rho_{2\mathrm{D}} - \rho_{21} - \rho_{23} - \rho_{24}$. A_δ is subject to a set of constraints called $\mathcal{A}$, which are provided in Table 1. Eq. (1) is derived in [1].

Definition 2.1. The seven constraints listed in Table 1 are denoted $\mathcal{A} \subset \mathbb{R}^{5\times5}$.

Remark. The dynamics matrix $A_\delta \in \mathcal{A}$ has 14 parameters.

Definition 2.2. The first six constraints listed in Table 1 are denoted $\mathcal{A}_{\mathrm{pre}} \subset \mathbb{R}^{5\times5}$.

3 Data for Estimating A_δ

A sample measured at time k of the cell population in well w consists of the number of cells in each phenotypic state, the number of dying cells, and the number of live or dying cells in total. The set of numbers of cells in each phenotypic state is the *phenotype time series*, and the set of numbers of dying cells and numbers of live or dying cells in total is the *death time series*. These data do not readily fit the form of the cell type vector x. The cell type vector contains the number of *live* cells in each phenotypic state. The data do not provide the number of live cells in each phenotypic state or the number of dying cells in each phenotypic state. The phenotypic state of a given cell and whether that cell is alive or dying could not be observed simultaneously.

Table 2 provides the number of samples used to estimate the 20 drug-specific dynamics parameters encoded in A_δ. If instrument errors had not occurred, then 105 samples would have been available to estimate each A_δ.

4 Merging the Phenotype and Death Data

This section presents how we partitioned the number of cells in a given phenotypic state into the number of live cells and the number of dying cells in that state for modelling purposes. We merged the phenotype time series and the death time series in different ways based on how death could be allocated between two phenotypes, K14^{hi} and K14^{low}. (K14^{low} is the union of $\text{VIM}^{\text{hi}}\text{K14}^{\text{low}}$, $\text{K19}^{\text{hi}}\text{VIM}^{\text{low}}\text{K14}^{\text{low}}$, and $\text{K19}^{\text{low}}\text{VIM}^{\text{low}}\text{K14}^{\text{low}}$.) A small number of *death options* were examined to ensure a parsimonious model.

Table 1: Constraints on A_δ (1)

	Constraint	Rationale
1	Each entry of A_δ is nonnegative.	All entries of the cell type vector x are non-negative for all time.
2	$\rho_i \geq 1$; $i \in \{1, \ldots, 4\}$	$\rho_i \cdot x_i(k) - x_i(k)$ is the increase in the number of live cells in phenotypic state i due to replication on $[k, k+1)$.
3	$\rho_{ij} \leq 1$; $(i,j) \in \{1, \ldots, 4\}^2$, $i \neq j$	Only a portion of live cells in phenotypic state i at time k can transition to phenotypic state j by time $k+1$.
4	$\rho_{iD} \leq 1$; $i \in \{1, \ldots, 4\}$	Only a portion of live cells in phenotypic state i at time k can die, or begin to die, by time $k+1$.
5	Last column of A_δ is $[0, ..., 0, 1]^T \in \mathbb{R}^5$	Dead or dying cells accumulate over time and cannot come back to life.
6	$\rho_i = \rho_1$; $i \in \{2, \ldots, 4\}$	Observations of EdU-positivity suggest similar rates of cell division for the phenotypic states [2].
7	$\rho_{i\text{D}} = \rho_{1\text{D}}$; $i \in \{2, \ldots, 4\}$	Motivated by preliminary analyses (Sec. 5).

Table 2: Numbers of available and unavailable samples

Drug	DMSO	Trametinib	BEZ235	Trametinib+BEZ235
Available samples	99	90	93	93
Unavailable samples	6	15	12	12

A (time k, well w)-sample is said to be *unavailable* if the images of the phenotypic states, the live or dying cells in total, or the dying cells taken at time k of well w are illegible.

Definition 4.1. *Death option 1.* Minimal death is allocated to $\mathrm{K14^{hi}}$; maximal death is allocated to $\mathrm{K14^{low}}$.

Definition 4.2. *Death option 2.* Death is allocated equally between $\mathrm{K14^{hi}}$ and $\mathrm{K14^{low}}$.

Definition 4.3. *Death option 3.* Minimal death is allocated to $\mathrm{K14^{low}}$; maximal death is allocated to $\mathrm{K14^{hi}}$.

The cell type vector $x = x(k, w; o)$ for the sample observed at time k in well w under death option o was computed from the data,

$$x = \begin{bmatrix} y_1(1 - \phi \cdot \gamma^{\mathrm{hi}}) \\ y_2(1 - \phi \cdot \gamma^{\mathrm{low}}) \\ y_3(1 - \phi \cdot \gamma^{\mathrm{low}}) \\ y_4(1 - \phi \cdot \gamma^{\mathrm{low}}) \\ \phi \cdot \sum_{i=1}^{4} y_i \end{bmatrix}, \tag{2}$$

where $y_i = y_i(k, w)$ is the number of cells in phenotypic state i observed from the phenotype time series, $\phi = \phi(k, w)$ is the dying fraction observed from the death time series, and $\gamma^{\mathrm{hi}} = \gamma^{\mathrm{hi}}(k, w; o)$ and $\gamma^{\mathrm{low}} = \gamma^{\mathrm{low}}(k, w; o)$ are *death allocation hyper-parameters* (Table 3, Sec. 4.1).

Definition 4.4. The phenotype time series data and the death time series data merged together via (2) is called *composite data.*

4.1 Death Allocation Hyper-parameters

The death allocation hyper-parameters, $\gamma^{\mathrm{hi}} = \gamma^{\mathrm{hi}}(k, w; o)$ and $\gamma^{\mathrm{low}} = \gamma^{\mathrm{low}}(k, w; o)$, allocate the death observed at time k in well w among the phenotypic states according to death option o. The death allocation hyper-parameters are defined implicitly,

$$\begin{aligned} d^{\mathrm{hi}} &= \gamma^{\mathrm{hi}} \cdot \phi \cdot y_1 \\ d^{\mathrm{low}} &= \gamma^{\mathrm{low}} \cdot \phi \cdot \textstyle\sum_{i=2}^{4} y_i, \end{aligned} \tag{3}$$

in terms of the number of $\mathrm{K14^{hi}}$ dead or dying cells estimated *in silico* $\big(d^{\mathrm{hi}} = d^{\mathrm{hi}}(k, w)\big)$, the number of $\mathrm{K14^{low}}$ dead or dying cells estimated *in silico* $\big(d^{\mathrm{low}} = d^{\mathrm{low}}(k, w)\big)$, and the empirical data $\big(y_i = y_i(k, w)$ and $\phi = \phi(k, w)\big)$; refer to Table 3.

Next, we state and prove three theoretical results needed to compute the values of γ^{hi} and γ^{low} for a given sample.

Remark. The dying fraction, ϕ, and the number of cells in phenotypic state i, y_i, are positive for all available samples.

Lemma 4.1. The death allocation hyper-parameters, γ^{hi} and γ^{low}, satisfy the equality,

$$\textstyle\sum_{i=1}^{4} y_i = \gamma^{\text{hi}} \cdot y_1 + \gamma^{\text{low}} \cdot \sum_{i=2}^{4} y_i. \tag{4}$$

Proof. The number of dead or dying cells can be expressed in two equivalent ways,

$$\begin{aligned} \#\text{ dead or dying cells} &= \phi \cdot \textstyle\sum_{i=1}^{4} y_i \\ \#\text{ dead or dying cells} &= d^{\text{hi}} + d^{\text{low}}. \end{aligned} \tag{5}$$

The first line of (5) comes from the cell type vector (2). The second line of (5) holds because any dying cell is either in K14^{hi} or K14^{low} but not both.

Using (5) and (3), we have

$$\begin{aligned} \phi \cdot \textstyle\sum_{i=1}^{4} y_i &= d^{\text{hi}} + d^{\text{low}} \\ \phi \cdot \textstyle\sum_{i=1}^{4} y_i &= \gamma^{\text{hi}} \cdot \phi \cdot y_1 + \gamma^{\text{low}} \cdot \phi \cdot \textstyle\sum_{i=2}^{4} y_i. \end{aligned} \tag{6}$$

Divide the second line of (6) by $\phi > 0$ to obtain (4). □

Lemma 4.2. γ^{hi} and γ^{low} satisfy the inequalites,

$$\begin{aligned} 0 &\leq \gamma^{\text{hi}} \leq \frac{1}{\phi} \\ 0 &\leq \gamma^{\text{low}} \leq \frac{1}{\phi}. \end{aligned} \tag{7}$$

Table 3: Notation for Sec. 4

Symbol	Definition
y_i; $i = 1, \ldots, 4$	$y_i = y_i(k, w)$ is the number of cells in phenotypic state i observed at time k in well w from the phenotype time series data.
ϕ	$\phi = \phi(k, w)$ is the number of dying cells divided by the number of live or dying cells in total observed at time k in well w from the death time series data.
γ^{hi}	$\gamma^{\text{hi}} = \gamma^{\text{hi}}(k, w; o)$ is the K14^{hi} death allocation hyper-parameter for the sample observed at time k in well w assuming death option o.
γ^{low}	$\gamma^{\text{low}} = \gamma^{\text{low}}(k, w; o)$ is the K14^{low} death allocation hyper-parameter for the sample observed at time k in well w assuming death option o.
d^{hi}	$d^{\text{hi}} = d^{\text{hi}}(k, w; o)$ is the number of dead or dying cells in K14^{hi} estimated *in silico* from the (time k, well w)-sample assuming death option o.
d^{low}	$d^{\text{low}} = d^{\text{low}}(k, w; o)$ is the number of dead or dying cells in K14^{low} estimated *in silico* from the (time k, well w)-sample assuming death option o.

Proof. The number of dead or dying cells in phenotypic state $\mathrm{K14^{low}}$ is nonnegative and at most equal to the total number of cells in $\mathrm{K14^{low}}$,

$$0 \leq d^{\mathrm{low}} \leq \textstyle\sum_{i=2}^{4} y_i. \tag{8}$$

Combining (3) and (8) yields,

$$0 \leq \gamma^{\mathrm{low}} \cdot \phi \cdot \textstyle\sum_{i=2}^{4} y_i \leq \sum_{i=2}^{4} y_i. \tag{9}$$

Divide (9) by $\phi \cdot \sum_{i=2}^{4} y_i > 0$ to obtain,

$$0 \leq \gamma^{\mathrm{low}} \leq \frac{1}{\phi}. \tag{10}$$

Deriving $0 \leq \gamma^{\mathrm{hi}} \leq \frac{1}{\phi}$ is analogous. □

The values of the death allocation hyper-parameters were determined for each death option o and data sample by minimizing the cost function, $J_o = J_o(\gamma^{\mathrm{hi}}, \gamma^{\mathrm{low}})$, subject to the constraints, (4) and (7). The cost functions are,

$$\begin{aligned} J_1 &= \gamma^{\mathrm{hi}} - \gamma^{\mathrm{low}} \\ J_2 &= |\gamma^{\mathrm{hi}} - \gamma^{\mathrm{low}}| \\ J_3 &= \gamma^{\mathrm{low}} - \gamma^{\mathrm{hi}}. \end{aligned} \tag{11}$$

We chose (11) because γ^{hi} and γ^{low} are proportional to the amount of dead or dying cells in the phenotypic states, $\mathrm{K14^{hi}}$ and $\mathrm{K14^{low}}$, respectively (3). If $o = 1$, the optimization program should minimize the amount of death allocated to $\mathrm{K14^{hi}}$ and maximize the amount of death allocated to $\mathrm{K14^{low}}$. So, γ^{hi} should be made as small as possible, while γ^{low} should be made as large as possible, yielding the form of J_1 in (11). The logic underlying the form of J_3 is analogous. If $o = 2$, the optimization program should evenly distribute the amount of death across the phenotypic states. Thus, the deviation between γ^{hi} and γ^{low} should be made as small as possible, which justifies the form of J_2 in (11).

The values of the death allocation hyper-parameters can be determined analytically, as stated in the following lemma.

Lemma 4.3. The optimal arguments $(\gamma^{*\mathrm{hi}}, \gamma^{*\mathrm{low}})$ of the program,

$$\begin{aligned} &\underset{\gamma^{\mathrm{hi}},\gamma^{\mathrm{low}}}{\text{minimize}} && J_o(\gamma^{\mathrm{hi}}, \gamma^{\mathrm{low}}) && (11) \\ &\text{subject to} && \textstyle\sum_{i=1}^{4} y_i = \gamma^{\mathrm{hi}} \cdot y_1 + \gamma^{\mathrm{low}} \cdot \sum_{i=2}^{4} y_i && (4) \\ &&& 0 \leq \gamma^{\mathrm{hi}} \leq \frac{1}{\phi},\ 0 \leq \gamma^{\mathrm{low}} \leq \frac{1}{\phi} && (7) \end{aligned} \tag{12}$$

are given below.

If $o = 1$,

$$\begin{aligned} \gamma^{*\mathrm{low}} &= \min\Big(\frac{y_1}{\sum_{i=2}^{4} y_i} + 1, \frac{1}{\phi}\Big) \\ \gamma^{*\mathrm{hi}} &= \frac{\sum_{i=2}^{4} y_i}{y_1}(1 - \gamma^{*\mathrm{low}}) + 1. \end{aligned} \tag{13}$$

If $o = 2$,

$$\gamma^{*\text{low}} = \gamma^{*\text{hi}} = 1. \tag{14}$$

If $o = 3$,

$$\begin{aligned} \gamma^{*\text{hi}} &= \min\Big(\frac{\sum_{i=2}^{4} y_i}{y_1} + 1, \frac{1}{\phi}\Big) \\ \gamma^{*\text{low}} &= \frac{y_1}{\sum_{i=2}^{4} y_i}(1 - \gamma^{*\text{hi}}) + 1. \end{aligned} \tag{15}$$

The notation above is defined in Table 3, and "$\min(a, b)$" is the minimum of a and b.

Proof. For $o = 1$, the optimization program (12) aims to minimize γ^{hi} and maximize γ^{low}. The optimal arguments $(\gamma^{*\text{hi}}, \gamma^{*\text{low}})$ reside on the line (4) and within the box (7) in the γ^{hi}-γ^{low} coordinate plane. Line (4) passes through the following two points: *a*) $\big(0, \frac{y_1}{\sum_{i=2}^{4} y_i} + 1\big)$ and *b*) $\big(\frac{\sum_{i=2}^{4} y_i}{y_1} \cdot (1 - \frac{1}{\phi}) + 1, \frac{1}{\phi}\big)$. Point a achieves the lower bound of γ^{hi}. Thus, point a is optimal if its γ^{low} value satisfies (7), namely if $\frac{y_1}{\sum_{i=2}^{4} y_i} + 1 \leq \frac{1}{\phi}$. Otherwise, the optimal value of γ^{hi} is slightly greater than zero, and the optimal value of γ^{low} is its upper bound, $\frac{1}{\phi}$; in other words, point b is optimal. This result is stated concisely in (13). The logic for $o = 3$ is analogous to that for $o = 1$. For $o = 2$, $\gamma^{*\text{hi}} = \gamma^{*\text{low}} = 1$ satisfies the constraints, (4) and (7), and achieves the minimum cost function value, $J_2 = 0$. □

Remark. Eq. (12) is a linear program for $o = 1$ and $o = 3$ and has a natural geometric interpretation (Fig. 1). See [3] for general reference on optimization.

5 System Identification

In the previous section, we showed how to partition the number of cells in a given phenotypic state into the number of live cells and the number of dying cells in that state. We explained how this partition can be done in different ways. Next, we will describe how to identify a dynamics matrix A_δ that fits the data sufficiently well and in the process how to select an appropriate partition.

We used a numerical system identification approach because A_δ is constrained and because some samples are missing (Table 1, Table 2). For each drug δ, the system identification problem is to solve the optimization program,

$$\begin{aligned} &\underset{A,X}{\text{minimize}} && \mathcal{J}(A, X; \lambda, \mu, o) \\ &\text{subject to} && A \in \mathcal{A},\ X \in \mathbb{R}_+^{5\times m}, \end{aligned} \tag{16}$$

for dynamics matrix $A_\delta \in \mathcal{A}$ and optimized data $X_\delta \in \mathbb{R}_+^{5\times m}$, a $5\times m$ matrix with nonnegative entries. $\mathcal{J}$ is a cost function parametrized by weights (λ, μ) and death option o; m is the number of samples used in the optimization. Once the values of (λ, μ, o) are chosen, m is

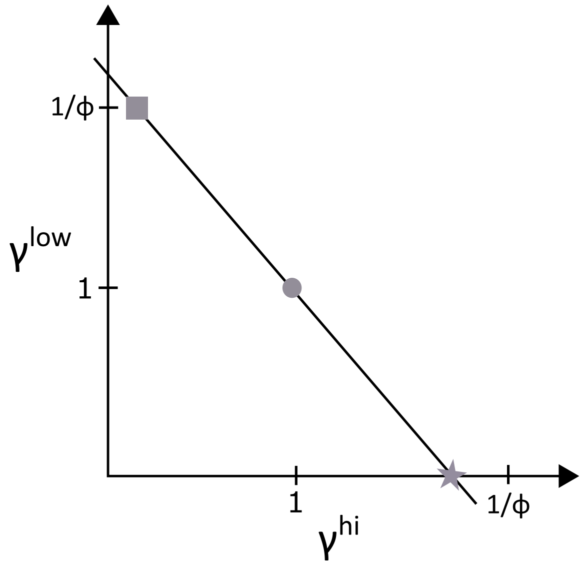

Figure 1: **Geometric interpretation of Lemma 4.3.** Let $(y'_1, y'_2, y'_3, y'_4, \phi')$ be a data sample. For illustration purposes, suppose $\frac{y'_1}{\sum_{i=2}^4 y'_i} + 1 > \frac{1}{\phi'}$ and $\frac{\sum_{i=2}^4 y'_i}{y'_1} + 1 < \frac{1}{\phi'}$. The optimal arguments $(\gamma^{*\text{hi}}, \gamma^{*\text{low}})$ for this sample are marked in the γ^{hi}-γ^{low} coordinate plane with a distinct shape for each death option o: square ($o = 1$), circle ($o = 2$), and star ($o = 3$).

105, the product of the number of time points (7) and the number of wells per time point (15).

The composite data and the optimized data are distinct. The composite data is computed directly from empirical measurements assuming death option o (2), has holes due to unavailable samples (Table 2), and is an input to the optimization program (16). The optimized data is an output of the program (16), has less measurement noise than the composite data, and includes estimates for the unavailable samples.

The cost function $\mathcal{J}$ in (16) was designed to accomplish three aims: a) to penalize process error and measurement error, b) to allow the covariance matrix of process error and the covariance matrix of measurement error to have unequal magnitudes, and c) to encourage the identified dynamics parameters to be close to their true (unknown) values. The above aims, if feasible, would facilitate learning a dynamics matrix, A_δ, that fits the composite data sufficiently well, where $\mathcal{J}$ measures how good the fit is.

The cost function in (16) takes the form,

$$\mathcal{J}(A, X; \lambda, \mu, o) = |A| + \lambda \cdot |\text{ProcessError}(A, X)| + \mu \cdot |\text{MeasurementError}(X; o)|, \tag{17}$$

such that $\lambda \in (0, 1]$ and $\mu \in (0, 1]$. ProcessError(A, X) is a process error metric and depends on the variables A and X. MeasurementError$(X; o)$ is a measurement error metric, depends on the variable X, and is parametrized by death option o. $|P|$ denotes the average size of an entry of the matrix P; see definition below.

Definition 5.1. If $P \in \mathbb{R}^{p \times q}$, then $|P| = \sqrt{\frac{1}{pq} \sum_{i,j} |P_{ij}|^2}$, where P_{ij} is the (i, j)-entry of P.

Specifying $\lambda \in (0, 1]$ and $\mu \in (0, 1]$ in (17) ensures that the size of A is penalized at least as much as the size of the process error and the size of the measurement error, respectively. The particular use of λ and μ in (17) induces element-wise shrinkage of A to zero, thereby reducing estimation error of the dynamics parameters [4][5].

Each column of ProcessError(A, X) in (17) takes the form,

$$x_{k+1,w} - A \cdot x_{k,w} \in \mathbb{R}^5, \tag{18}$$

where $x_{k,w} \in \mathbb{R}^5_+$ is the column of variable X associated with time k and well w.

Remark. The variables A and X are coupled in the cost function (17) because they are coupled in ProcessError(A, X). Consequently, standard convex optimization methods cannot be used to solve (16). Instead, the system identification problem will be solved using a heuristic that approximates (16).

Each column of MeasurementError$(X; o)$ takes the form,

$$x(k, w; o) - x_{k,w} \in \mathbb{R}^5, \tag{19}$$

where $x(k, w; o) \in \mathbb{R}^5_+$ is the composite data sample computed from the (time k, well w)-observation assuming death option o (2), and $x_{k,w} \in \mathbb{R}^5_+$ is the (time k, well w)-column of variable X.

Remark. $x(k, w; o)$ is known and computed using (2), while $x_{k,w}$ is a variable to be optimized using a program that approximates (16).

To solve (16) approximately, we used a standard algorithm called alternating minimization (AM). The algorithm reduces the value of the cost (17) in an iterative fashion by alternating the role of the optimization variable between A and X. Please see the references in the methods section of the main manuscript for examples of AM in the literature.

5.1 Preliminary Analysis

The values of (λ, μ, o) in (17) need to be chosen for each drug δ. In preliminary analysis, we examined many possible values, $(\lambda, \mu, o) \in \mathcal{S}^2_{\text{pre}} \times \{1, 2, 3\}$, where

$$\mathcal{S}_{\text{pre}} = \{1, \frac{1}{2}, \frac{1}{10}, \frac{1}{20}, \frac{1}{10^2}, \frac{1}{200}, \frac{1}{10^3}, \frac{1}{10^4}, \frac{1}{10^5}\}, \tag{20}$$

and allowed the death gains to be unequal. See $\mathcal{A}_{\text{pre}} \subset \mathbb{R}^{5\times 5}$ in Def. 2.2.

The data for each drug was split into a training set (∼2/3) and a test set (∼1/3). The test set contained only available samples to facilitate the calculation of cross-validation error. Composite training data for each death option o and composite test data for each death option o were computed (Sec. 4). For each $(\lambda, \mu, o) \in \mathcal{S}^2_{\text{pre}} \times \{1, 2, 3\}$, a dynamics matrix $A^{\text{tr}}_{\lambda,\mu,o} \in \mathcal{A}_{\text{pre}}$ was identified on the composite training data for death option o via alternating minimization with the cost (17) and the weights (λ, μ). Cross-validation error was computed to determine how well $A^{\text{tr}}_{\lambda,\mu,o}$ fit the composite test data for death option o.

Remark. The cross-validation error metric is $|\text{ProcessError}(A^{\text{tr}}_{\lambda,\mu,o}, X^{\text{te}}_o)|$. X^{te}_o is the (known) composite test data matrix for death option o. See (18) and Definition 5.1.

Cross-validation error versus (λ, μ) for each death option o is provided for each drug in Fig. 2-Fig. 5. Interestingly, the error changes substantially as λ varies but not as μ or o vary. In particular, each surface is relatively constant along the μ-axis at any fixed (λ, o). Further, at any fixed (λ, μ), the cross-validation error is similar among the three death options. So, λ is more important than μ or o, and the terms affected by λ in the cost function (17) depend on the dynamics matrix variable A, namely $|A|$ and $|\text{ProcessError}(A, X)|$, not on the death option o. The majority of terms in the dynamics matrix variable involve transition (1). Taken together, the results suggest that the data is generally insensitive to the distribution of death across the phenotypic states due to the more prominent role of phenotypic state transition.

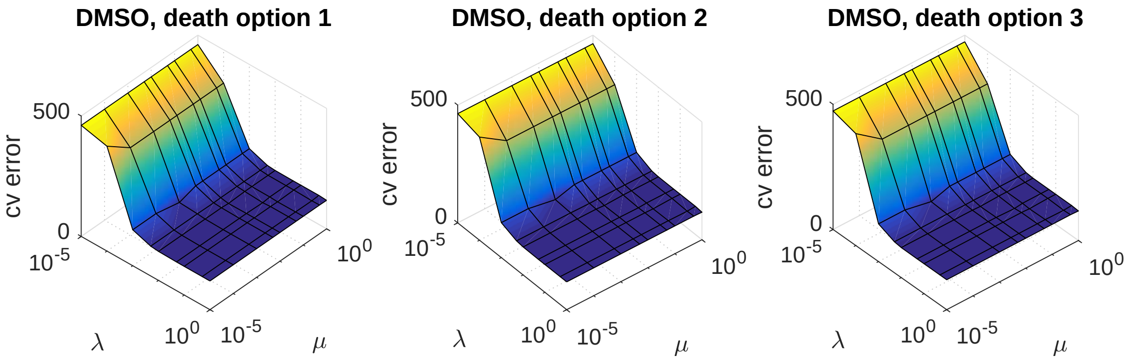

Figure 2: DMSO, Cross-validation error versus $(\lambda, \mu, o) \in \mathcal{S}_{\text{pre}}^2 \times \{1, 2, 3\}$.

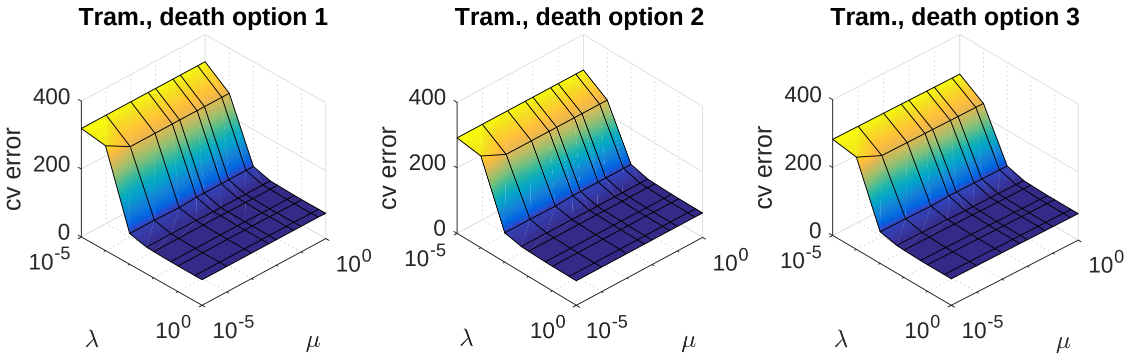

Figure 3: Trametinib, Cross-validation error versus $(\lambda, \mu, o) \in \mathcal{S}_{\text{pre}}^2 \times \{1, 2, 3\}$.

5.2 Using the Preliminary Analysis

Our approach to system identification appreciated the insights from the preliminary analysis to reduce computational cost and overfitting. We set the death option to evenly distributed death across the phenotypic states ($o = 2$) because the data did not display a strong preference for the more restrictive options. λ could take on several values associated with lower

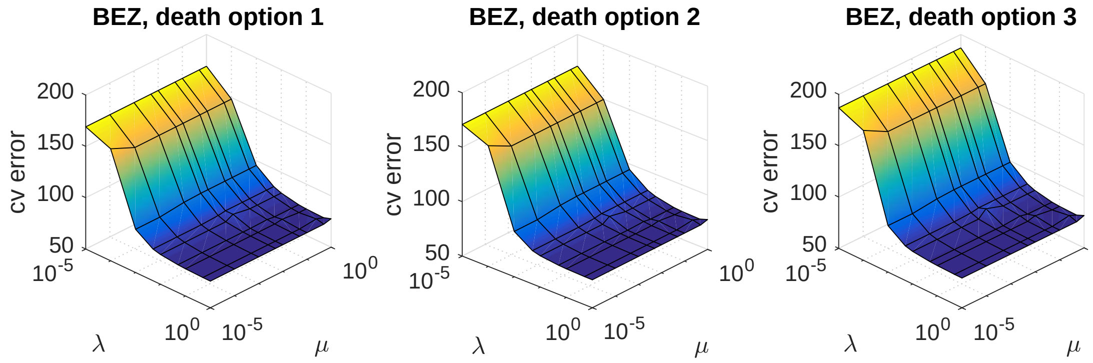

Figure 4: BEZ235, Cross-validation error versus $(\lambda, \mu, o) \in \mathcal{S}_{\text{pre}}^2 \times \{1, 2, 3\}$.

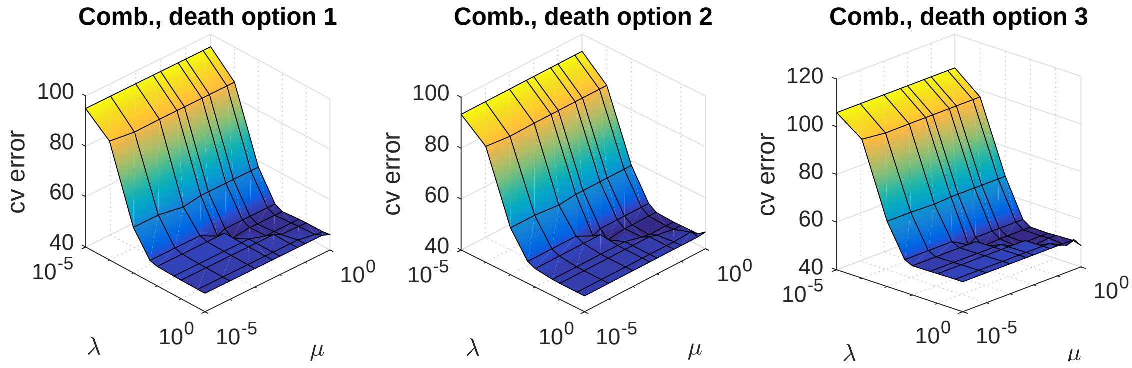

Figure 5: Trametinib+BEZ235, Cross-validation error versus $(\lambda, \mu, o) \in \mathcal{S}_{\text{pre}}^2 \times \{1, 2, 3\}$.

cross-validation error,

$$\mathcal{S} = \{1, \frac{1}{2}, \frac{1}{10}, \frac{1}{20}, \frac{1}{10^2}, \frac{1}{200}, \frac{1}{10^3}\}. \tag{21}$$

The value of μ was set to the value of λ because μ displayed a much lower degree of importance than λ. Assuming a close relationship between death rates and death distributions, we set the death gains equal among the phenotypic states; see Def. 2.1.

For each drug δ, cross-validation was performed for $\lambda \in \mathcal{S}$, $\mu = \lambda$, and $o = 2$, yielding the chosen weight λ_δ^*. Then, the optimized drug-specific dynamics and data matrices $(A_\delta^*, X_\delta^*) \in \mathcal{A} \times \mathbb{R}_+^{5 \times 105}$ were found via alternating minimization with the cost (17) and $(\lambda, \mu, o) = (\lambda_\delta^*, \lambda_\delta^*, 2)$.

6 Model Testing

For each drug δ, we examined how well the dynamics matrix A_δ^* fit to another data set. This test set consists of a phenotype time series and a death time series with measurements from 4 replicate wells every 12h over 6 time points. The two time series were merged together assuming evenly distributed death ($o = 2$), yielding a composite test data set. These test

samples were fitted to A_δ^*,

$$\hat{x}(k+1, w; \delta) = A_\delta^* \cdot x(k, w; \delta), \tag{22}$$

where x is the (time k, well w)-test sample and $\hat{x}$ is the prediction at the next time step. Shared initial conditions for x and $\hat{x}$ were assumed.

7 Uncertainty Analysis

For each drug δ, we studied the variation of the dynamics matrix A_δ^* in the presence of measurement error using the resampling residuals bootstrap proposed by Wu [6], a well-established algorithm for statistical inference. We present it briefly here. For each sample j, specify the value $s_j^\dagger$ of a discrete random variable,

$$S^\dagger = \begin{cases} 1 & \text{with probability } 1/2 \\ -1 & \text{with probability } 1/2 \end{cases}. \tag{23}$$

Using the value $s_j^\dagger$, compute the resample j,

$$x_j^\dagger = \hat{x}_j + (x_j - \hat{x}_j) \cdot s_j^\dagger, \tag{24}$$

where $\hat{x}_j \in \mathbb{R}$ is the fitted sample and $x_j \in \mathbb{R}$ is the measured sample. The resampling method (23) was proposed by Davidson and Flachaire [7]. The *fitted sample* is the entry of the optimized data $X_\delta^* \in \mathbb{R}^{5 \times 105}$ for cell type i, time k, and well w. The *measured sample* is element i of the cell type vector (2) computed with the (time k, well w)-sample under death option $o = 2$. The data-generating process (24) assumes that the measurement errors are homoskedastic (i.e., have constant variance) and are independent across cell types conditioned on time point and well index.

For each bootstrap iteration b, the residuals were resampled with the optimized data as the fitted samples and the composite data under death option $o = 2$ as the measured samples. The system identification problem was solved on the resampled data, yielding a bootstrapped model $A_\delta^b \in \mathcal{A}$. 95% confidence intervals were computed using the collection of bootstrapped models $\{A_\delta^b\}_{b=1}^{120}$. The procedure was completed for every drug δ.

References

[1] M. P. Chapman, T. T. Risom, A. Aswani, R. Dobbe, R. C. Sears, and C. J. Tomlin, "A model of phenotypic state dynamics initiates a promising approach to control heterogeneous malignant cell populations," in *Decision and Control (CDC), 2016 IEEE 55th Conference on.* IEEE, 2016, pp. 2481–2487.

[2] T. T. Risom, E. M. Langer, M. P. Chapman, J. Rantala, M. J. Alvarez, N. D. Kendsersky, C. R. Pelz, K. Johnson-Camacho, K. Chin, N. J. Wang, P. Spellman, A. Califano, C. J. Tomlin, J. W. Gray, and R. C. Sears, "Differentiation-state plasticity is a targetable resistance mechanism in basal-like breast cancer," *Nature Communications*, p. In press, 2018.

[3] S. Boyd and L. Vandenberghe, *Convex Optimization.* Cambridge University Press, 2004.

[4] T. Hastie, R. Tibshirani, and J. Friedman, *The Elements of Statistical Learning*, 2nd ed. Springer-Verlag, 2009.

[5] A. E. Hoerl and R. W. Kennard, "Ridge regression: Biased estimation for nonorthogonal problems," *Technometrics*, vol. 12, no. 1, pp. 55–67, 1970.

[6] C.-F. J. Wu, "Jackknife, bootstrap and other resampling methods in regression analysis," *The Annals of Statistics*, vol. 14, no. 4, pp. 1261–1295, 1986.

[7] R. Davidson and E. Flachaire, "The wild bootstrap, tamed at last," *Journal of Econometrics*, vol. 146, no. 1, pp. 162–169, 2008.
