# Supplementary material for: Modeling differentiation-state transitions linked to therapeutic escape in triple-negative breast cancer
Source: PLoS Comput Biol. 2019 Mar 11;15(3):e1006840. doi: 10.1371/journal.pcbi.1006840 (PMC6428348; doi:10.1371/journal.pcbi.1006840)

S2 Appendix: P-Values for Dynamics Parameters

For a given treatment condition, we conducted a two-sided one-sample sign test for each dynamics parameter using the samples obtained from the resampling residuals bootstrap algorithm (see Methods). This test assesses whether a collection of samples has a continuous distribution with median *m* against the alternative that the distribution does not have median *m* at the 5% significance level. We used the MATLAB function **signtest** and the following parameter-specific medians: no division ($\rho_i = m = 1$), no switching ($\rho_{ij} = m = 0$), and no death ($\rho_{iD} = m = 0$). A *small* p-value indicates that the null hypothesis of median *m* is rejected (i.e., a statistically significant difference is detected). The table below indicates that for most parameters the corresponding null hypothesis is rejected. Further, we have provided histograms of several collections of samples.

Remark. Before conducting each test, we added zero-mean Gaussian noise with standard deviation equal to 10^{-7} to the samples to ensure a continuous empirical distribution. Without additional noise, for example, the null hypothesis of median $m = 1$ was rejected ($p<0.001$) for ρ_1 of the Trametinib+BEZ235 condition, which is not a valid outcome since all the samples have values near 1 (see histogram to the right). With additional noise, however, the outcome of the hypothesis test ($p=0.65$) is consistent with the samples.

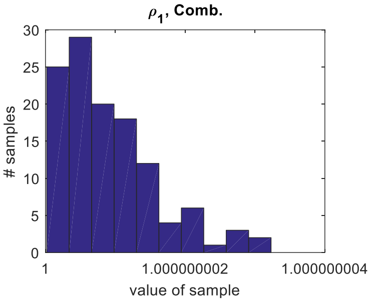

	DMSO	Trametinib	BEZ235	Trametinib+ BEZ235
$\rho_1 = \rho_2 = \rho_3 = \rho_4$	$p < 0.001$	$p < 0.001$	$p < 0.001$	$p = 0.65$
ρ_{12}	$p < 0.001$	$p < 0.001$	$p < 0.001$	$p < 0.001$
ρ_{13}	$p < 0.001$	$p < 0.001$	$p < 0.001$	$p < 0.001$
ρ_{14}	$p < 0.001$	$p < 0.001$	$p < 0.001$	$p < 0.001$
$\rho_{1D} = \rho_{2D} = \rho_{3D} = \rho_{4D}$	$p < 0.001$	$p < 0.001$	$p < 0.001$	$p < 0.001$
ρ_{21}	$p < 0.001$	$p < 0.001$	$p < 0.001$	$p < 0.001$
ρ_{23}	$p < 0.001$	$p < 0.001$	$p < 0.001$	$p = 0.52$
ρ_{24}	$p < 0.001$	$p < 0.001$	$p < 0.001$	$p < 0.001$
ρ_{31}	$p < 0.001$	$p < 0.001$	$p = 0.0024$	$p < 0.001$
ρ_{32}	$p < 0.001$	$p < 0.001$	$p < 0.001$	$p = 0.93$
ρ_{34}	$p < 0.001$	$p < 0.001$	$p < 0.001$	$p < 0.001$
ρ_{41}	$p < 0.001$	$p < 0.001$	$p = 0.78$	$p < 0.001$
ρ_{42}	$p < 0.001$	$p < 0.001$	$p < 0.001$	$p < 0.001$
ρ_{43}	$p < 0.001$	$p < 0.001$	$p < 0.001$	$p < 0.001$

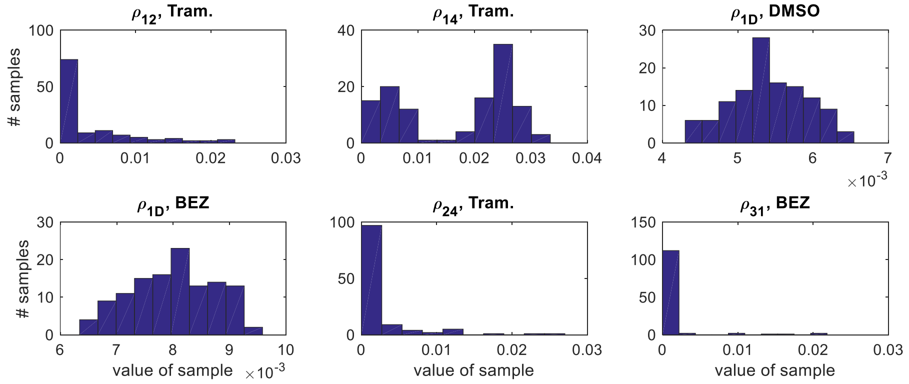

Supplement: S2 Appendix — This appendix provides the outcome of a two-sided one-sample sign test for each dynamics parameter. (PDF) [file pcbi.1006840.s008.pdf]
