# Supplementary material for: Modeling differentiation-state transitions linked to therapeutic escape in triple-negative breast cancer
Source: PLoS Comput Biol. 2019 Mar 11;15(3):e1006840. doi: 10.1371/journal.pcbi.1006840 (PMC6428348; doi:10.1371/journal.pcbi.1006840)

S3 Appendix: Evolution of the Dynamics Parameters during Alternating Minimization

The following figures show how the values of the dynamics parameters evolve during the iterative process of the alternating minimization algorithm, while the optimized dynamics matrix was being identified. In particular, the results indicate that the value of each dynamics parameter converges within numerical accuracy.

DMSO

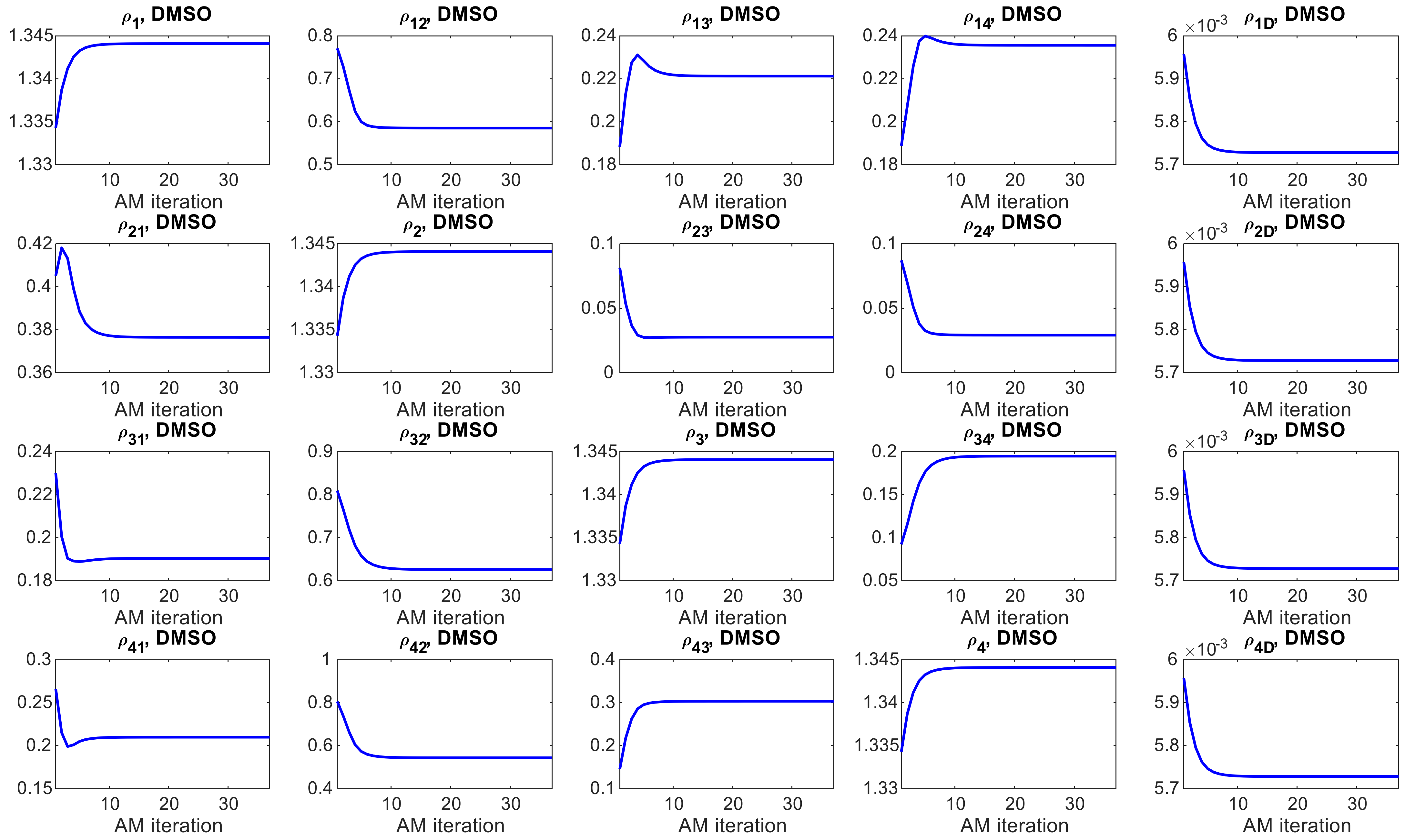

ρ1, DMSO
ρ12, DMSO
ρ13, DMSO
ρ14, DMSO
ρ1D, DMSO
ρ21, DMSO
ρ2, DMSO
ρ23, DMSO
ρ24, DMSO
ρ2D, DMSO
ρ31, DMSO
ρ32, DMSO
ρ3, DMSO
ρ34, DMSO
ρ3D, DMSO
ρ41, DMSO
ρ42, DMSO
ρ43, DMSO
ρ4, DMSO
ρ4D, DMSO
×10-3
AM iteration

Trametinib

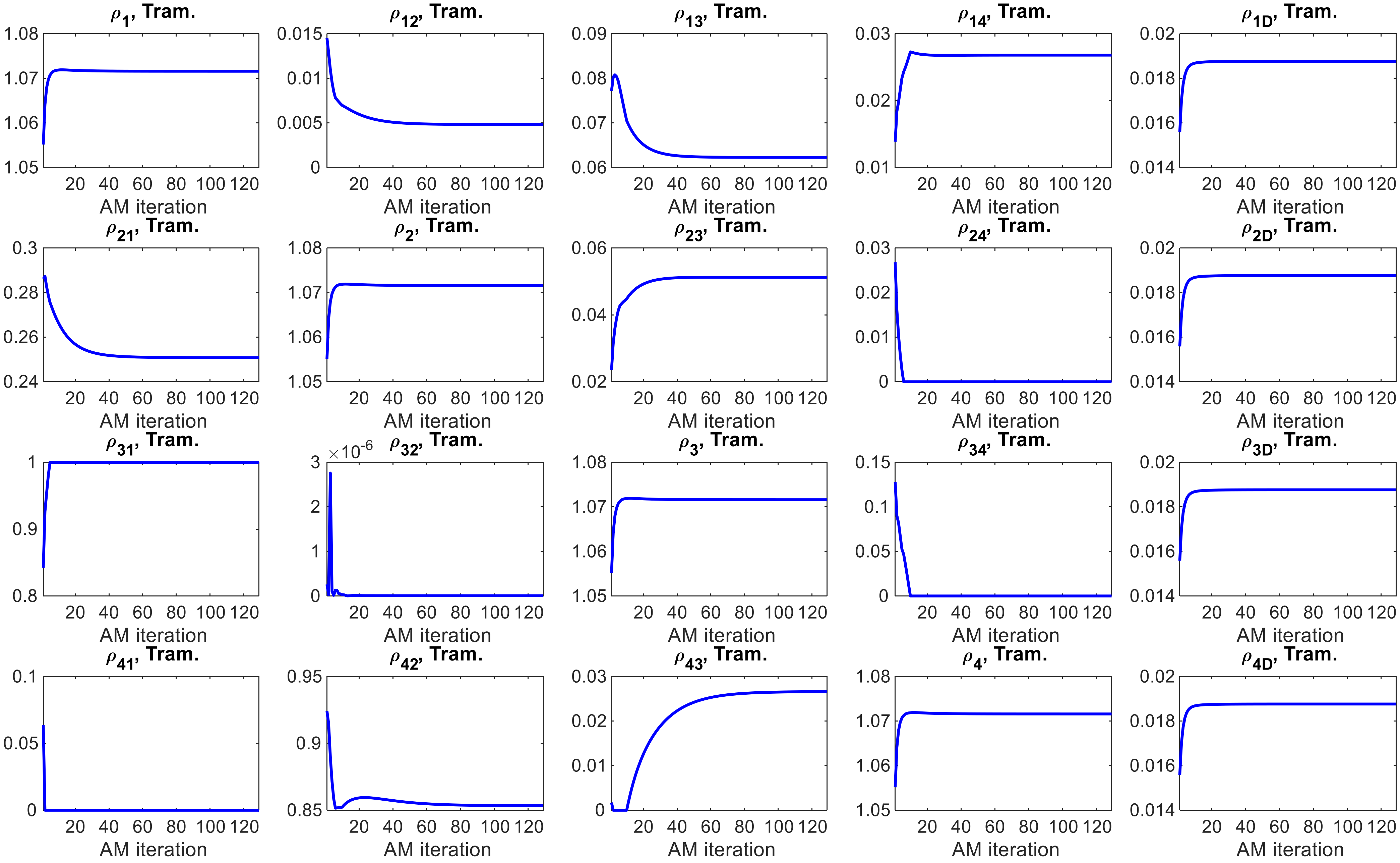

ρ1, Tram.
ρ12, Tram.
ρ13, Tram.
ρ14, Tram.
ρ1D, Tram.
ρ21, Tram.
ρ2, Tram.
ρ23, Tram.
ρ24, Tram.
ρ2D, Tram.
ρ31, Tram.
ρ32, Tram.
×10-6
ρ3, Tram.
ρ34, Tram.
ρ3D, Tram.
ρ41, Tram.
ρ42, Tram.
ρ43, Tram.
ρ4, Tram.
ρ4D, Tram.
20 40 60 80 100 120
AM iteration

BEZ235

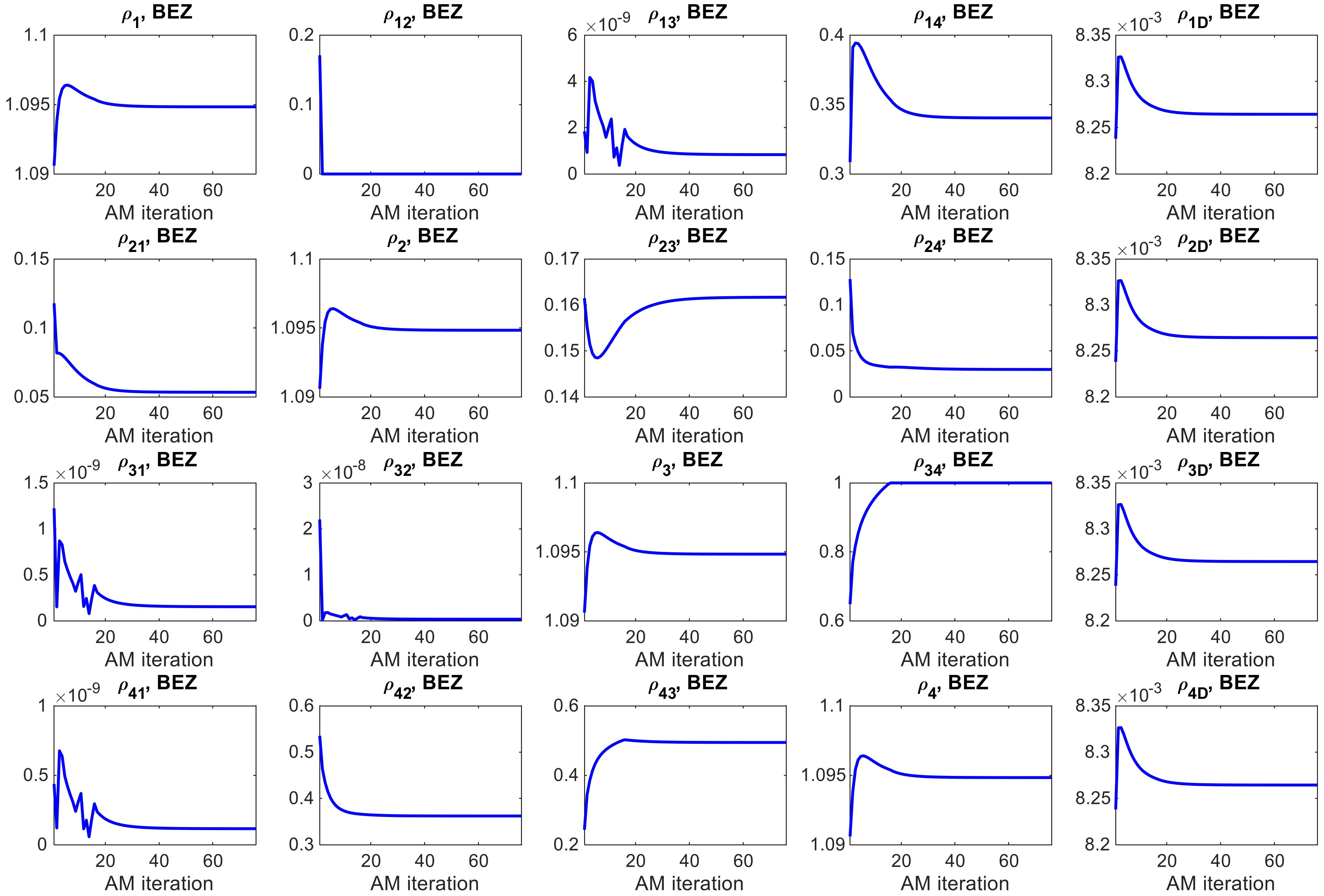

ρ1, BEZ
ρ12, BEZ
ρ13, BEZ
ρ14, BEZ
ρ1D, BEZ
ρ21, BEZ
ρ2, BEZ
ρ23, BEZ
ρ24, BEZ
ρ2D, BEZ
ρ31, BEZ
ρ32, BEZ
ρ3, BEZ
ρ34, BEZ
ρ3D, BEZ
ρ41, BEZ
ρ42, BEZ
ρ43, BEZ
ρ4, BEZ
ρ4D, BEZ
AM iteration

Trametinib+BEZ235

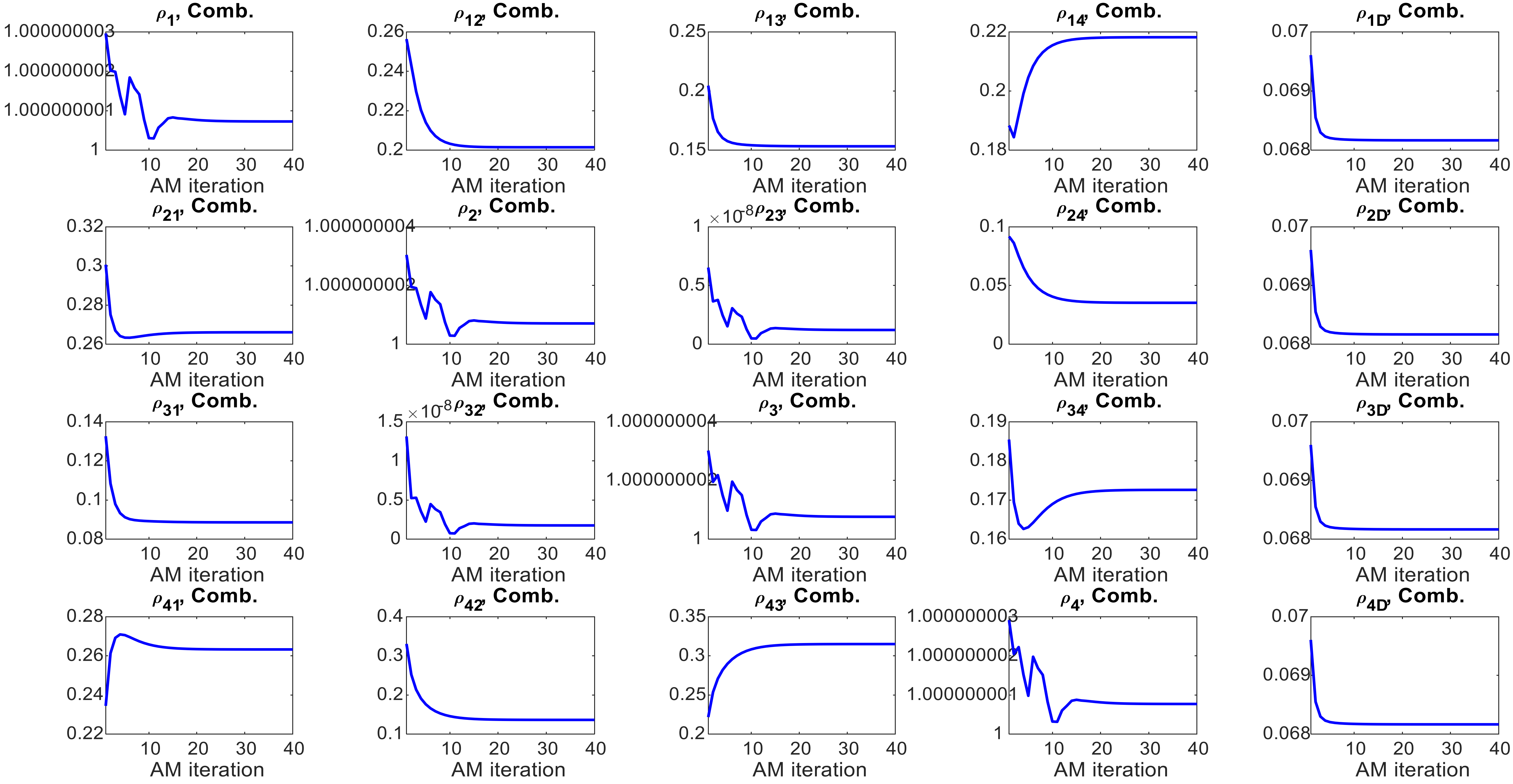

Supplement: S3 Appendix — This appendix shows how the values of the dynamics parameters evolve during the iterative process of the alternating minimization algorithm. (PDF) [file pcbi.1006840.s009.pdf]
