# Supplementary material for: Modeling differentiation-state transitions linked to therapeutic escape in triple-negative breast cancer
Source: PLoS Comput Biol. 2019 Mar 11;15(3):e1006840. doi: 10.1371/journal.pcbi.1006840 (PMC6428348; doi:10.1371/journal.pcbi.1006840)
Supplement: S4 Appendix — This appendix assesses the sensitivity of the dynamics matrix returned by the alternating minimization algorithm with respect to the initialization. (PDF) [file pcbi.1006840.s010.pdf]

S4 Appendix: Multi-Initialization System Identification

For each drug δ, to identify a dynamics matrix A_δ^*, recall that we set the data variable to a specific initial value $\hat{X}_\delta$ prior to starting the alternating minimization algorithm. Further recall that each column of $\hat{X}_\delta$ is a training data sample for a particular time point-well pair, or the sample mean of the available training data for the time point when training data for the time point-well pair was not available. Training data samples for all time point-well pairs were not available due to instrument errors that occurred during the cell line experiments (see Methods).

In a separate study (provided in this appendix), we assessed how different initial values of the data variable $\hat{Y}_\delta^{(j)}$ affected the dynamics matrix that was identified $A_\delta^{(j)}$. Each column of $\hat{Y}_\delta^{(j)}$ is a training data sample for a particular time point-well pair, or a *pseudorandom* sample drawn from a Gaussian distribution when training data for the time point-well pair was not available. (The mean and the covariance of the Gaussian distribution are the sample mean and the sample covariance of the available training data for the time point.)

The following tables show the difference between each pseudorandom initial value $\hat{Y}_\delta^{(j)}$ and the initial value used in the main paper $\hat{X}_\delta$, and the difference between the dynamics matrices that were identified using these initial values ($A_\delta^{(j)}$ and A_δ^*, respectively). Specifically, these dynamics matrices are similar in most cases, which justifies the initialization used in the main paper.

**δ = Trametinib+BEZ235**		
**Trial index j**	**Maximum absolute entry-wise difference between $\hat{Y}_\delta^{(j)}$ and $\hat{X}_\delta$**	**Maximum absolute entry-wise difference between $A_\delta^{(j)}$ and A_δ^***
1	131	1.19e-07
2	116	1.19e-07
3	152	4.09e-07
4	115	2.06e-08
5	87.4	4.40e-07
6	89.8	1.86e-07
7	134	1.23e-08
8	101	3.94e-07
9	90.1	4.03e-07
10	97.0	6.18e-08

**δ = DMSO**		
**Trial index j**	**Maximum absolute entry-wise difference between $\hat{Y}_\delta^{(j)}$ and $\hat{X}_\delta$**	**Maximum absolute entry-wise difference between $A_\delta^{(j)}$ and A_δ^***
1	215	0.0045
2	158	0.0124
3	441	0.0058
4	303	0.0073
5	327	0.0234
6	549	0.0049
7	337	0.0046
8	238	0.0070
9	292	0.0083
10	517	0.0178

δ = Trametinib		
**Trial index j**	**Maximum absolute entry-wise difference between $\hat{Y}_\delta^{(j)}$ and $\hat{X}_\delta$**	**Maximum absolute entry-wise difference between $A_\delta^{(j)}$ and A_δ^***
1	130	3.65e-07
2	169	9.07e-07
3	192	0.48
4	149	0.48
5	118	2.86e-07
6	245	8.72e-08
7	119	0.48
8	152	1.01e-06
9	144	1.04e-07
10	182	5.98e-07

Remark. The large differences between A_δ^* and $A_\delta^{(j)}$ for j = 3, 4, 7 (δ = Trametinib) arise from the bolded entries in the third column below.

$$A_\delta^{(3)} \approx A_\delta^{(4)} \approx A_\delta^{(7)} \approx \begin{bmatrix} 0.99 & 0.28 & \mathbf{0.52} & 0 & 0 \\ 0.0012 & 0.76 & 0 & 0.78 & 0 \\ 0.040 & 0.0077 & \mathbf{0.53} & 0.058 & 0 \\ 0.025 & 0 & 0 & 0.21 & 0 \\ 0.019 & 0.019 & 0.019 & 0.019 & 1 \end{bmatrix}$$

$$A_\delta^* \approx \begin{bmatrix} 0.96 & 0.25 & \mathbf{1} & 0 & 0 \\ 0.0048 & 0.75 & 0 & 0.85 & 0 \\ 0.062 & 0.051 & \mathbf{0.053} & 0.027 & 0 \\ 0.027 & 0 & 0 & 0.17 & 0 \\ 0.019 & 0.019 & 0.019 & 0.019 & 1 \end{bmatrix}$$

Note that the entry in the first row and third column is the dynamics parameter ρ_{31} (see S1 Appendix). Also note that the 95% confidence interval for ρ_{31} under Trametinib extends from 0.32 to 1 (Fig. 1 in this appendix). In trials 3, 4, and 7, $\rho_{31} \approx 0.52$, which is within this confidence interval. The study in this appendix and the uncertainty analysis in the main paper both indicate that ρ_{31} for Trametinib is not fully constrained by the (training) data. Further, A_δ^* and $A_\delta^{(j)}$ for j = 3, 4, 7 (δ = Trametinib) have similar cell division gains (sum the entries in the third column to compute ρ_3, see S1 Appendix). This outcome is consistent with the uncertainty analysis: the confidence interval for ρ_3 under Trametinib has a small range, meaning that ρ_3 is well-constrained by the data (Fig. 2 in this appendix).

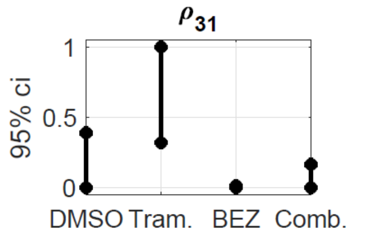

Fig. 1: 95% confidence intervals for ρ_{31}. Taken from uncertainty analysis figure in main paper.

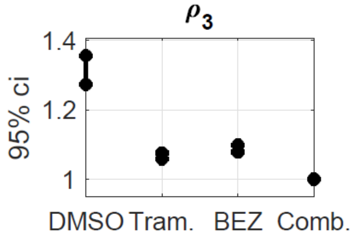

Fig. 2: 95% confidence intervals for the cell division gain. Taken from uncertainty analysis figure in main paper.

δ = BEZ235		
**Trial index j**	**Maximum absolute entry-wise difference between $\widehat{Y}_{\delta}^{(j)}$ and $\widehat{X}_{\delta}$**	**Maximum absolute entry-wise difference between $A_{\delta}^{(j)}$ and A_{δ}^{*}**
1	140	4.57e-07
2	141	1.20e-07
3	178	8.02e-07
4	121	1.32e-07
5	123	1.26e-06
6	126	4.27e-07
7	162	2.18e-06
8	135	4.63e-07
9	199	6.51e-07
10	188	4.88e-07
